# Dynamic Connectivity in Disk Graphs

**DOI:** 10.1007/s00454-023-00621-x

**Published:** 2024-01-03

**Authors:** Alexander Baumann, Haim Kaplan, Katharina Klost, Kristin Knorr, Wolfgang Mulzer, Liam Roditty, Paul Seiferth

**Affiliations:** 1 Institut für Informatik, Freie Universität Berlin, Berlin, Germany; 2 School of Computer Science, Tel Aviv University, Tel Aviv-Yafo, Israel; 3 Department of Computer Science, Bar Ilan University, Ramat Gan, Israel

**Keywords:** Disk graphs, Connectivity, Lower envelopes, Theory of computation →  Computational geometry, Theory of computation →  Data structures design and analysis, Mathematics of computing →  Paths and connectivity problems

## Abstract

Let $S \subseteq \mathbb {R}^2$ be a set of *n* *sites* in the plane, so that every site $s \in S$ has an *associated radius* $r_s > 0$. Let $\mathcal {D}(S)$ be the *disk intersection graph* defined by *S*, i.e., the graph with vertex set *S* and an edge between two distinct sites $s, t \in S$ if and only if the disks with centers *s*, *t* and radii $r_s$, $r_t$ intersect. Our goal is to design data structures that maintain the connectivity structure of $\mathcal {D}(S)$ as sites are inserted and/or deleted in *S*. First, we consider *unit disk graphs*, i.e., we fix $r_s = 1$, for all sites $s \in S$. For this case, we describe a data structure that has $O(\log ^2 n)$ amortized update time and $O(\log n/\log \log n)$ query time. Second, we look at disk graphs *with bounded radius ratio* $\Psi$, i.e., for all $s \in S$, we have $1 \le r_s \le \Psi$, for a parameter $\Psi$ that is known in advance. Here, we not only investigate the fully dynamic case, but also the incremental and the decremental scenario, where only insertions or only deletions of sites are allowed. In the fully dynamic case, we achieve amortized expected update time $O(\Psi \log ^{4} n)$ and query time $O(\log n/\log \log n)$. This improves the currently best update time by a factor of $\Psi$. In the incremental case, we achieve logarithmic dependency on $\Psi$, with a data structure that has $O(\alpha (n))$ amortized query time and $O(\log \Psi \log ^{4} n)$ amortized expected update time, where $\alpha (n)$ denotes the inverse Ackermann function. For the decremental setting, we first develop an efficient decremental *disk revealing* data structure: given two sets *R* and *B* of disks in the plane, we can delete disks from *B*, and upon each deletion, we receive a list of all disks in *R* that no longer intersect the union of *B*. Using this data structure, we get decremental data structures with a query time of $O(\log n/\log \log n)$ that supports deletions in $O(n\log \Psi \log ^{4} n)$ overall expected time for disk graphs with bounded radius ratio $\Psi$ and $O(n\log ^{5} n)$ overall expected time for disk graphs with arbitrary radii, assuming that the deletion sequence is oblivious of the internal random choices of the data structures.

## Introduction

Suppose we are given a simple, undirected graph *G*, and we would like to preprocess it so that we can determine efficiently if any two given vertices of *G* lie in the same connected component. If *G* is fixed, there is a simple solution: we perform a graph search in *G* (e.g., a BFS or a DFS [15]) to label the vertices of each connected component with a unique identifier. After that, we can answer a query in *O*(1) time by comparing the corresponding identifiers, with linear preprocessing time and space.

A much harder situation occurs if *G* changes over time. Now, the connectivity queries may be interleaved with operations that modify *G*. If *G* has a fixed set of *n* vertices and edges can only be inserted, the problem directly reduces to disjoint-set-union [37]. Using a standard disjoint-set-union structure [15], an optimal data structure that achieves $O(\alpha (n))$ amortized time for edge insertions and queries can be constructed, where $\alpha (n)$ is the inverse Ackermann function [15]. This reduction seems to be folklore, we explain the details in Theorem 2.1 below. If the vertex set is fixed, but edges can be both inserted and deleted, the data structure with the currently fastest update time is due to Huang et al. [24], which is based on a result by Thorup [40]. It has amortized expected update time $O(\log n(\log \log n)^2)$ and query time $O(\log n / \log \log \log n)$. Generally, there were several recent results in this area [24, 29, 41]. In our constructions, we will use a slightly older, but simpler data structure by Holm, Lichtenberg, and Thorup [23] with a memory optimization by Thorup [40]. We will refer to this data structure as the *HLT-structure*. It achieves $O(\log n / \log \log n)$ query time and $O(\log ^2 n)$ amortized update time. For the special case of planar graphs, Eppstein et al. [19] give a data structure with $O(\log n)$ amortized time for both queries and updates.

One way to model the case of a changing vertex set is the *dynamic subgraph connectivity* problem. Here, we have a known fixed graph *H* with *n* vertices and *m* edges, and *G* is a subgraph of *H* that changes dynamically by activating or deactivating the vertices of *H* (the edges of *G* are induced by the active vertex set). In this setting, there is a data structure with $O(m^{1/3} {{\,\textrm{polylog}\,}}n)$ query time and $O(m^{2/3}{{\,\textrm{polylog}\,}}n)$ amortized update time, developed by Chan et al. [13].

In this paper, we add a geometric twist: we study the dynamic connectivity problem on different variants of *disk intersection graphs*. Let $S \subset \mathbb {R}^2$ be a set of planar *point sites*, where each site $s \in S$ has an *associated radius* $r_s > 0$. The *disk intersection graph* (*disk graph*, for short) $\mathcal {D}(S)$ is the undirected graph with vertex set *S* that has an edge between any two distinct sites *s* and *t* if and only if the Euclidean distance between *s* and *t* is at most $r_s + r_t$. In other words, there is an edge between *s* and *t* if and only if the disks with centers *s* and *t* and with radius $r_s$ and $r_t$, respectively, intersect. Note that even though $\mathcal {D}(S)$ is fully described by the *n* sites and their associated radii, it might have $\Theta (n^2)$ edges. Thus, our goal is to find algorithms whose running time depends only on the number of sites and not on the number of edges. We consider three variants of disk graphs, characterized by the possible values for the associated radii. In the first variant, *unit disk graphs*, all radii are 1. In the second variant, *disk graphs of bounded radius ratio*, all radii must come from the interval $[1, \Psi ]$, where $\Psi$ is a parameter that is known in advance and that may depend on the number of sites *n*, i.e., it can be as large as $n^2, 2^n$, or even larger. In the third variant, *general disk graphs*, the radii can be arbitrary positive real numbers.

The static connectivity problem in disk graphs can be solved similarly as in general graphs: we compute the connected components and label the vertices of each connected component with the same unique identifier. The challenge for disk graphs lies in finding a quick way to perform the preprocessing step. For unit disk graphs, there are several methods to perform a BFS in worst-cast time $O(n \log n)$ (see, e.g., [30] and the references therein). For general disk graphs, Cabello and Jejcic [8] observed that a BFS can be performed with the help of an efficient weighted nearest neighbor structure. With this approach and the fastest available data structures, this leads to an algorithm with expected running time $O(n \log ^4 n)$ [27, 31].

We assume that *S* is *dynamic*, i.e., sites can be inserted and deleted over time. At each update, the edges incident to the modified site appear or disappear in $\mathcal {D}(S)$. An update can change up to $n-1$ edges in $\mathcal {D}(S)$, so simply storing $\mathcal {D}(S)$ in the HLT-structure could lead to potentially superlinear update times and might even be slower than recomputing the connectivity information from scratch.

For dynamic connectivity in general disk graphs, Chan et al. [13] give a data structure with $O(n^{1/7 + \varepsilon })$ query time and $O(n^{20/21 +\varepsilon })$ amortized update time, where $\varepsilon > 0$ is a constant that can be made arbitrarily small, but which must be fixed. As far as we know, this is currently still the best fully dynamic connectivity structure for general disk graphs. However, Chan et al. present their data structure as a special case of a more general setting, so there is hope that the specific geometry of disk graphs may allow for better running times.

Indeed, several results show that for certain disk graphs, one can achieve polylogarithmic update and query times. For unit disk graphs, Chan et al. [13] observe that there is a data structure with $O(\log ^{6} n)$ amortized update time and $O(\log n/\log \log n)$ query time.^1^ For bounded radius ratio, Kaplan et al. [27] show that there is a data structure with amortized expected update time $O(\Psi ^2 \log ^{4}n)$ and query time $O(\log n /\log \log n)$.^2^

Both results use the notion of a *proxy graph*, a sparse graph that models the connectivity of the original disk graph and that can be updated efficiently with suitable dynamic geometric data structures. The proxy graph is then stored in the HLT -structure, so the query procedure coincides with the one by HLT. The update operations involve a combination of updating the proxy graph with the help of geometric data structures and of modifying the edges in the HLT-structure.

**Table 1:** The state of the art after our work

	Update time	Query time	Space usage
Unit disks, fully dynamic			
Chan et al. [13]	$O(\log ^4 n)$ amortized	$O(\log n/\log \log n)$	$O(n \log ^2 n)$
Theorem 3.2	$O(\log ^2 n)$ amortized	$O(\log n / \log \log n)$	*O*(*n*)
$r_s \in [1,\Psi ]$, fully dynamic			
Kaplan et al. [27]	$O(\Psi ^2 \log ^{4}n)$ amortized expected	$O(\log n/\log \log n)$	$O(\Psi ^2 n \log n)$
Theorem 4.6	$O(\Psi \log ^{4} n)$ amortized expected	$O(\log n/\log \log n)$	$O(\Psi n \log n)$
$r_s \in [1,\Psi ]$, insertion only			
Theorem 6.8	$O(\log \Psi \log ^{4} n)$ amortized expected	$O(\alpha (n))$ amortized	$O(n \log \Psi \log n)$
$r_s \in [1,\Psi ]$, deletion only			
Theorem 6.7	$O( n\log \Psi \log ^4n)$ overall expected with oblivious adversary	$O(\log n/\log \log n)$	$O(n (\log n + \log \Psi ))$
General, fully dynamic			
Chan et al. [13]	$O(n^{1/7 + \varepsilon })$ amortized	$O(n^{20/21 +\varepsilon })$	$O(n^{5/3 + \varepsilon })$
General, deletion only			
Theorem 7.7	$O(n\log ^{5} n)$ overall expected with oblivious adversary	$O(\log n/\log \log n)$	$O(n \log ^2 n)$

Time bounds are per operation, unless noted otherwise. In the semi-dynamic cases where no explicit bounds are given the best known results coincide with the fully-dynamic case. The space requirements for the results of Chan et al. [13] are not stated explicitly in their paper, but they were derived according to our understanding of their method

**Our results.** For unit disk graphs, we significantly improve the observation of Chan et al. [13]: with a direct approach utilizing a grid-based proxy graph and dynamic data structures for lower envelopes, we obtain $O(\log ^2 n)$ amortized update and $O(\log n / \log \log n)$ query time (Theorem 3.2).

For bounded radius ratio, we give a data structure that improves the update time in Theorem 4.6. Specifically, we achieve amortized expected update time $O(\Psi \log ^{4} n)$ and query time $O(\log n/\log \log n)$. Compared to the previous data structure of Kaplan et al. [27], this improves the factor in the update time from $\Psi ^2$ to $\Psi$.

We also provide partial results that push the dependency on $\Psi$ from linear to logarithmic. For this, we consider the *semi-dynamic* setting, in which only insertions (*incremental*) or only deletions (*decremental*) are allowed. In both semi-dynamic settings we use quadtrees and assign the input disks to certain sets defined by the cells of the quadtree. Similar approaches have been used before [7, 25, 26, 32, 33]. In the incremental setting, we use a dynamic additively weighted Voronoi diagram to obtain a data structure with $O(\alpha (n))$ amortized query time and $O(\log \Psi \log ^{4} n)$ amortized expected update time, see Theorem 6.8.

In the decremental setting, a main challenge is to identify those edges in $\mathcal {D}(S)$ that were incident to a freshly removed site and whose removal changes the connectivity in $\mathcal {D}(S)$. To address this, we first develop a data structure for a related dynamic geometric problem which might be of independent interest. In this problem, we have two sets *R* and *B* of disks in the plane, such that the disks in *R* can be both inserted and deleted, while the disks in *B* can only be deleted. We would like to maintain *R* and *B* in a data structure such that whenever we delete a disk *b* from *B*, we receive a list of all the disks in the current set *R* that intersect the disk *b* but no remaining disk in $B \setminus \{b\}$. We say that these are the disks in *R* that are *revealed* by the deletion of *b*. If *B* initially contains *n* disks, we can process a sequence of *m* updates to *R* and *k* deletions from *B* in expected time $O(n \log ^2 n + m \log ^4 n + k \log ^4 n)$, assuming an oblivious adversary. We call this data structure the *disk reveal data structure* (RDS ) and it can be found in Theorem 5.7.

The RDS plays a crucial part in developing decremental connectivity structures for disk graphs of bounded radius ratio and for general disk graphs. For both cases, we obtain data structures with $O(\log n/\log \log n)$ query time. The total expected time is $O(n\log \Psi \log ^{4} n )$ for bounded radius ratio (Theorem 6.7) and $O(n\log ^{5}n )$ for the general case (Theorem 7.7), assuming an oblivious adversary for both data structures. Our contributions and the current state of the art are summarized in Table 1. A simplified version of the decremental data structure for general disk graphs also implies a static connectivity data structure with $O(n\log ^2n)$ preprocessing and $O(1)$ query time (Lemma 7.8).

## Preliminaries

After formally defining our geometric setting, we briefly recall some basic notions and structures that will be relevant throughout the paper.

### Problem Setting

Let $S\subset \mathbb {R}^2$ be a set of *n* *sites* in the plane. Every $s \in S$ has an *associated radius* $r_s \in \mathbb {R}$, $r_s > 0$, and it defines a closed disk $D_s$ with center *s* and radius $r_s$. The *disk graph* $\mathcal {D}(S)$ of *S* is the graph with vertex set *S* and an undirected edge *st* if and only if $\Vert st\Vert \le r_s+r_t$. Here, $\Vert st\Vert$ denotes the Euclidean distance between *s* and *t*. Equivalently, there is an undirected edge *st* if and only if the disks $D_s$ and $D_t$ intersect. Two sites *s*, *t* are *connected* in $\mathcal {D}(S)$ if and only if $\mathcal {D}(S)$ contains a path between *s* and *t*.

We consider three types of disk graphs: *unit disk graphs*, *disk graphs of bounded radius ratio*, and *general disk graphs*. In unit disk graphs, we have $r_s = 1$, for all $s \in S$. In disk graphs of bounded radius ratio, we require that $r_s \in [1, \Psi ]$, for all $s \in S$, where $\Psi$ is some fixed parameter. The time bounds now may depend on $\Psi$. Note that $\Psi$ in turn may depend on *n*, i.e., be $n^2, 2^n$, or even larger. For simplicity, we assume that $\Psi$ is known to the data structure in advance. However, we believe that even if $\Psi$ is not previously known, most of our approaches can be adapted to achieve the same running times with regard to the maximum radius ratio over the life span of the data structure. In general disk graphs, there are no additional restrictions on the radii, and the running times depend only on *n*.

In all three scenarios, the site set *S* is *dynamic*, i.e., it may change over time. Our goal is to maintain *S* while allowing *connectivity queries* of the following form: *given two sites* $s, t \in S$, *are they connected in the current disk graph* $\mathcal {D}(S)$? Depending on the exact nature of how *S* can change, we distinguish between the *incremental*, the *decremental*, and the *fully dynamic* setting. In the incremental setting, we start with $S = \emptyset$, and we can only add new sites to *S*. In the decremental setting, we start with a given set *S* of *n* sites that may be subject to preprocessing, and we allow only deletions from *S*. In the fully dynamic case, we start with $S = \emptyset$, and sites can be inserted and deleted arbitrarily. In all three settings, the updates can be interleaved with the queries in any possible way.

### Data Structures for Edge Updates

We rely on existing data structures that support connectivity queries and edge updates on general graphs. In the incremental case, the problem reduces to disjoint-set-union [15]. The construction seems to be folklore (see e.g.@ Holm et al. [23], Reif [37]), we state it in the following theorem, and provide a proof for completeness.

#### Theorem 2.1

Starting from the empty graph, there is a deterministic data structure for incremental dynamic connectivity such that an isolated vertex can be added in *O*(1) time, an edge between two existing vertices can be added in $O(\alpha (n))$ amortized time, and a connectivity query takes $O(\alpha (n))$ amortized time, where *n* is the total number of inserted vertices so far and $\alpha (n)$ denotes the inverse Ackermann function.

#### Proof

This is a direct application of a disjoint-set-union structure with the operations Make-Set, Union, and Find [15]. The vertices of *G* correspond to the elements of the sets. An insertion of an isolated vertex becomes a Make-Set operation and takes *O*(1) time. To determine if two vertices are connected, we perform a Find operation on the corresponding elements, and we return *yes* if they lie in the same set. This has amortized running time $O(\alpha (n))$. To insert an edge *uv*, we first find the sets that contain *u* and *v*, and then we perform a Union operation on these sets. This takes $O(\alpha (n))$ amortized time. $\square$

For the fully dynamic case, we use the following result of Holm et al. [23]. We refer to this data structure as the *HLT-structure*.

#### Theorem 2.2

(Holm et al.  [23, Thm. 3]) Let *G* be a graph with *n* vertices and initially empty edge set. There is a deterministic fully dynamic data structure so that edge updates in *G* take amortized time $O(\log ^2 n)$ and connectivity queries take worst-case time $O(\log n/\log \log n)$.

Theorem 2.2 assumes that *n* is fixed. However, we can easily support vertex insertions and deletions within the same amortized time bounds, by rebuilding the data structure whenever the number of vertices changes by a factor of 2. Thorup presented a variant of Theorem 2.2 that uses *O*(*m*) space, where *m* is the number of edges that are currently stored in the data structure [40].

It should also be mentioned that both Theorem 2.1 and Theorem 2.2 can easily be extended to not only answer connectivity queries but also to maintain the number of connected components. As both data structures require some form of explicit operation when merging or splitting a connected component, a counter for the number of current components can be maintained and returned in *O*(1) time. This directly implies that all except one of our disk connectivity data structures can be extended to also support those queries in *O*(1) time. This is not directly clear for the data structure of Theorem 4.6, as this data structure does not maintain the full connectivity internally for an improved update time.

### The Hierarchical Grid and Quadtrees

**The hierarchical grid**

**Fig. 1:**
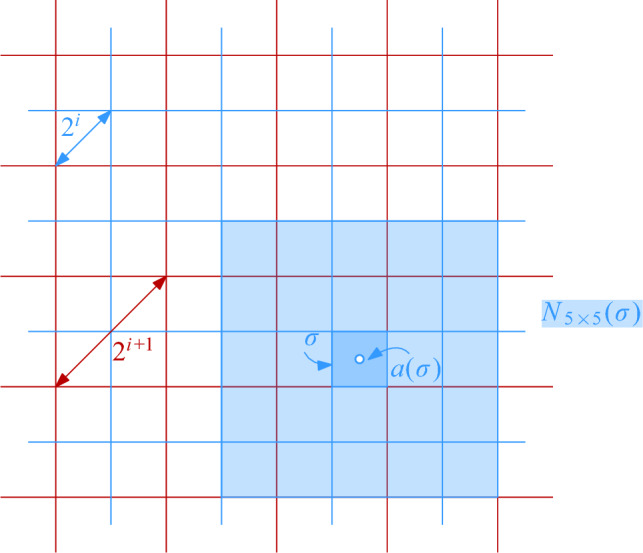
Two levels of the hierarchical grid, a cell $\sigma$ at level *i*, its center $a(\sigma )$, and the $(5 \times 5)$-neighborhood of $\sigma$

To structure our set of sites, we will make frequent use of the hierarchical grid and quadtrees. Let $i \ge 0$. A *cell* $\sigma$ *of level* *i* is an axis-parallel square with diameter $2^i$. On the boundary, the cell $\sigma$ contains the right edge without its bottom endpoint and the upper edge without its left endpoint, but none of the remaining points.^3^ The *grid* $\mathcal {G}_i$ is the set of all cells of level *i* such that $\mathcal {G}_i$ contains the cell with the origin as the upper right corner; the cells in $\mathcal {G}_i$ cover the plane; and the closures of any two cells in $\mathcal {G}_i$ are either disjoint or share exactly one corner or a complete boundary edge. Note that every point in the plane lies in exactly one cell of $\mathcal {G}_i$. The *hierarchical grid* $\mathcal {G}$ is then defined as $\mathcal {G}= \bigcup _{i = 0}^{\infty } \mathcal {G}_i$. For any cell $\sigma \in \mathcal {G}$, we denote by $|\sigma |$ its diameter and by $a(\sigma )$ its center. We say that $\mathcal {G}_i$ is the *grid at level i* in $\mathcal {G}$. We assume that given a point $p \in \mathbb {R}^2$ and a level $i \ge 0$, we can find (the coordinates of) the cell of $\mathcal {G}_i$ that contains *p* in *O*(1) time. Furthermore, for a cell $\sigma \in \mathcal {G}_i$ and odd $k \in {\mathbb {N}}$, the $(k\times k)$-*neighborhood* of $\sigma$, $N_{k\times k}(\sigma )$, is the set of $k^2$ cells in $\mathcal {G}_i$ that contains $\sigma$ and all cells that can be reached from $\sigma$ by crossing at most $(k-1)/2$ vertical and at most $(k-1)/2$ horizontal cell boundaries. See Fig. 1 for an illustration.

**Quadtrees**

Let $\mathcal {C} \subset \mathcal {G}$ be a finite set of cells. The *quadtree* $\mathcal {T}$ for $\mathcal {C}$ is a rooted 4-ary tree whose nodes are cells from $\mathcal {G}$. The root of $\mathcal {T}$ is the smallest cell $\rho$ in $\mathcal {G}$ that contains all the cells of $\mathcal {C}$. Starting from $\rho$, we expand the nodes of $\mathcal {T}$ as follows: if a cell $\sigma$ in $\mathcal {T}$ with level $i \ge 1$, properly contains at least one cell of $\mathcal {C}$, then $\sigma$ obtains four children, namely the cells $\tau _j$ with level $i-1$ and $\tau _j \subseteq \sigma$. If a cell $\sigma$ in $\mathcal {T}$ does not properly contain a cell of $\mathcal {C}$, it is not expanded any further, and it becomes a leaf of $\mathcal {T}$. Typically, we do not explicitly distinguish between a cell $\sigma$ and its associated node in $\mathcal {T}$. A quadtree $\mathcal {T}$ on a given set of *n* cells can be constructed in $O(n\log |\rho | )$ time, where $\rho$ is the root of $\mathcal {T}$.^4^

### Maximal Bichromatic Matchings

We make frequent use of a data structure that dynamically maintains a *maximal bichromatic matching* (MBM) between two sets of disks. Let $R \subseteq S$ and $B \subseteq S$ be two disjoint non-empty sets of sites, and let $(R \times B) \cap \mathcal {D}(S)$ be the bipartite graph on *R* and *B* that consists of all edges of $\mathcal {D}(S)$ with one vertex in *R* and one vertex in *B*. An MBM between *R* and *B* is a maximal set of vertex-disjoint edges in $(R \times B) \cap \mathcal {D}(S)$.

We show how to maintain a dynamic MBM as sites are inserted or deleted in *R* and in *B*. For this, we need a dynamic data structure for *disk unions*: let $T \subseteq S$ be a set of sites. A *disk union structure* dynamically maintains *T* as sites are inserted or deleted, while supporting the following query: given a site $s \in S$, report an arbitrary site $t \in T$ such that $D_s$ and $D_t$ intersect, or indicate that no such *t* exists. Given a disk union structure, we can maintain an MBM with only constant overhead:

#### Lemma 2.3

(Kaplan et al.  [27, Lem. 9.10]) Let $R,B \subseteq S$ be two disjoint sets with a total of at most *n* sites. Suppose we have a dynamic data structure for disk unions in *R* and *B* that has update time *U*(*m*), query time *Q*(*m*), and space requirement *S*(*m*), for *m* elements. Then, there exists a dynamic data structure that maintains an MBM for *R* and *B* with $O(U(n) + Q(n) + \log n)$ update time, using $O(S(n) + n)$ space.

#### Proof

We store MBM *M* as a red-black tree [15]. Each edge of the matching is stored twice, using as keys both of its vertices, and the search order is any sensible order. We use two disk union data structures $D_R$ and $D_B$ for the sites from *R* and *B* that currently do not appear as an endpoint in *M*. The invariant is that *M* contains a matching between *R* and *B*, and that the disks in $D_R$ and $D_B$ are pairwise disjoint. This implies that *M* is maximal.

To insert a new site *r* into *R*, we perform a query with *r* in $D_B$. If this query reports a site $b \in B$ such that $D_r$ and $D_b$ intersect, we add the edge *rb* to *M*, and we delete *b* from $D_B$. Otherwise, we insert *r* into $D_R$. To delete a site *r* from *R*, there are two cases: first, if *r* is unmatched, we simply remove *r* from $D_R$. Second, if *r* is matched, say to the site $b \in B$, we proceed as follows: we remove the edge *rb* from *M* and we query $D_R$ with *b*, looking for a new partner for *b* in *R*. If we find a new partner $r'$ for *b*, we delete $r'$ from $D_R$, and we add the edge $r'b$ to *M*. Otherwise, we insert *b* into $D_B$. Updates to *B* are analogous. Thus, an update to the MBM requires *O*(1) insertions, deletions, or queries to $D_R$ or $D_B$ and *O*(1) queries to *M*, and the lemma follows. $\square$

We use two ways to implement the dynamic structure for disk unions, based on two different underlying data structures. For the general case, we rely on an *additively-weighted nearest neighbor data structure* (AWNN). An AWNN stores a set $P \subset \mathbb {R}^2$ of *n* points in the plane, such that every point $p \in P$ is assigned a *weight* $w_p \in \mathbb {R}$. The AWNN supports *additively-weighted nearest neighbor queries*: given a point $q\in \mathbb {R}^2$, find the point $p \in P$ that minimizes the additively weighted Euclidean distance $\Vert pq \Vert + w_p$ to *q*. Kaplan et al. [27], with the improved construction of shallow cuttings by Liu [31], show the following result:

#### Lemma 2.4

(Kaplan et al.  [27, Thm. 8.3, Sect. 9], Liu [31, Corr. 4.3]) There is a fully dynamic AWNN data structure that allows insertions in $O(\log ^2 n)$ amortized expected time and deletions in $O(\log ^{4} n)$ amortized expected time. Furthermore, a query takes $O(\log ^2 n)$ worst case time. The data structure requires $O(n \log n)$ space.

Lemma 2.4 can be used directly to implement a dynamic disk union structure: we assign to each site $t \in T$ the weight $w_t = -r_t$. To check if a disk $D_s$ intersects a disk in *T*, we query the AWNN structure with *s*. Let $t \in T$ be the resulting additively-weighted nearest neighbor. We check if the disks $D_s$ and $D_t$ intersect. If so, we return *t*, otherwise, we report that no such intersection exists. Combining Lemmas 2.3 and 2.4, we get:

#### Lemma 2.5

Let $R,B \subseteq S$ be two disjoint sets with a total of at most *n* sites. Then, there exists a dynamic data structure that maintains an MBM for *R* and *B* with $O(\log ^{4} n)$ amortized expected update time, using $O(n \log n)$ space.

**Fig. 2:**
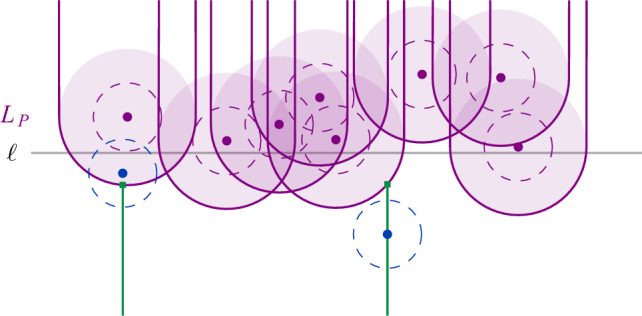
The set $L_P$ induced by *P*. The unit disks are drawn dashed. If a site $b \in B$ lies above the lower envelope, the unit disks intersect

Next, we consider the more restricted case that *S* contains only unit disks, and that the sites in *R* and *B* are separated by a vertical or horizontal line $\ell$ that is known in advance. In this case, we can implement a disk union structure using a *dynamic lower envelope* (DLE) structure for pseudolines. Indeed, suppose we have a dynamic set *T* of sites with $r_t = 1$, for all $t \in T$. Assume further that we are given a vertical or horizontal line $\ell$ such that all query sites *s* have $r_s = 1$ and are separated from *T* by $\ell$. We rotate and translate everything such that $\ell$ is the *x*-axis and all sites in *T* have positive *y*-coordinate. We consider the set $U_T$ of disks with radius 2 and centers in *T* (see Fig. 2). Then, a site in *s* that lies below $\ell$ and has $r_s = 1$ intersects some disk in *T* if and only if it is contained in the union of the disks in $U_T$. To detect this, we maintain the lower envelope of $U_T$, with a DLE-structure for pseudolines.

This is defined as follows: let *L* be a set of *pseudolines* in the plane, i.e., each element of *L* is a simple continuous *x*-monotone curve, and any two distinct curves in *L* cross in exactly one point (and do not meet anywhere else). The *lower envelope* of *L* is the pointwise minimum of the graphs of the curves in *L*. Combinatorially, it consists of a sequence of *pseudoline segments*, such that each pseudoline contributes at most one segment. The goal is to maintain a representation of this lower envelope efficiently as pseudolines are inserted and deleted in *L*. At the same time, the structure should support *vertical ray shooting queries*: given a query $q \in \mathbb {R}$, report the pseudoline(s) for the segment(s) of the current lower envelope that intersect the vertical line with *x*-coordinate *q*. Overmars and van Leeuwen show how to maintain the DLE of a set of lines with update time $O(\log ^2 n)$ such that *vertical ray shooting* queries take $O(\log n)$ time [35]. This was extended to pseudolines by Agarwal et al. [1]:

#### Lemma 2.6

(Agarwal et al. [1]) We can maintain the DLE of a set of pseudolines with $O(\log ^2 n)$ worst-case update time and $O(\log n)$ worst-case time for vertical ray shooting queries. Here, *n* denotes the maximum number of pseudolines in the structure. The data structure uses *O*(*n*) space.

Now, consider the following set $L_T$ of pseudolines: for each disk *D* of $U_T$, take the arc that defines the lower part of the boundary of *D* and extend both ends upward to $\infty$, with a very high slope. By maintaining $L_T$, we can implement a disk union structure for our special case with worst-case update time $O(\log ^2 n)$ and worst-case query time $O(\log n)$. Combining with Lemmas 2.3 and 2.6, we get:

#### Lemma 2.7

Suppose that $r_s = 1$, for all sites $s \in S$. Let $R,B \subseteq S$ be two disjoint sets with a total of at most *n* sites, such that there is a known vertical or horizontal line that separates *R* and *B*. Then, there exists a dynamic data structure that maintains an MBM for *R* and *B* with $O(\log ^{2} n)$ worst-case update time, using *O*(*n*) space.

**Remark.** By now, there are several improvements of the structure of Overmars and van Leeuwen [6, 10, 28], culminating in an optimal $O(\log n)$ amortized update time due to Brodal and Jacob [6]. There is solid evidence [22] that the improvement of Kaplan et al. [28] carries over to the pseudoline setting, giving a better $O(\log n\log \log n)$ amortized update time with $O(\log n)$ query time. However, there is no formal presentation of these arguments yet, so we refrain from formally stating the result. The structure by Brodal and Jacob [6] is very involved, and we were not able to verify if it carries over to the setting of pseudolines. This poses an interesting challenge for further investigation.

### Computational Model

Our algorithms use quadtrees and hierarchical grids. Even though this is a common practice in the computational geometry literature (see, e.g., Har-Peled’s book [21] for many examples of such algorithms), we should be aware that this creates some issues: in order to use hierarchical grids of arbitrary resolution, we typically need to determine which grid cell of a certain diameter contains a given point. To do this efficiently, we need to extend the standard computational model in computational geometry, the real RAM [36], by an additional *floor function* that rounds a given real number down to the next integer [21]. Even though this floor function is natural—and implemented in many real-world computers—it is problematic in the context of the real RAM: the floor function together with the unbounded precision of the real RAM provide us with a very powerful model of computation that can even solve PSPACE-complete problems in polynomial time (see, e.g., Mitchell and Mulzer [34] and the references therein for further discussion of this issue). Thus, when using this model, we should be careful to use the floor function in a “reasonable” way. Typically, it is considered reasonable to stipulate an operation that allows us to find the cell of a given level of the hierarchical grid that contains a given input point in constant time [21]. In the main part of this paper, we will follow this approach, because it will lead to a clearer description of the algorithms. Note that this is mainly an issue for insertion operations in the semi-dynamic incremental and the fully dynamic setting, because if the points are given in advance (as in the decremental setting), we have time to preprocess them to find the associated grid cells. Furthermore, for the query operations, we can store the associated grid cell for each point in the data structure with its satellite data, so that we do not need to determine this cell with the help of the floor function during the query.

However, it typically turns out that the use of the floor-function is not strictly necessary. The price we need to pay for this is that the grid cells are no longer perfectly aligned, which makes the algorithms a bit more messy. However, these issues can typically be dealt with no or little overhead (see, e.g., [7, 32] for examples). This is also true for our algorithms, and we give the details in the Appendix, in order to satisfy the curious reader while keeping the main text free from additional complications.

### The Role of Randomness

Most of our algorithms use randomness, and some of the running time guarantees hold in expectation only. We would like to emphasize that the expectation is over the randomness in the algorithms only, and they hold in the worst case over all possible query sequences. However, sometimes, in particular in the context of the disk revealing structure, we will need the *obliviousness assumption*. This assumption says that the query sequence must be *oblivious* to the random choices of the algorithm and may not adapt to the state of the data structure. This is a typical assumption in the analysis of data structures and is used, e.g., in the analysis of hashing-based structures. However, we should mention that in recent years a lot of effort has been put into designing randomized data structures that do not rely on the obliviousness assumption and that it is an intriguing open problem to obtain a disk revealing structure that does not need this assumption.

## Unit Disk Graphs

**Fig. 3:**
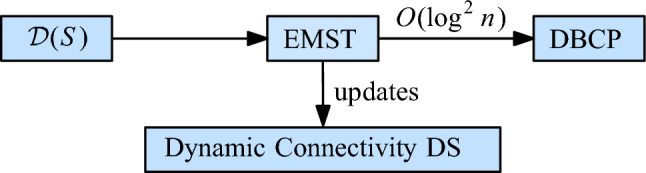
A solution for unit disks with $O(\log ^{6} n)$ update time

We first consider the case of unit disk graphs. As mentioned in the introduction, this problem was already addressed by Chan et al. [13]. They observed how to combine several known results into a data structure for connectivity queries in fully dynamic unit disk graphs with amortized update time $O(\log ^{6}n)$ and query time $O(\log n/\log \log n)$. We briefly sketch their approach (see Fig. 3): let *T* be the Euclidean minimum spanning tree (EMST) of *S*. If we remove from *T* all edges with length more than 2, the resulting forest *F* is spanning for $\mathcal {D}(S)$. Thus, to maintain the components of $\mathcal {D}(S)$, it suffices to maintain the components of *F*. For this, we use an HLT-structure $\mathcal {H}$. The geometric properties of the EMST ensure that inserting or deleting a site from *S* modifies *O*(1) edges in *T*. Thus, if we can efficiently find the set *E* of edges in *T* that change during an update, we can maintain the components of *F* through *O*(1) updates to $\mathcal {H}$, by considering all edges in *E* of length at most 2. To find *E*, we need to dynamically maintain the EMST *T* of *S*. For this, there is a technique of Agarwal et al. that reduces the problem to several instances of the *dynamic bichromatic closest pair problem* (DBCP), with an overhead of $O(\log ^2 n)$ in the update time [2]. Recently, Chan [12] showed how to solve the DBCP-problem with a modified dynamic nearest neighbor (DNN) structure, in $O(\log ^4 n)$ amortized update time. These two results together allow us to maintain the EMST dynamically with an amortized update time of $O(\log ^6 n)$.^5^ We can use $\mathcal {H}$ for queries in $O(\log n/\log \log n)$ time.

**Fig. 4:**
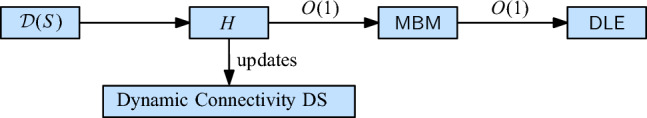
The structure of our data structure for unit disks

To improve over this result, we replace the EMST by a simpler graph that still captures the connectivity of $\mathcal {D}(S)$. We also replace the DNN structure by a suitable maximal bichromatic matching (MBM) structure that is based on dynamic lower envelopes (Lemma 2.7). These two changes improve the amortized update time to $O(\log ^2 n)$, without affecting the query time. Figure 4 shows the structure of our technique.

First, we define a *proxy graph* *H* that represents the connectivity of $\mathcal {D}(S)$. This proxy graph groups close unit disks by a grid. It is similar to other grid-based proxy graphs used to solve problems related to unit disk graphs, e.g.@ the construction of hop spanners [9]. For a current set $S \subset \mathbb {R}^2$ of sites, the vertices of *H* are those cells of the level-1 grid $\mathcal {G}_1$ (cf. Sect. 2.3) that contain at least one site of *S*, i.e., the cells $\sigma \in \mathcal {G}_1$ with $\sigma \cap S \ne \emptyset$. We call these cells *non-empty*, and we say that a site $s \in S$ is *assigned* to the cell $\sigma \in \mathcal {G}_1$ that contains it. We let $S(\sigma )$ denote the sites that are assigned to $\sigma$. Two distinct non-empty cells $\sigma$, $\tau$ are connected by an edge in *H* if and only if there is an edge $st\in \mathcal {D}(S)$ with $s \in S(\sigma )$ and $t \in S(\tau )$. The following lemma states that the proxy graph *H* is sparse and that it represents the connectivity in $\mathcal {D}(S)$:

### Lemma 3.1

The proxy graph *H* has at most *n* vertices, each with degree *O*(1). Two sites $s, t\in S$ are connected in $\mathcal {D}(S)$ if and only if their assigned cells $\sigma$ and $\tau$ are connected in *H*.

### Proof

Since every vertex of *H* has at least one site of *S* assigned to it, and since every site is assigned to exactly one vertex, there are at most *n* vertices in *H*. We say that two cells $\sigma$ and $\tau$ in $\mathcal {G}_1$ are *neighboring* if $\tau \in N_{5\times 5}(\sigma )$. Then, a cell $\sigma$ can be adjacent in *H* only to the *O*(1) neighboring cells of $\sigma$, as the distance to all other cells is larger than 2.

Next, we show that (i) for every edge *st* in $\mathcal {D}(S)$, the assigned cells $\sigma$ of *s* and $\tau$ of *t* are connected in *H*; and (ii) for every edge $\sigma \tau$ in *H*, all sites in $S(\sigma )$ can reach all sites in $S(\tau )$ in $\mathcal {D}(S)$. This immediately implies the claim about the connectivity, because we can then map paths in $\mathcal {D}(S)$ to paths in *H*, and vice versa. To prove (i), let *st* be an edge of $\mathcal {D}(S)$, and consider the assigned cells $\sigma , \tau$ with $s \in S(\sigma )$ and $t \in S(\tau )$. If $\sigma = \tau$, there is nothing to show. If $\sigma \ne \tau$, the definition of *H* immediately implies that the edge $\sigma \tau$ exists in *H*, and hence $\sigma$ and $\tau$ are connected. For (ii), we first note that for every vertex $\sigma$ in *H*, the sites in $S(\sigma )$ induce a clique in $\mathcal {D}(S)$. Indeed, for any $s, t \in S(\sigma )$, we have $\Vert st\Vert < |\sigma | = 2$, so the unit disks $D_s$ and $D_{t}$ intersect, and *st* is an edge in $\mathcal {D}(S)$. Now, let $\sigma \tau$ be an edge in *H*. By definition of *H*, there is at least one pair $s, t \in S$ with $s \in S(\sigma )$, and $t \in S(\tau )$ such that *st* is an edge of $\mathcal {D}(S)$. Then, as the sites in $S(\sigma )$ and the sites in $S(\tau )$ each form a clique in $\mathcal {D}(S)$, all sites in $S(\sigma )$ are connected to *s*, and all sites in $S(\tau )$ are connected to *t*, so every site in $S(\sigma )$ can reach every site in $S(\tau )$ by at most three steps in $\mathcal {D}(S)$. The claim follows. $\square$

In our connectivity structure, we maintain an HLT-structure $\mathcal {H}$ for *H*. To determine the connectivity between two sites *s* and *t*, we first identify the cells $\sigma$ and $\tau$ in $\mathcal {G}_1$ to which *s* and *t* are assigned. This requires *O*(1) time, because as mentioned in Sect. 2.5, we can store the assigned cell for *u* in its satellite data during the insertion of *u*. Then, we query $\mathcal {H}$ with the vertices $\sigma$ and $\tau$ of *H*, which, by Lemma 3.1, yields the correct answer. When a site *s* is inserted into or deleted from *S*, only the edges incident to the assigned cell $\sigma$ of *s* are affected. By Lemma 3.1, there are only *O*(1) such edges. Thus, once the set *E* of these edges is determined, by Theorem 2.2, we can update $\mathcal {H}$ in time $O(\log ^2 n)$.

We describe how to find the edges *E* of *H* that change when we update *S*. For every pair $\sigma ,\tau$ of neighboring cells in $\mathcal {G}_1$ where at least one cell is non-empty, we maintain a maximal bichromatic matching (MBM) $M_{\{\sigma ,\tau \}}$ for $R = S(\sigma )$ and $B = S(\tau )$, as in Lemma 2.7 (note that the special requirements of the lemma apply here). By definition, $\sigma \tau$ is an edge of *H* if and only if $M_{\{\sigma ,\tau \}}$ is not empty. When inserting or deleting a site *s* from *S*, we proceed as follows: let $\sigma \in \mathcal {G}_1$ be the cell assigned to *s*. We go through all cells $\tau \in N_{5\times 5}(\sigma )$, and we update $M_{\{\sigma ,\tau \}}$ by inserting or deleting *s* from the relevant set (creating or removing the underlying MBM structure if necessary). If the matching $M_{\{\sigma ,\tau \}}$ becomes non-empty during an insertion or empty during a deletion, we add the edge $\sigma \tau$ to *E* and mark it for insertion or remove $\sigma \tau$ from E and mark it for deletion, respectively. By Lemma 2.7, these updates to the MBM-structures take $O(\log ^2 n)$ time. Putting everything together, we obtain the main result of this section:

### Theorem 3.2

There is a dynamic connectivity structure for unit disk graphs such that the insertion or deletion of a site takes amortized time $O\left( \log ^2 n\right)$ and a connectivity query takes worst-case time $O(\log n/\log \log n)$, where *n* is the maximum number of sites at any time. The data structure requires *O*(*n*) space.

### Proof

The main part of the theorem follows from the discussion so far and Lemmas 3.1 and 2.7. For the space bound, note that the MBM s have size linear in the number of involved sites, and every site lies in a constant number of cells and MBM s. Also, the total number of edges in *H* is *O*(*n*), so the HLT-structure requires linear space as well. $\square$

**Remark.** Following the remark regarding Lemma 2.7, there is evidence that the update time of Theorem 3.2 can be improved. Using a better MBM data structure and a faster data structure for maintaining the proxy graph [24, 41] would lead to an improved update time (with possibly worse query and space bounds, depending on the data structures chosen).

## Polynomial Dependence on $\Psi$

We extend our structure from Theorem 3.2 to intersection graphs of arbitrary disks. Now, the running times will depend polynomially on the radius ratio $\Psi$. The general approach is unchanged, but the varying radii of the disks introduce new issues.

Again, we use a grid to structure the sites, but instead of the single grid $\mathcal {G}_1$, we rely on the first $\lfloor \log \Psi \rfloor + 1$ levels of the hierarchical grid $\mathcal {G}$ (see Sect. 2.3). Each site *s* is assigned to a grid level that is determined by the associated radius $r_s$. As in the other sections, we assume without loss of generality that all those cells are part of the same globally aligned grids. In Appendix A.1 we give the details on why this assumption can be made without negatively impacting the running time.

Since the disks have different sizes, we can no longer use Lemma 2.7 to maintain the maximal bichromatic matchings (MBM s) between neighboring grid cells. Instead, we use the more complex structure from Lemma 2.5. This increases the overhead for updating the MBM for each pair of neighboring cells.^6^ Furthermore, we will see that a disk can now intersect disks from $\Theta (\Psi ^2)$ other cells, generalizing the *O*(1)-bound from the unit disk case. Thus, the degree of the proxy graph, and hence the number of edges that need to be modified in a single update, depends on $\Psi$. To address this latter problem—at least partially—we describe in Sect. 4.2 how to refine the definition of the proxy graph so that fewer edges need to be modified in a single update. This will reduce the dependence on $\Psi$ in the update time from quadratic to linear. The query procedure becomes slightly more complicated, but the asymptotic running time remains unchanged.

We note that the approach in Sect. 4.1 is similar to the method of Kaplan et al. [27, Theorem 9.11] that achieves the same time and space bounds. However, our implementation uses a hierarchical grid instead of a single fine grid. This will be crucial for the improvement in Sect. 4.2, so we first describe the details of the modified approach.

### Extending the Unit Disk Case

As for unit disk graphs, we define a proxy graph *H* for $\mathcal {D}(S)$ that is based on grid cells and the intersections between the disks. The vertices of *H* are cells from the first $\lfloor \log \Psi \rfloor + 1$ levels of the hierarchical grid $\mathcal {G}$. More precisely, a site $s \in S$ is *assigned* to the cell $\sigma \in \mathcal {G}$ with $s \in \sigma$ and $|\sigma | \le r_s < 2 |\sigma |$.^7^ We define the *level* of *s* as the level of the cell $\sigma$ that *s* was assigned to. As before in Sect. 3, we denote the set of sites assigned to a cell $\sigma$ by $S(\sigma )$. It is still the case that all sites in $S(\sigma )$ form a clique in $\mathcal {D}(S)$, since for $s, t \in S(\sigma )$, we have $\Vert st\Vert \le |\sigma | \le r_s + r_t$. The vertices of *H* are all cells $\sigma$ of $\mathcal {G}$ with $S(\sigma ) \ne \emptyset$.

**Fig. 5:**
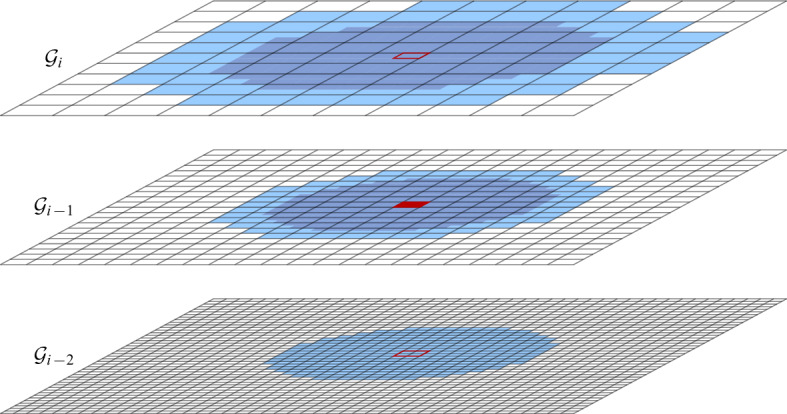
The neighboring cells of the red cell in $\mathcal {G}_{i-1}$. The area of the neighboring cells in one level beneath is colored in a darker shade

As in Sect. 3, we connect two cells $\sigma , \tau \in \mathcal {G}$ by an edge in *H* if and only if there are assigned sites $s \in S(\sigma )$ and $t \in S(\tau )$ such that *st* is an edge in $\mathcal {D}(S)$. Note that $\sigma$ and $\tau$ do not have to be on the same level. We call a pair of cells $\sigma$, $\tau$ *neighboring* if and only if it is possible that they could become adjacent in *H*, i.e., if some disks in $S(\sigma )$ and $S(\tau )$ could intersect. This is the case if the distance between $\sigma$ and $\tau$ is less than $2 | \sigma | + 2 | \tau |$, see Fig. 5. Since we are now dealing with cells from $\lfloor \log \Psi \rfloor + 1$ levels, the degree in *H* depends on $\Psi$.

#### Lemma 4.1

The proxy graph *H* has at most *n* vertices. Let $\sigma$ be a vertex of *H*, and let $\mathcal {N}(\sigma ) \subseteq \mathcal {G}$ be the neighboring cells of $\sigma$. Then, we have $|\mathcal {N}(\sigma )| = O(\Psi ^2)$, so *H* has maximum degree $O(\Psi ^2)$. Two sites of *S* are connected in $\mathcal {D}(S)$ if and only if their assigned cells are connected in *H*.

#### Proof

As in Lemma 3.1, the bound on the vertices follows from the facts that every vertex in *H* has at least one site assigned to it, and that every site is assigned to exactly one vertex. Now, let $\sigma$ be a vertex of *H*, and let $\mathcal {N}(\sigma )$ the set of neighboring cells for $\sigma$. Recall that all sites are assigned to the lowest $\lfloor \log \Psi \rfloor + 1$ levels of $\mathcal {G}$. Let $\ell \in \{0, \dots , \lfloor \log \Psi \rfloor \}$ be such that $\sigma \in \mathcal {G}_{\ell }$. First, fix an $\ell '$ with $\ell \le \ell ' \le \lfloor \log \Psi \rfloor$, and let $\sigma ' \supseteq \sigma$ be the cell at level $\ell '$ that contains $\sigma$. As can be seen by simple volume considerations, all cells in $\mathcal {N}(\sigma ) \cap \mathcal {G}_{\ell '}$ lie in the neighborhood $N_{13\times 13}(\sigma ')$, so there are at most $13^2$ of them. Next, consider $\ell '$ with $0 \le \ell ' < \ell$. All cells of $\mathcal {N}(\sigma ) \cap \mathcal {G}_{\ell '}$ are contained in the region $N_{13 \times 13}(\sigma )$. Thus, since every cell of $\mathcal {G}_\ell$ contains exactly $4^{\ell - \ell '}$ cells of $\mathcal {G}_{\ell '}$, we have $|\mathcal {N}(\sigma ) \cap \mathcal {G}_{\ell '}| \le 13^2 \cdot 4^{\ell - \ell '}$. Altogether, $|\mathcal {N}(\sigma )|$ is at most

$$\begin{aligned} 13^2 \cdot \sum _{i=0}^{\lfloor \log \Psi \rfloor } 4^i = O(4^{\log \Psi }) = O(\Psi ^2). \end{aligned}$$

Now, the claim on the maximum degree of *H* is immediate. The claim on the connectivity is shown verbatim as in the proof of Lemma 3.1. $\square$

We now describe the details of our data structure. The main ingredient is a *quadforest* $\mathcal {F}$. The quadforest helps us find the relevant edges of the proxy graph *H* that need to be updated. Let $\mathcal {C} = \bigcup _{\sigma \in H} \{\sigma \} \cup \mathcal {N}(\sigma )$ be the set of all vertices $\sigma$ in *H* together with their neighboring cells in $\mathcal {G}$.

Let $\mathcal {C}'$ be the set of all cells in $\mathcal {G}_{\lfloor \log \Psi \rfloor }$ that contain at least one cell from $\mathcal {C}$. For each cell $\rho \in \mathcal {C}'$, we construct a *quadtree* $\mathcal {T}_{\rho }$ (cf. Sect. 2.3): we make $\rho$ the root of $\mathcal {T}_{\rho }$. If $\rho$ properly contains a cell of $\mathcal {C}$, we take the four cells of $\mathcal {G}$ that lie in $\rho$ in the level below, and we add each such subcell as a child to $\rho$ in $\mathcal {T}_{\rho }$. We recurse on all children of $\rho$ that still properly contain a cell from $\mathcal {C}$, until $\mathcal {T}_{\rho }$ is complete. The resulting set of trees $\mathcal {T}_\rho$, for all $\rho \in \mathcal {C}'$, constitutes our quadforest $\mathcal {F}$. Since $\mathcal {C}$ contains the full neighborhood for every vertex in *H*, the quadforest $\mathcal {F}$ has $O(\Psi ^2 n)$ nodes, by Lemma 4.1. The number of quadtrees in $\mathcal {F}$ is *O*(*n*), since the roots for the neighboring cells of a vertex $\sigma \in H$ must be in the $(13 \times 13)$-neighborhood of the root for $\sigma$. We store the *O*(*n*) quadtree root cells of $\mathcal {F}$ in a red-black tree [15] that allows us to locate a root in $O(\log n)$ time when given the coordinates of its lower-left corner. We have an MBM-structure of Lemma 2.5 for every pair of neighboring cells $\sigma$, $\tau$ such that $S(\sigma ) \ne \emptyset$ or $S(\tau ) \ne \emptyset$, containing the sites from $S(\sigma )$ and $S(\tau )$. The proxy graph *H* is represented by an HLT-structure $\mathcal {H}$ (cf. Theorem 2.2). In $\mathcal {H}$, we store a vertex for every cell $\sigma$ with $S(\sigma ) \ne \emptyset$ and edge $\sigma \tau$ for every pair of neighboring cells $\sigma$ and $\tau$ whose MBM is nonempty.

**Fig. 6:**
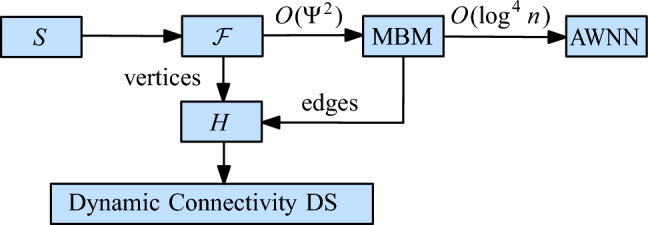
The structure of our initial data structure for general disks

For every cell $\sigma$ in $\mathcal {F}$, we store the set $S(\sigma )$ of assigned sites (possibly empty) as well as a red-black tree containing all MBM-structures that involve $\sigma$. This red-black tree uses as index the center of the other cell $\tau$ that defines the MBM-structure together with $\sigma$. The center of a cell is unique, even across different cell sizes. The total space for $\mathcal {F}$, the red-black tree of the roots, and $\mathcal {H}$ is $O(\Psi ^2 n)$, so the space is dominated by the total size of the MBM -structures. By Lemma 2.5, this is bounded by $O(\Psi ^2 n \log n)$, since every site appears in at most $O(\Psi ^2)$ MBM s. See Fig. 6 for an overview of the structure.

To perform a connectivity query for two sites *s* and *t*, we first obtain the cells $\sigma$ and $\tau$ with $s \in S(\sigma )$ and $t \in S(\tau )$. This takes *O*(1) time, because this information can be stored with a site when it is inserted into the structure. Then, we query the HLT-structure $\mathcal {H}$ with $\sigma$ and $\tau$, in $O(\log n / \log \log n)$ time. By Lemma 4.1, this yields the correct result.

To insert a site *s* into *S*, we determine the cell $\sigma \in \mathcal {G}$ to which *s* is assigned, as well as the set of neighboring cells $\mathcal {N}(\sigma )$ of $\sigma$. We locate all cells of $\{ \sigma \} \cup \mathcal {N}(\sigma )$ in $\mathcal {F}$, creating new quadtree nodes if necessary. This takes $O(\Psi ^2 + \log n)$ time, by Lemma 4.1 and the time required to obtain the quadtree roots. We add *s* to $S(\sigma )$, and we insert *s* into the MBM structures for $\sigma$ and every neighboring cell $\tau \in \mathcal {N}(\sigma )$, creating new MBM-structures if necessary. For every MBM that becomes non-empty we insert an edge into the HLT-structure $\mathcal {H}$. Since $|\mathcal {N}(\sigma )| = O(\Psi ^2)$, by Lemmas 2.5 and 4.1, updating the MBM s needs amortized expected time $O(\Psi ^2 \log ^{4} n)$ (this also includes the time for inserting the new MBM s into their respective red-black trees), and the update in $\mathcal {H}$ needs $O(\Psi ^2 \log ^2 n)$ amortized time. Thus, the total amortized expected time for an insertion is $O(\Psi ^2 \log ^{4} n)$.

Similarly, to delete a site $s \in S$, we locate the cell $\sigma$ with $s \in S(\sigma )$ together with the set of neighboring cells $\mathcal {N}(\sigma )$ of $\sigma$ in $\mathcal {F}$. This takes $O(\Psi ^2 + \log n)$ time, by Lemma 4.1 and the time required to obtain the quadtree roots. Then, we delete *s* from $S(\sigma )$ and from all MBM s for $\sigma$ and a neighboring cell $\tau$. If now $S(\sigma ) = \emptyset$, we also delete for all neighboring cells $\tau$ with $S(\tau ) = \emptyset$ the MBM for $\sigma$ and $\tau$. Since $\sigma$ has $O(\Psi ^2)$ neighboring cells, all this needs amortized expected time $O(\Psi ^2 \log ^{4} n)$ (including the time for deleting the MBM s from their respective red-black trees). Afterwards, for all $\tau \in \mathcal {N}(\sigma )$ whose MBM with $\sigma$ has become empty or was deleted through the deletion of *s*, we delete the edge $\sigma \tau$ from the HLT-structure $\mathcal {H}$. Since there are $O(\Psi ^2)$ such edges, this takes $O(\Psi ^2 \log ^2 n)$ amortized time. Finally, we delete from $\mathcal {F}$ all cells $\tau \in \{ \sigma \} \cup \mathcal {N}(\sigma )$ that have $S(\tau ) = \emptyset$ and that no longer occur in the neighborhood of any vertex of *H* with an assigned site. The last condition corresponds to all cells of $\{ \sigma \} \cup \mathcal {N}(\sigma )$ with an empty MBM red-black tree. Hence, they can be obtained in $O(\Psi ^2 + \log n)$ time. Overall, the deletion time is dominated by the updates in the MBM s, and it is bounded by $O(\Psi ^2 \log ^{4} n)$. We obtain the following theorem:

#### Theorem 4.2

There is a fully dynamic connectivity structure for disk graphs of bounded radius ratio $\Psi$ such that an update takes amortized expected time $O(\Psi ^2 \log ^{4} n)$ and a connectivity query takes worst-case time $O(\log n/\log \log n)$, where *n* is the maximum number of sites at any time. The data structure requires $O(\Psi ^2 n \log n)$ space.

### Improving the Dependence on $\Psi$

To improve over Theorem 4.2, we show how to reduce the degree of the proxy graph *H* from $O(\Psi ^2)$ to $O(\Psi )$. The intuition is that it suffices to focus on disks that are not contained in any other disk of *S* to maintain the connected components of $\mathcal {D}(S)$. We call these disks *(inclusion) maximal*. Then, we need to consider only edges between disks that intersect *properly*, i.e., their boundaries intersect. If we want to perform a connectivity query between two sites *s* and *t*, we must find appropriate maximal disks in *S* that contain $D_s$ and $D_t$. See Fig. 9 for examples depicting this intuition.

The following definition formalizes the notion of containment between disks. The definition is based on grids, since we must take into account not only the disks that are currently present in the structure, but also the disks that could be inserted in the future.

#### Definition 4.3

Let *D* be a disk and $\sigma \in \mathcal {G}$ a grid cell with level at most $\lfloor \log \Psi \rfloor$. We say that $\sigma$ is *Minkowski covered* by *D* if and only if every possible disk that can be assigned to $\sigma$ fully lies in *D*. We call $\sigma$ *maximal* if and only if there is no larger cell $\tau \supset \sigma$ in $\mathcal {G}$ that is Minkowski covered by *D*.

**Fig. 7:**
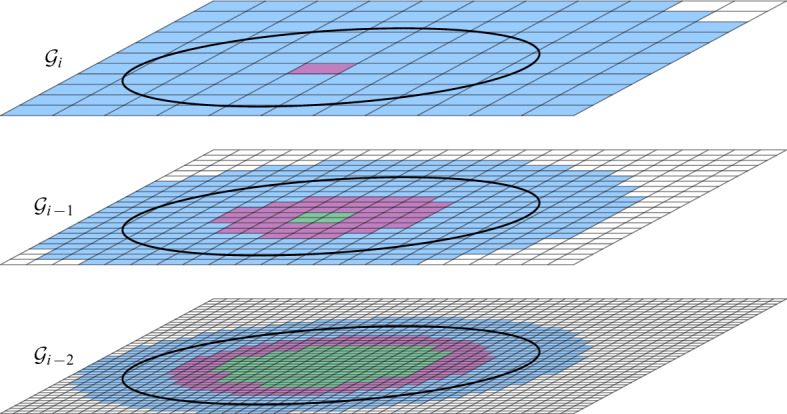
The types of cells that require checking when updating the black disk in the data structure of Theorem 4.2:
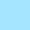
$\mathcal {N}_1$: not Minkowski covered
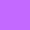
$\mathcal {N}_2$: maximal Minkowski covered
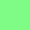
$\mathcal {N}_3$: Minkowski covered, not maximal

Equivalently, Definition 4.3 states that $\sigma$ is Minkowski covered by *D* if and only if (i) $\sigma$ has level at most $\lfloor \log \Psi \rfloor$ and (ii) the Minkowski sum of $\sigma$ with an open disk of radius $2|\sigma |$ is contained in *D*. By definition, if $\sigma$ is Minkowski covered by *D*, then all smaller cells $\tau \in \mathcal {G}$ with $\tau \subset \sigma$ are also Minkowski covered by *D*. The following lemma bounds the number of cells of different types, for a given disk. See Fig. 7 for an illustration.

#### Lemma 4.4

Let $s \in S$ be a site, and let $\mathcal {N}(s)$ be the set of cells of $\mathcal {G}$ that may have an assigned disk that intersects $D_s$. Write $\mathcal {N}(s)$ as the disjoint union $\mathcal {N}(s) = \mathcal {N}_1(s) \mathrel {{\dot{\cup }}} \mathcal {N}_2(s) \mathrel {{\dot{\cup }}} \mathcal {N}_3(s)$, such that (i) $\mathcal {N}_1(s)$ consists of the cells that are not Minkowski covered by $D_s$, (ii) $\mathcal {N}_2(s)$ consists of the cells that are maximal Minkowski covered by $D_s$, and (iii) $\mathcal {N}_3(s)$ consists of the cells that are Minkowski covered by $D_s$, but not maximal with this property. Then, we have $|\mathcal {N}_1(s) \cup \mathcal {N}_2(s)| = O(\Psi )$ and $|\mathcal {N}_3(s)| = O(\Psi ^2)$.

#### Proof

First, we show that $|\mathcal {N}_1(s)| = O(\Psi )$. Let $\ell \in \{0, \dots , \lfloor \log \Psi \rfloor \}$ be the level of *s*, and let $\sigma \in \mathcal {G}_\ell$ be the cell with $s \in S(\sigma )$. By definition, all cells of $\mathcal {N}_1(s)$ have level at most $\lfloor \log \Psi \rfloor$. We bound for each level $\ell ' \in \{0, \dots , \lfloor \log \Psi \rfloor \}$ the size of $\mathcal {N}_1(s) \cap \mathcal {G}_{\ell '}$. For $\ell ' \ge \ell$, there are *O*(1) cells in $\mathcal {G}_{\ell '}$ that are neighboring to $\sigma$, so we have $|\mathcal {N}_1(s) \cap \mathcal {G}_{\ell '}| = O(1)$. For $\ell ' < \ell$, we observe that any cell in $\mathcal {N}_1(s) \cap \mathcal {G}_{\ell '}$ must intersect the annulus $A_s$ that is centered at the boundary of $D_s$ and has width $\Theta (2^{\ell '})$, see Fig. 7. The area of $A_s$ is $\Theta (r_s \cdot 2^{\ell '}) = \Theta (2^{\ell + \ell '})$. Since the cells at level $\ell '$ have pairwise disjoint interiors and area $\Theta (2^{2\ell '})$, a simple volume argument shows that $|\mathcal {N}_1(s) \cap \mathcal {G}_{\ell '}| = O(2^{\ell - \ell '})$. Adding over all $\ell '$, we get

$$\begin{aligned} |\mathcal {N}_1(s)| = \sum _{\ell ' = 0}^{\lfloor \log \Psi \rfloor } |\mathcal {N}_1(s) \cap G_{\ell '}|&= \sum _{\ell ' = 0}^{\ell - 1} |\mathcal {N}_1(s) \cap G_{\ell '}| + \sum _{\ell ' = \ell }^{\lfloor \log \Psi \rfloor }|\mathcal {N}_1(s) \cap G_{\ell '}|\\&= \sum _{\ell ' = 0}^{\ell - 1} O\Big (2^{\ell - \ell '}\Big ) + \sum _{\ell ' = \ell }^{\lfloor \log \Psi \rfloor }O(1)\\&= O(2^\ell ) + O(\log \Psi ) = O(\Psi ). \end{aligned}$$

Next, we bound $|\mathcal {N}_1(s) \cup \mathcal {N}_2(s)|$. For this, we note that every cell in $\mathcal {N}_2(s)$ either (i) has level $\lfloor \log \Psi \rfloor$ (and there are *O*(1) such cells that are Minkowski covered by $D_s$), or (ii) is one of the four cells that are directly contained in a cell $\sigma \in \mathcal {N}_1(s)$ and have diameter $|\sigma |/2$. Hence, we get $|\mathcal {N}_1(s) \cup \mathcal {N}_2(s)| = O(|\mathcal {N}_1(s)|) = O(\Psi )$. The bound on $|\mathcal {N}_3(s)|$ follows from Lemma 4.1. $\square$

Now, we show how to reduce the degree of the proxy graph. Let *H* be the proxy graph from Sect. 4.1, and let $H'$ be a subgraph of *H* that is defined as follows: as in *H*, the vertices of $H'$ are all the grid cells $\sigma \in \mathcal {G}$ with $S(\sigma ) \ne \emptyset$. Let $\sigma , \tau$ be two distinct vertices of $H'$. Then, there is an edge between $\sigma$ and $\tau$ in $H'$ if and only if there are sites $s \in S(\sigma )$ and $t \in S(\tau )$ such that (i) $D_s$ and $D_t$ intersect; (ii) $D_s$ does *not* Minkowski cover $\tau$; and (iii) $D_t$ does *not* Minkowski cover $\sigma$. From the definition, it is immediate that $H'$ is a subgraph of *H*, and that there is an edge between $\sigma$ and $\tau$ if and only there are sites $s \in S(\sigma )$ and $t \in S(\tau )$ such that (i) $D_s$ and $D_t$ intersect; (ii) $\tau \in \mathcal {N}_1(s)$; and (iii) $\sigma \in \mathcal {N}_1(t)$.

**Fig. 8:**
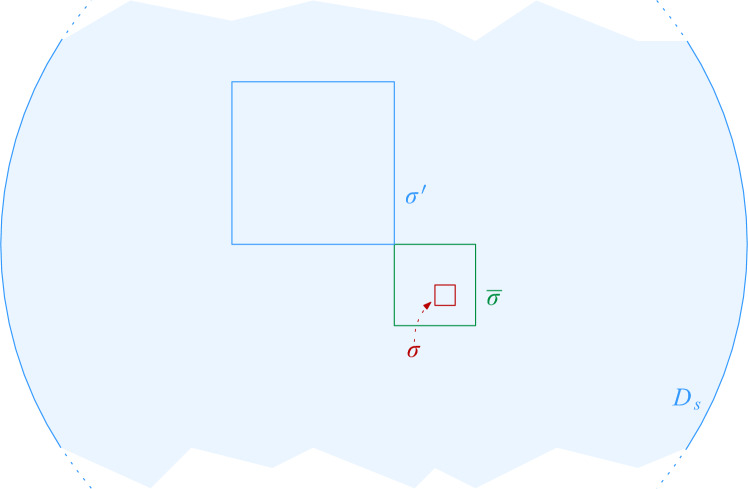
Obtaining the proxy vertex for $\sigma$. We assume $\overline{\sigma }$ is the largest cell which (i) contains $\sigma$ and (ii) is Minkowski covered by a disk. If $D_s$ is the largest disk doing so, and we have $s \in S(\sigma ')$, then $\sigma '$ is the proxy vertex for $\sigma$. This means in particular that for each $v \in S(\sigma )$ the disk $D_s$ properly contains $D_v$

Let $\sigma$ be a vertex in $H'$. In order to implement connectivity queries in the sparsified graph $H'$, we define the *proxy vertex* $\sigma '$ of $\sigma$ in $H'$ as follows: if there is no site $s \in S$ such that $D_s$ Minkowski covers $\sigma$, then $\sigma ' = \sigma$. Otherwise, let $\overline{\sigma }$ be the maximal cell such that (i) $\overline{\sigma }$ contains $\sigma$ and (ii) there is a site $s \in S$ such that $\overline{\sigma }$ is Minkowski covered by $D_s$. Let $t \in S$ be a site of maximum radius such that $D_t$ Minkowski covers $\overline{\sigma }$, and let $\rho$ be the cell that *t* is assigned to, i.e., $\rho$ is the cell with $t \in S(\rho )$. Then, the proxy vertex $\sigma '$ for $\sigma$ in $H'$ is defined as $\rho$. If there are multiple choices for *t*, we fix an arbitrary one. See Fig. 8 for an illustration. The next lemma shows that $H'$ preserves the connectivity between proxy vertices.

#### Lemma 4.5

Let $s, t \in S$ be two sites, and let $\sigma , \tau$ be the cells with $s \in S(\sigma )$ and $t \in S(\tau )$. Let $\sigma '$ and $\tau '$ be the proxy vertices for $\sigma$ and $\tau$ in $H'$. Then, $\sigma '$ and $\tau '$ are connected in $H'$ if and only if *s* and *t* are connected in $\mathcal {D}(S)$.

#### Proof

First, suppose that $\sigma '$ and $\tau '$ are connected in $H'$. The cells $\sigma '$ and $\tau '$ are connected in *H*, since $H'$ is a subgraph of *H*. From the definition of proxy vertices it is immediate that $\sigma '$ is adjacent in *H* to $\sigma$ and that $\tau '$ is adjacent in *H* to $\tau$. Thus, $\sigma$ and $\tau$ are connected in *H*, and Lemma 4.1 shows that *s* and *t* are connected in $\mathcal {D}(S)$.

Next, suppose that *s* and *t* are connected in $\mathcal {D}(S)$. We call a disk $D_u$, $u \in S$, *(inclusion) maximal* if there is no site $v \in S$ with $u \ne v$ and $D_u \subset D_v$. Our strategy is to consider a path $\pi$ of *(inclusion) maximal* disks that connects *s* and *t* in $\mathcal {D}(S)$, and to show that $\pi$ induces a path $\pi '$ between $\sigma '$ and $\tau '$ in $H'$. To construct $\pi$, we first find a maximal disk $D_{s'}$ that *represents* $D_s$, as follows: if the proxy vertex $\sigma '$ is different from $\sigma$, we let $D_{s'}$ be the disk used to define $\sigma '$, i.e., the disk of maximum radius that Minkowski covers $\overline{\sigma }$, the maximal Minkowski covered cell that contains $\sigma$. Then, the disk $D_{s'}$ is indeed maximal, since any disk that properly contains $D_{s'}$ would have larger radius and would Minkowski cover $\overline{\sigma }$, contradicting the choice of $D_{s'}$. If the proxy vertex $\sigma '$ is $\sigma$ itself, we let $D_{s'}$, $s' \in S$, be an arbitrary maximal disk that contains $D_s$. Possibly, this may be $D_s$ itself, but it also may be that $s' \not \in S(\sigma )$. In the same way, we obtain a maximal disk $D_{t'}$ that represents $D_t$.

**Fig. 9:**
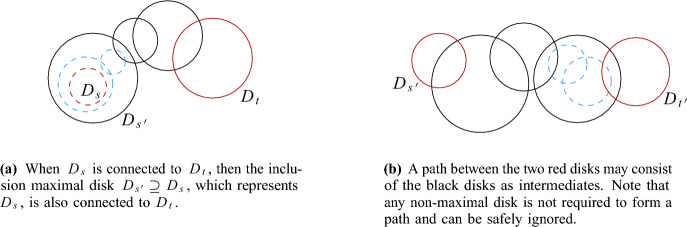
The two main ingredients for the construction of path $\pi$ in Lemma 4.5

Now, we claim that there is a path $\pi$ in $\mathcal {D}(S)$ between $s'$ and $t'$ that uses only maximal disks: by our choice of $s'$ and $t'$, we know that $s'$ is in the same connected component as *s*, and $t'$ is in the same connected component as *t*. Since we assumed that *s* and *t* are connected in $\mathcal {D}(S)$, this also holds for $s'$ and $t'$. Consider a path in $\mathcal {D}(S)$ between $s'$ and $t'$, and replace every disk along this path by a maximal disk that contains it. The resulting path $\pi$ has the required property (possibly after making shortcuts between duplicate disks). See Fig. 9 for an illustration.

**Fig. 10:**
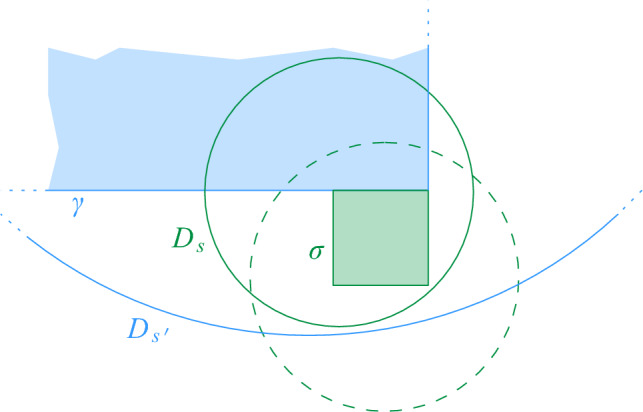
In the proof of Lemma 4.5 the first cell of $\pi '$ might not be the proxy vertex of $\sigma$. This occurs if $\sigma$ is its own proxy cell, there exist a maximal disk $D_{s'}$ containing $D_s$, $s' \not \in S(\sigma )$, and $D_{s'}$ does not Minkowski cover $\sigma$. In this example, we have $s \in S(\sigma )$, $s' \in S(\gamma )$, and the dashed disk would be assigned to $\sigma$, hence $D_{s'}$ does not Minkowski cover $\sigma$

Consider the sequence $\pi '$ of cells in $H'$ that we obtain by replacing every site *u* in $\pi$ by the vertex $\sigma _u$ of $H'$ with $u \in S(\sigma _u)$, and by taking shortcuts between duplicate cells. Then, $\pi '$ is a path in $H'$, since the definition of $H'$ implies that the assigned cells for two intersecting maximal disks of *S* must be either identical or adjacent in $H'$. Furthermore, the first cell of $\pi '$ is either the proxy vertex $\sigma '$ or adjacent in $H'$ to $\sigma '$. The latter may happen if $\sigma ' = \sigma$ and *S* has a disk that contains $D_s$, but no disk that Minkowski covers $\sigma$, see Fig. 10. Similarly, the last cell of $\pi '$ is either $\tau '$ or a cell that is adjacent to $\tau '$ in $H'$. Thus, by possibly adding $\sigma '$ and/or $\tau '$ to $\pi '$, we obtain a path $\pi '$ in $H'$ that connects $\sigma '$ and $\tau '$. $\square$

Lemma 4.5 implies that it is enough to maintain the sparsified proxy graph $H'$ in an HLT-structure $\mathcal {H}$. To perform updates and queries efficiently, we need to do two things: first, we need to determine which edges in $H'$ are affected by an update in *S*; second, in order to perform a connectivity query between two sites *s* and *t*, we not only need to determine the assigned cells $\sigma$ and $\tau$ for *s* and *t*, but we must also find the proxy vertices $\sigma '$ for $\sigma$ and $\tau '$ for $\tau$ in $H'$. We again maintain a *quadforest* $\mathcal {F}$: for each $s \in S$, let $\sigma _s$ be the cell with $s \in S(\sigma _s)$, and let $\mathcal {C} = \bigcup _{s \in S} \{\sigma _s\} \cup \mathcal {N}_1(s) \cup \mathcal {N}_2(s)$, with the notation from Lemma 4.4.^8^

As in Sect. 4.1, we use $\mathcal {C}$ to define $\mathcal {F}$. Let $\mathcal {C}'$ be the set of all cells in $\mathcal {G}_{\lfloor \log \Psi \rfloor }$ that contain at least one cell from $\mathcal {C}$. For each $\rho \in \mathcal {C}'$, let $\mathcal {T}_\rho$ be the quadtree for the cells in $\mathcal {C}$ that lie in $\rho$ (cf. Sect. 4.1). Let $\mathcal {F}$ be the quadforest that contains all quadtrees $\mathcal {T}_\rho$, $\rho \in \mathcal {C}'$. By Lemma 4.4, the quadforest $\mathcal {F}$ contains $O(\Psi n)$ nodes, and as in Sect. 4.1, we see that is has *O*(*n*) roots. We store the root cells of $\mathcal {F}$ in a red-black tree [15], indexed by the lower-left corner of the cells. We have an MBM-structure for every pair of cells $\sigma$, $\tau$ with $S(\sigma ) \ne \emptyset$ and $\tau \in \bigcup _{ s \in S(\sigma ) } \mathcal {N}_1(s)$ or with $S(\tau ) \ne \emptyset$ and $\sigma \in \bigcup _{t \in S(\tau )} \mathcal {N}_1(t)$. The MBM-structure for $\sigma$ and $\tau$ contains all sites $s \in S(\sigma )$ with $\tau \in \mathcal {N}_1(s)$ and all sites $t \in S(\tau )$ with $\sigma \in \mathcal {N}_1(t)$. The proxy graph *H* is represented by an HLT-structure $\mathcal {H}$ (cf. Theorem 2.2). In $\mathcal {H}$, we store a vertex for every cell $\sigma$ with $S(\sigma ) \ne \emptyset$ and edge $\sigma \tau$ for every pair of cells $\sigma$ and $\tau$ whose MBM is nonempty.

For every cell $\sigma$ in $\mathcal {F}$, we store several data structures. First, we maintain the set $S(\sigma )$ of assigned sites for $\sigma$ (possibly empty). Second, we store the set $\mathcal {C}(\sigma )$ of all sites $s \in S$ such that $\sigma$ is maximal Minkowski covered by $D_s$, i.e., such that $\sigma \in \mathcal {N}_2(s)$. The set $\mathcal {C}(\sigma )$ is represented as a red-black tree, where the ordering is determined by the associated radius $r_s$. In addition, a pointer to the maximum element is maintained, allowing its retrieval in *O*(1) time. Third, we have the MBM s that involve $\sigma$. The MBM s are organized in a red-black tree, indexed by the lower-left corner of a neighboring vertex $\tau$. The total space for $\mathcal {F}$, the red-black tree of roots, the $\mathcal {C}(\sigma )$ structures and $\mathcal {H}$ is $O(\Psi n)$, so the space is dominated by the total size of the MBM-structures. This is bounded by $O(\Psi n \log n)$, since every site appears in at most $O(\Psi )$ MBM s.

**Fig. 11:**
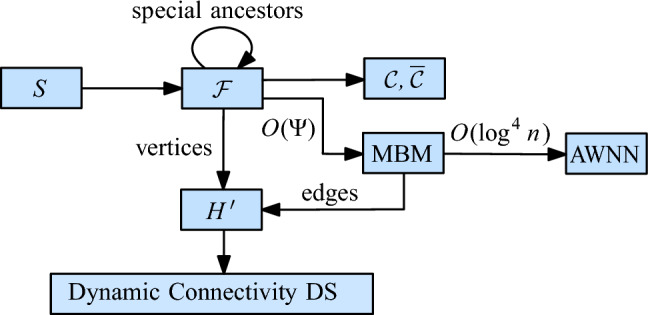
The structure of our data structure for general disks

Let $q = \lceil \log \log \Psi / 2 \rceil$. In the quadforest $\mathcal {F}$, we designate the cells with level $i \cdot q$, for $0 \le i \le \lfloor \log \Psi \rfloor /q$ as *special*. In $\mathcal {F}$, we maintain a pointer from every special cell $\rho$ to the lowest special ancestor of $\rho$, lying *q* levels above. Additionally, the special cell $\rho$ stores a red-black tree $\overline{\mathcal {C}}(\rho )$ that contains the elements of the red-black trees $\mathcal {C}(\rho ')$ for all ancestors $\rho '$ of $\rho$ that are between $\rho$ and the next special level above it. Since the elements from a red-black tree $\mathcal {C}(\rho ')$ appear in at most $4^q = O(\log \Psi )$ special red-black trees, the total additional space is $O( \Psi n \log \Psi )$. It follows that the whole structure needs $O(\Psi n (\log n + \log \Psi ))$ space. See Fig. 11 for an overview of the data structure.

Using $\mathcal {F}$ we can perform a connectivity query for two sites *s* and *t*. Let $\sigma$ and $\tau$ be the cells with $s \in S(\sigma )$ and $t \in S(\tau )$. As in Sect. 4.1, we can store these cells with the sites *s* and *t* and retrieve them in *O*(1) time. We describe how to find the proxy vertex $\sigma '$ for $\sigma$, the procedure for $\tau$ is analogous: first, we obtain the maximal cell $\overline{\sigma } \supseteq \sigma$ that contains $\sigma$ and is Minkowski covered by a disk from *S* (if it exists). For this, we ascend from $\sigma$ in its respective quadtree for at most *q* steps, until we reach the closest special level above $\sigma$. From there, we follow the pointers that connect the special levels, until the root. For each cell $\rho$ along the way, we check if the red-black tree $\mathcal {C}(\rho )$ (for a non-special cell) or the red-black tree $\overline{\mathcal {C}}(\rho )$ (for a special cell) is non-empty, in *O*(1) time. Let $\rho '$ be the largest cell with this property. If $\rho '$ is non-special, we set $\overline{\sigma } = \rho '$. If $\rho$ is special, we ascend from $\rho '$ towards the root until the next special level, to find the largest cell $\overline{\sigma }$ along the way that has $\mathcal {C}(\overline{\sigma })$ non-empty. At the end, we have found the largest ancestor $\overline{\sigma }$ of $\sigma$ that is Minkowski covered by a disk $D_s$, $s \in S$, if it exists. Now, if $\overline{\sigma }$ does not exist, we set $\sigma ' = \sigma$. Otherwise, let *u* be the site of maximum radius that is stored in the red-black tree $\mathcal {C}(\overline{\sigma })$ and set $\sigma ' = \sigma _u$, where $\sigma _u$ is the cell with $u \in S(\sigma _u)$. Once $\overline{\sigma }$ is known, it takes *O*(1) time to find $\sigma '$. Thus, the total time to find $\sigma '$ (and $\tau '$) is $O(\log \Psi / \log \log \Psi )$. Once $\sigma '$ and $\tau '$ are determined, we use them to query the connectivity structure $\mathcal {H}$, in $O(\log n/\log \log n)$ time, and we return the result. By Lemma 4.5, this gives the correct answer. The overall running time is $O(\log n/\log \log n + \log \Psi /\log \log \Psi )$.

To insert a site *s* into *S*, we determine the cell $\sigma \in \mathcal {G}$ to which *s* is assigned, as well as the sets $\mathcal {N}_1(s)$ and $\mathcal {N}_2(s)$ of neighboring cells. We locate all cells of $\{\sigma \} \cup \mathcal {N}_1(s) \cup \mathcal {N}_2(s)$ in $\mathcal {F}$, creating new quadtree nodes if necessary. This takes $O(\Psi + \log n)$ time, by Lemma 4.4 and the time to obtain the quadtree roots. We add *s* to $S(\sigma )$, and we insert *s* into the MBM-structures for $\sigma$ and every neighboring cell $\tau \in \mathcal {N}_1(s)$, creating new MBM-structures if necessary. Finally, for $\tau \in \mathcal {N}_2(s)$, we insert *s* into the red-black tree $\mathcal {C}(\tau )$ and into the special red-black trees $\overline{\mathcal {C}}(\tau ')$ for all special descendants of $\tau$ that are at most *q* levels below it, creating cells as necessary. For every MBM that becomes non-empty, we insert a corresponding edge into the HLT-structure $\mathcal {H}$. Since $|\mathcal {N}_1(s) \cup \mathcal {N}_2(s)| = O(\Psi )$, by Lemma 4.4, updating the MBM s takes $O(\Psi \log ^4 n)$ amortized expected time, while updating the red-black trees $\mathcal {C}(\tau )$ and the edges in $\mathcal {H}$ takes $O(\Psi \log ^2 n)$ amortized time. The special red-black trees can be updated in $O(\Psi \log \Psi \log n)$ time, since *s* needs to be inserted into $O(\Psi \log \Psi )$ of them. The overall running time is $O(\Psi (\log ^4 n + \log n\log \Psi ))$.

To delete a site *s* from *S*, we locate in $\mathcal {F}$ the cell $\sigma$ with $s \in S(\sigma )$ as well as the sets $\mathcal {N}_1(s)$ and $\mathcal {N}_2(s)$ of neighboring cells. This takes $O(\Psi + \log n)$ time, by Lemma 4.4. We remove *s* from $S(\sigma )$ and from the MBM-structures for $\sigma$ and every cell $\tau \in \mathcal {N}_1(s)$. For $\tau \in \mathcal {N}_2(s)$, we delete *s* from the red-black tree $\mathcal {C}(\tau )$ as well as the special red-black trees $\overline{\mathcal {C}}(\tau ')$ for all special descendants of $\tau$ that are at most *q* levels below it. For every MBM that becomes empty, we delete the corresponding edge from the HLT-structure $\mathcal {H}$. Finally, we delete from $\mathcal {F}$ all cells $\tau$ that have $S(\tau ) = \emptyset$, for which no site *t* with $\tau \in \mathcal {N}_1(t) \cup \mathcal {N}_2(t)$ exists, and which also don’t have a special cell $\tau '$ with non-empty red-black tree $\overline{\mathcal {C}}(\tau ')$ as descendant. Since $|\mathcal {N}_1(s) \cup \mathcal {N}_2(s)| = O(\Psi )$, by Lemma 4.4, updating the MBM s takes $O(\Psi \log ^4 n)$ amortized expected time, while updating the red-black trees $\mathcal {C}(\tau )$ and the edges in $\mathcal {H}$ takes $O(\Psi \log n)$ amortized time. The special red-black trees can be updated in $O(\Psi \log \Psi \log n)$ time, since $O(\Psi \log \Psi )$ of them contain *s*. The overall running time is $O(\Psi (\log ^4 n + \log n\log \Psi ))$.

#### Theorem 4.6

There is a data structure for dynamic disk connectivity with amortized expected update time $O(\Psi \log ^{4} n)$ and query time $O(\log n/\log \log n)$. It needs $O(\Psi n \log n)$ space.

#### Proof

This is immediate by the discussion above and the fact that we can assume $\Psi = O(n^3)$, since otherwise the theorem follows from the trivial algorithm that completely recomputes all connected components after every update. $\square$

## Disk Reveal Data Structure

**Fig. 12:**
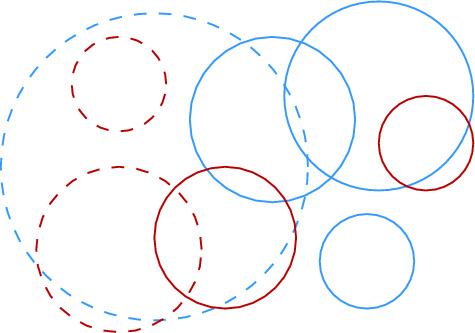
When removing a blue disk *b*, we want to obtain all red disks that intersect *b* but no other blue disk. For example, after removing the dashed blue disk, the dashed red disks are revealed and need to be reported

In Sect. 6, we will construct data structures for the semi-dynamic setting. Before that, we address the following problem: we are given two dynamic sets *R* and *B* of disks, such that the disks in *R* can be both inserted and deleted, while the disks in *B* can only be deleted. We would like to maintain *R* and *B* in a data structure such that whenever we delete a disk *b* from *B*, we receive a list of all the disks in the current set *R* that intersect the disk *b* but no other disk from the remaining set $B \setminus \{b\}$. We call such disks *revealed* by the deletion of *b*; see Fig. 12. Using this *disk reveal* data structure, we will be able to obtain efficiently the affected edges during an update in the decremental setting, as described in Sect. 6.

We construct the disk reveal structure in Sects. 5.2 and 5.3, and state our final result in Theorem 5.7. The central idea is to represent the intersections between each disk $r \in R$ and the disks in *B* sparsely by assigning *r* to one disk $b \in B$ that intersects it. If *b* gets deleted, we either report *r* as revealed, or we determine a new disk of *B* that intersects *r*. To ensure that the assignments are not updated too often (at least in expectation), we choose the assigned disk of *r* at random and assume an oblivious adversary. Thus, given *r*, we must be able to randomly sample a disk among all disks in *B* that intersect it.

Our main task in this section is to obtain a dynamic data structure for disks that allows for *random sample queries*: given a query disk *D*, report a random disk among all disks in the current set that intersect *D*. We build upon the dynamic lower envelope data structure by Kaplan et al. [27], which we briefly review in Sect. 5.1. We proceed in two steps: first, we describe a simpler data structure for sampling a random disk that contains a given query *point*. This builds on the data structure of Kaplan et al.@ for planes [27, Sect. 7]. Afterwards, we extend the result to the data structure of continuous bivariate functions of constant description complexity [27, Sect. 8]. This works essentially in the same way, but the details are slightly more intricate. As a result, we get a structure for sampling a random disk intersecting a given disk, from which our disk reveal structure is readily derived.

### A Deeper Dive into Kaplan et al.

We describe the details of the data structure by Kaplan et al. [27]. Essentially, they explain how to maintain the lower envelope of a set of *xy*-monotone surfaces in $\mathbb {R}^3$ under insertions and deletions, while supporting vertical ray shooting queries [27, Sect. 8]. Their structure is an extension of a structure by Chan [11] that applies to the special case of planes.

The main ingredient are *vertical* *k-shallow* (1/*r*)*-cuttings*: let $\mathcal {A}(H)$ be the arrangement of a set of planes *H* in $\mathbb {R}^3$. The *k-level* $L_k$ of $\mathcal {A}(H)$ is the closure of all points in $\bigcup _{h \in H} h$ with *k* planes of *H* strictly below them. Then, $L_{\le k}(H)$ (or $L_{\le k}$ if the planes are clear from the context) is the union of the levels $L_0, \dots , L_k$. A *vertical k-shallow (1/r)-cutting* for *H* is a set $\Lambda$ of pairwise openly disjoint prisms such that (i) the union of the prisms in $\Lambda$ covers $L_{\le k}(H)$; (ii) the interior of each $\tau \in \Lambda$ is intersected by at most |*H*|/*r* planes of *H*; and (iii) every prism is *vertical* (i.e., it consists of a three-dimensional triangle and all points vertically below it, where some or all vertices the triangle may lie at infinity.). The *conflict list* ${{\,\textrm{CL}\,}}(\tau )$ of a prism $\tau \in \Lambda$ is the set of all planes in *H* that cross the interior of $\tau$. The *size* of $\Lambda$ is the number of prisms in $\Lambda$. Given *H*, a vertical $\Theta (|H|/r)$-shallow 1/*r*-cutting for *H* of size *O*(*r*) can be found in time $O(|H| \log r)$, as shown by Chan and Tsakalidis [14].

The data structure of Kaplan et al.@ consists of $O(\log n)$ static *substructures* of exponentially decreasing sizes, where *n* is the current number of planes in *H*. A substructure $\Xi$ is constructed for some initial set $H' \subseteq H$ of $n'$ planes, It consists of a hierarchy $\Lambda _{m +1}, \dots , \Lambda _0$ of $m + 2$ vertical shallow cuttings, with $m = O(\log n')$. These shallow cuttings are obtained as follows: let $\alpha > 0$ and $k_0 \in {\mathbb {N}}$ be appropriate constants, and let $k_j = 2^j k_0$, for $j > 0$. The cutting $\Lambda _{m+1}$ consists of a single prism covering all of $\mathbb {R}^3$ which has $H'$ as its conflict list. Let $H_{m+1} = H'$ and $n_{m + 1} = |H_{m + 1}|$. The remaining cuttings are constructed iteratively: set $H_m = H_{m + 1}$ and $n_m = |H_m|$. For $j = m, \dots , 0$, the cutting $\Lambda _j$ is obtained as a vertical $k_j$-shallow $(\alpha k_j/n_{j})$-cutting for $H_{j}$. The prisms of $\Lambda _j$ cover $L_{\le k_j}(H_{j})$, and each conflict list contains at most $\alpha k_j$ planes of $H_j$. Additionally, the set *Q* of all planes in $H_{j}$ that lie in “too many” conflict lists of the cuttings constructed so far is determined. All planes in *Q* are removed from the conflict lists in $\Lambda _j$ (but not from the higher cuttings, they are just marked there). For the next round $j - 1$, set $H_{j - 1} = H_j \setminus Q$ and $n_{j - 1} = |H_{j - 1}|$, and the construction continues until $j = 0$. We say that the planes in $H_{-1}$ are *stored* in $\Xi$, and the planes in $H' \setminus H_{-1}$ are *pruned* in $\Xi$. One can show that $|H_{-1}| = \Theta (|H'|)$ (see the paper [27] for details). The substructure $\Xi$ needs $O(n' \log n')$ space, and Chan showed how to build it in $O(n' \log n')$ time [12].

The whole data structure is now obtained as follows: first, a substructure $\Xi$ with $H' = H$ is constructed. Since not all planes from *H* may be stored in $\Xi$, let $H'' = H' \setminus H_{-1}$ be the set of pruned planes in $\Xi$. Then, a substructure $\Xi '$ is constructed for $H''$. This is repeated until there are no more pruned planes at the end of an iteration. At the end, every plane from *H* is stored in exactly one substructure, and one can show that $O(\log n)$ substructures are needed until the pruning stops.

Now, we describe how to perform a vertical ray shooting query for $q = (x, y)$. Let $\ell _q$ be the vertical line through *q*. The shallow cutting $\Lambda _0$ of each of the $O(\log n)$ substructures is searched for the prism that intersects $\ell _q$. This takes $O(\log n)$ time in each $\Lambda _0$, using a suitable point location structure (e.g.@ Edelsbrunner et al. [17]). Then, the conflict list of each such prism is scanned for the lowest plane that intersects $\ell _q$, in $O(k_0) = O(1)$ time per prism. To answer the query, the lowest plane overall is returned. It follows that the total time for a vertical ray shooting query is $O(\log ^2 n)$.

**Fig. 13:**
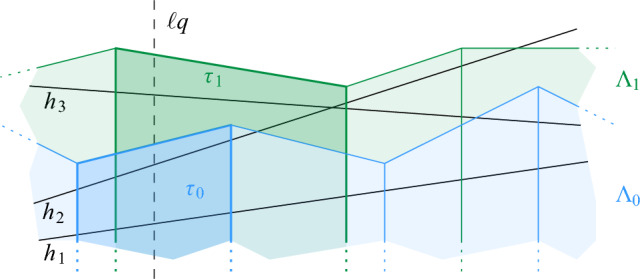
Illustration of a situation, where the lowest active plane of a substructure might not be in the relevant prism. The ray shooting query is performed with $\ell _q$. If $h_1$ and $h_2$ are not active (e.g.@ they were deleted), then $h_3$ has to be returned. As only the conflict list of $\tau _0 \in \Lambda _0$ with $\ell _q \cap \tau _0 \ne \emptyset$ is searched for the lowest active hyperplane, $h_3$ is not found. This issue is mitigated through purging

To insert a new plane a standard Bentley–Saxe-method [5] is used, rearranging smaller substructures into bigger ones to accommodate the new element. Since the Bentley–Saxe-method adds another $\log$-factor over the construction time of a substructure, an insertion needs $O(\log ^2 n)$ amortized time. Deletions are handled lazily, by marking a plane as *deleted*, while leaving it in the structures that contain it. During a query and the rebuilding during an insertion, the deleted planes are simply ignored. Thus, without further action, it may happen that the lowest active (i.e.@ not deleted, not pruned, and not purged, with purging being explained shortly) plane along a given vertical line no longer lies in the relevant prism of a lowest cutting $\Lambda _0$. See Fig. 13 for an example.This is avoided as follows: when deleting a plane *h*, all prisms $\tau$ in the whole structure that have $h \in {{\,\textrm{CL}\,}}(\tau )$ are obtained. For each such $\tau$, a counter is incremented. If this counter reaches the *purging threshold* $\left| {{\,\textrm{CL}\,}}(\tau )\right| /2\alpha$, where $\alpha$ is the constant from the cutting construction above, the prism $\tau$ and all active planes in ${{\,\textrm{CL}\,}}(\tau )$ are marked as *purged*. When a plane is first marked as purged in the substructure where it is stored, it is reinserted into the data structure, using the standard insertion. Naturally, purged planes are ignored during a query and the rebuilding during an insertion.

**Fig. 14:**
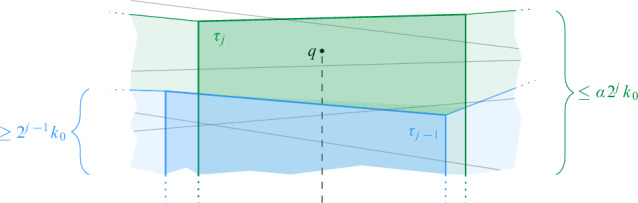
The situation after walking through the shallow cuttings $\Lambda _j$ for a query point $q \in \mathbb {R}^3$ from $j = 0$ upwards. Here, *q* lies in $\tau _j \in \Lambda _j$, but not in $\tau _{j - 1} \in \Lambda _{j - 1}$. Additionally, the number of planes of $H_j$ intersecting the downward vertical ray from *q* is indicated

We briefly outline the reason why the purging mechanism ensures the correctness of the data structure: suppose we would like to perform a vertical ray shooting query $q = (x, y)$, and let $\ell _q$ be the vertical line through *q*. Let *h* be the plane in *H* that has the lowest intersection with $\ell _q$, and let $q^* = \ell _q \cap h$. Furthermore, let $\Xi$ be the substructure that stores *h*, and let $\Lambda _j$ be the lowest shallow cutting (i.e., with minimum index *j*) in $\Xi$ such that $\Lambda _j$ has a prism $\tau$ that contains $q^*$. If $j = 0$, the ray shooting query will be answered correctly. Thus, suppose that $j > 0$. We know that $\left| {{\,\textrm{CL}\,}}(\tau )\right| \le \alpha k_j$, and that $\Lambda _{j-1}$ covers the $k_{j - 1}$-level of $H_{j - 1}$. Hence, $H_{j - 1}$ has at least $k_{j - 1}$ planes that lie vertically below $q^*$; see Fig. 14. By construction, all these planes are also in ${{\,\textrm{CL}\,}}(\tau )$, and since $q^*$ is the answer to the query, all of them must have been deleted from the structure. Thus, at least $k_{j - 1} = k_j/2 \ge \left| {{\,\textrm{CL}\,}}(\tau )\right| /2 \alpha$ planes in ${{\,\textrm{CL}\,}}(\tau )$ have been deleted, so $\tau$ should have been purged already, and *h* would no longer be stored in $\Xi$. This gives a contradiction and we must have $j = 0$. Using an appropriate potential function, one can show that deletions require $O(\log ^4 n)$ amortized time [12]. The overall space requirement of the structure is $O(n \log n)$.

As mentioned above, this approach can also be extended to arrangements of bivariate functions. For the shallow cuttings, the cells are now vertical pseudo-prisms, where the ceiling is a pseudo-trapezoid that lies on a single surface [27, Sect. 3]. One can then use the adapted shallow cuttings as a black box in their data structure for planes to obtain the extended result [27, Thm. 8.3, Thm. 8.4]. Recently, Liu [31, Corr. 4.3] presented an improved construction for the shallow cuttings that leads to fewer $\log$-factors. The analysis is similar to the one sketched above, one simply needs to change a few parameters. Under appropriate, reasonable, conditions on the bivariate functions, the data structure allows insertions in $O(\log ^2 n)$ amortized expected time, deletions in $O(\log ^4 n)$ amortized expected time, and a query requires $O(\log ^2 n )$ time. The data structure requires $O( n \log n )$ space.

### Sampling Planes

We first construct a data structure for the problem of sampling a random disk containing a given point, from a dynamic set of disks. Using standard lifting [3, 42], this is the same as sampling a random plane in $\mathbb {R}^3$ that does not lie above a given three-dimensional query point, from a dynamic set of planes. For this, we extend the data structure by Kaplan et al.

Recall from Sect. 5.1 that their data structure uses $O(\log n)$ substructures $\Xi$, each of which uses a logarithmic number of shallow cuttings $\Lambda _j$, each with an associated set of planes $H_j$. Upon deletion, a plane is only marked as deleted in the respective substructures, and it remains stored there. As explained in Sect. 5.1, the main idea behind the data structure lies in the *purging mechanism* which clears out the conflict list of a prism once too many planes in it are deleted. This ensures that for any query point *q* that lies in a non-purged prism $\tau$, but not in the shallow cutting below it, at least one plane in ${{\,\textrm{CL}\,}}(\tau )$ below *q* is not deleted. We can extend this property of the structure to implement our random sampling query. As described in Sect. 5.1, in the original structure, the conflict list ${{\,\textrm{CL}\,}}(\tau )$ in a prism $\tau$ is purged once $\left| {{\,\textrm{CL}\,}}(\tau )\right| /2\alpha$ planes in ${{\,\textrm{CL}\,}}(\tau )$ have been marked as deleted. To ensure that for any query point *q* that lies in $\tau$, but not in the shallow cutting below it, a constant *fraction* of the non-deleted planes in ${{\,\textrm{CL}\,}}(\tau )$ lie below *q*, we need to lower the purging threshold to $f' = 1/4 \alpha$.^9^ The essential properties of the data structure remain unchanged.

#### Lemma 5.1

Suppose we change the purging threshold in the Kaplan et al.@ data structure for planes  [27, Sect. 7] from $f = 1/2\alpha$ to $f' = 1/4\alpha$. Then, this affects neither the correctness nor the asymptotic time and space bounds of the structure.

The correctness argument in Kaplan et al. [27, Lem. 7.6] (see also Sect. 5.1) is unchanged: the prisms are just purged earlier. The running time analysis [27, Lem. 7.7] requires only an adjustment of constants.^10^ The asymptotic space bound is also unaffected.

#### Lemma 5.2

Suppose we use the purging threshold $f' = 1/4 \alpha$. Let $q \in \mathbb {R}^3$ be a query point, and $\ell _q$ the downward vertical ray from *q*. Let $\Lambda _j$ and $\Lambda _{j-1}$ be two consecutive shallow cuttings in the same substructure, and suppose that $\ell _q$ intersects the prism $\tau _j \in \Lambda _j$ and the prism $\tau _{j - 1} \in \Lambda _{j - 1}$. Assume further that *q* lies in $\tau _j \setminus \tau _{j - 1}$, and that $\tau _j$ has not been purged. Then, $\ell _q$ intersects at least $\left| {{\,\textrm{CL}\,}}(\tau _j)\right| /4\alpha$ planes from ${{\,\textrm{CL}\,}}(\tau _j)$ that are not marked as deleted.

#### Proof

Since $\Lambda _{j - 1}$ covers the $k_{j - 1}$-level of $H_{j - 1}$, it follows that $\ell _q$ intersects at least $k_{j - 1}$ planes from ${{\,\textrm{CL}\,}}(\tau _{j - 1})$. By construction, all these planes are also present in ${{\,\textrm{CL}\,}}(\tau _j)$, see Fig. 14. Furthermore, we have $\left| {{\,\textrm{CL}\,}}(\tau _j)\right| \le \alpha k_j = 2\alpha k_{j - 1}$, as $\Lambda _j$ is an $(\alpha k_j/n_j)$-cutting for $H_j$. Thus, if $\tau _j$ has not been purged, the downward vertical ray $\ell _q$ intersects more than

$$\begin{aligned} k_{j-1} - \frac{\left| {{\,\textrm{CL}\,}}(\tau _j)\right| }{4\alpha } \ge \frac{\left| {{\,\textrm{CL}\,}}(\tau _j)\right| }{2\alpha } - \frac{\left| {{\,\textrm{CL}\,}}(\tau _j)\right| }{4\alpha } = \frac{\left| {{\,\textrm{CL}\,}}(\tau _j)\right| }{4 \alpha } \end{aligned}$$

non-deleted planes from ${{\,\textrm{CL}\,}}(\tau _{j})$. $\square$

To sample a plane not above a given point $q \in \mathbb {R}^3$, we go through the substructures $\Xi$. In each $\Xi$, we walk through the shallow cuttings $\Lambda _j$, from $j = 0$ upwards. In each step, we locate the vertical prism that intersects the downward vertical ray from *q* in $O(\log n)$ time with a suitable point location structure, as it is done in $\Lambda _0$ in the original data structure. If *q* lies *inside* this prism, we stop; otherwise we continue with $\Lambda _{j + 1}$. Finally we apply Lemma 5.2 to the prism we obtain, see Fig. 14.

#### Theorem 5.3

There is a data structure that maintains the lower envelope of a dynamic set of planes in $\mathbb {R}^3$, such that an insertion takes $O(\log ^2 n)$ amortized time, a deletion takes $O(\log ^4 n)$ amortized time, vertical ray shooting queries take $O(\log ^2 n)$ worst-case time, and random sampling queries take $O(\log ^3 n)$ expected time. Here, *n* is the number of planes when the operation is performed. The structure requires $O(n \log n)$ space.

#### Proof

We construct the data structure by Kaplan et al. [27, Sect. 7] with $f' = 1/4 \alpha$ as the purging threshold, and with Chan’s improved algorithm for hierarchies of shallow cuttings [12] and without the space optimization (which we omitted in Sect. 5.1). According to Lemma 5.1, the correctness and asymptotic time and space bounds are unchanged. We construct suitable point location structures [17] for all substructures and all shallow cuttings. This requires $O(n \log n)$ storage, and the running time required to construct these structures is subsumed in the time required to construct the vertical shallow cuttings.

To sample a plane that lies on or below a query point $q \in \mathbb {R}^3$ we proceed as follows: for each substructure $\Xi$, we obtain the first shallow cutting $\Lambda _j$ and the corresponding prism $\tau$ containing *q*, as described above. If $\tau$ was purged, or if $\tau$ is in $\Lambda _0$ and contains no non-deleted plane that intersects the downward vertical ray from *q* (which we can detect in *O*(1) time) we omit $\tau$, otherwise we add it to the set $\Delta$ of *relevant prisms*. This step needs $O(\log ^3 n)$ time in total.

Now, we use rejection sampling as follows: we sample a random relevant prism $\tau$ from $\Delta$, where the sampling probability of a prism $\rho \in \Delta$ is set to $\left| {{\,\textrm{CL}\,}}(\rho )\right| /\sum _{\rho ' \in \Delta } \left| {{\,\textrm{CL}\,}}(\rho ')\right|$. Then, we choose a plane *h* from ${{\,\textrm{CL}\,}}(\tau )$, uniformly at random. If *h* intersects the downward vertical ray from *q* and is not deleted, not purged, and not pruned in $\tau$, we return *h*. Otherwise, we repeat.

By Lemma 5.2, with probability $\Omega (1)$ during one sampling attempt, we obtain an *h* that is not deleted and intersects the downward vertical ray from *q*. Furthermore, the data structure has the property that for every non-deleted plane $h'$, there is exactly one substructure in which $h'$ is neither purged nor pruned. Hence, each sampling attempt returns a valid plane with probability $\Omega (1/\log n)$. Thus, we expect $O(\log n)$ attempts until the sample succeeds. $\square$

Using standard lifting, we get the following corollary:

#### Corollary 5.4

We can implement a data structure that maintains a dynamic set of disks in the plane and allows to sample a random disk from the current set that contains a given query point with the bounds of Theorem 5.3.

### Sampling Disks

To solve the more general problem of sampling a random disk that intersects a given query disk, we adapt the more general data structure of Kaplan et al.@ for continuous bivariate functions of constant description complexity [27, Sect. 8]. This structure works in the same way as the structure for planes, the only difference being that the shallow cutting construction is more general (see also Liu [31, Corr. 4.3] for the improved log-factors). Thus, applying our technique from Sect. 5.2 in the more general setting, we get the following result:

#### Theorem 5.5

Let $\mathcal {F}$ be a set of totally defined continuous bivariate functions of constant description complexity, such that for every finite subset $F \subseteq \mathcal {F}$, the lower envelope of *F* has linear size. Then, a dynamic subset of $\mathcal {F}$ can be maintained while supporting vertical ray shooting queries and random sampling queries with the following guarantees, where *n* is the number of functions in *F* when the operation is performed:

- inserting a function takes $O(\log ^2 n)$ amortized expected time,
- deleting a function takes $O(\log ^4 n)$ amortized expected time,
- a vertical ray shooting query takes $O(\log ^2 n)$ time, and
- sampling a random function that intersects a given downward vertical ray takes $O(\log ^3 n)$ expected time.

The data structure requires $O(n \log n)$ space.

For disks, Theorem 5.5 yields the following:

#### Corollary 5.6

We can implement a data structure that maintains a dynamic set of disks in the plane and can sample a random disk from the current set that intersects a given query disk with the bounds of Theorem 5.5.

#### Proof

Let *D* be a disk with center $c_d$ and radius $r_d$. We can represent the distance of any point $p \in \mathbb {R}^2$ from *D* as an additively weighted Euclidean distance function with $\delta (p, D) = \Vert p c_d\Vert - r_d$. The distance functions for a set of disks form a lower envelope of linear complexity [38]. Hence, we can apply Theorem 5.5 to maintain the distance functions $p \mapsto \delta (p, D)$ for a dynamic set of disks *D*. A disk intersecting a given query disk *Q* with center $c_q$ and radius $r_q$ can then be found by sampling a random function that intersects the downward vertical ray from the point $((c_q)_x, (c_q)_y, r_q)$, as every such function satisfies $\delta (c_q, D) = \Vert c_q c_d\Vert - r_d \le r_q$. $\square$

We can now describe the reveal data structure:

#### Theorem 5.7

(Reveal data structure (RDS)) Let *B* be a set of *n* disks in the plane, and let *R* be initially empty. We can preprocess *B* into a data structure such that the following operations are possible: (i) insert a disk into *R*; (ii) delete a disk from *R*; and (iii) delete a disk from *B* and report all disks from *R* that are revealed by the deletion. It takes $O(\log ^3 n)$ expected time to insert a disk into *R*, and *O*(1) worst-case time to delete a disk from *R*. Preprocessing *B* and performing *k* deletions in *B* requires $O(n \log ^2 n + k \log ^4 n + m \log ^4 n)$ expected time and $O(n \log n + m)$ space, where *m* is the total size of *R* and the *k* deletions are assumed to be oblivious of the internal random choices of the data structure.

#### Proof

We store *B* in the data structure of Corollary 5.6. It takes $O(n \log ^2 n)$ expected time to insert all disks from *B* into the initially empty structure, and the space is $O(n \log n)$.

To insert a new disk *r* into *R*, we pick a random disk $b \in B$ that intersects *r*, and we *assign* *r* to *b*. By Corollary 5.6, this takes $O(\log ^3 n)$ expected time. To delete a disk *r* from *R*, we just remove it from *R* without further action, in *O*(1) time. To delete a disk *b* from *B*, we proceed as follows: we find the set $R_b \subseteq R$ of disks from *R* that are assigned to *b*, and for each $r \in R$, we try to sample a new disk $b_r \in B$ that intersects it. If there is no such disk, we report *r* as *revealed*. Otherwise, we reassign *r* to $b_r$. Finally, we delete *b* from the structure. By Corollary 5.6, this takes amortized expected time $O(\log ^4 n + |R_b| \log ^3 n)$.

To bound the total time for the deletions, we fix a sequence of *k* deletions $b_1, b_2, \dots , b_k$ of *B*, which is assumed to be oblivious of the random assignments through sampling. Then, the total deletion time is

$$\begin{aligned} \sum _{i = 1}^{k} \left( \log ^4 n + |R_{b_{i}}| \log ^3 n\right)&= k \log ^4 n + \left( \sum _{i = 1}^{k} |R_{b_{i}}|\right) \log ^3 n\\&= k \log ^4 n + \left( \sum _{r \in R} I_r \right) \log ^3 n, \end{aligned}$$

where $I_r$ denotes the number of times that the blue disk a red disk $r \in R$ is currently assigned to is deleted. To bound the expected value of $I_r$, consider the set $B_r$ of all disks in the original set *B* that intersect *r*. Due to the assumption of obliviousness, for the *i*-th disk $b_i$ in the deletion sequence the probability that *r* is assigned to $b_i$ is at most $1/(|B_r| - i + 1)$, because this is an upper bound on the probability that $b_i$ was chosen when *r* was assigned the previous time to a blue disk. Hence, the expected value of $I_r$ is at most

$$\begin{aligned} \textbf{E}[I_r] \le \sum _{i = 1}^{|B_r|} \frac{1}{|B_r| - i + 1} = O(\log |B_r|) = O(\log n), \end{aligned}$$

and the total expected time for *k* deletions from *B* is $O(k \log ^4 n + m \log ^4 n)$. $\square$

We now state a variant of Theorem 5.7 that will be useful for the decremental connectivity data structures in Sects. 6.2 and 7.3.

#### Corollary 5.8

Let *R* and *B* be two sets of disks in $\mathbb {R}^2$ with $|R| + |B| = n$. We can preprocess $R \cup B$ into a data structure that supports deletions, while detecting all newly revealed disks of *R* after each deletion. Preprocessing needs $O\left( |B|\log ^2 n + |R|\log ^3 n\right)$ expected time and the resulting data structure needs $O(|B| \log |B| + |R|)$ space. Deleting *k* disks from *B* and any number of disks from *R* needs $O\left( k\log ^4 n + |R|\log ^4 n\right)$ expected time, where the *k* deletions are assumed to be oblivious of the internal random choices of the data structure.

## Logarithmic Dependence on $\Psi$

We turn to the semi-dynamic setting, and we show how to reduce the dependency on $\Psi$ from linear to logarithmic. For both the incremental and the decremental scenario, we use the same proxy graph *H* to represent the connectivity in $\mathcal {D}(S)$. The proxy graph is described in Sect. 6.1. In Sects. 6.2 and 6.3 we present the data structures that are based on *H*.

Throughout this section, we assume that we work with a quadforest on a globally aligned grid. Refer to Appendix A.1 for a details description of how the data structure can be adapted to work on the realRAM without global alignment.

### The Proxy Graph

To define the proxy graph *H*, we use the set *S* of sites, together with a set $\mathcal {A}$ of planar regions, to be described below. The proxy graph *H* has one *site-vertex* for every site in *S* and one *region-vertex* for every region in $\mathcal {A}$. The regions in $\mathcal {A}$ are derived from a quadforest for *S*, and every region $A \in \mathcal {A}$ has two associated sets of sites, $S_1(A)$ and $S_2(A)$. The first set $S_1(A) \subseteq S$ has the property that (i) all sites of $S_1(A)$ lie in *A*; (ii) every site $s \in S_1(A)$ has a radius $r_s$ “comparable” to the diameter of *A*; and (iii) the induced disk graph $\mathcal {D}(S_1(A))$ is a clique. A site *s* can lie in more than one set $S_1(A)$. The second set $S_2(A)\subseteq S$ contains all sites $s \in S$ that (i) lie in the quadtree cell that is “associated” with the region *A*; (ii) have a “small” radius relative to the diameter of *A*; and (iii) are adjacent in $\mathcal {D}(S)$ to at least one site in $S_1(A)$.

The proxy graph *H* is bipartite, with all edges going between the site-vertices for *S* and the region-vertices for $\mathcal {A}$. More precisely, a region-vertex $A \in \mathcal {A}$ is connected to all site-vertices in $S_1(A) \cup S_2(A)$. The edges between *A* and $S_1(A)$ constitute a sparse representation of the clique $\mathcal {D}(S_1(A))$ in $\mathcal {D}(S)$.

The edges between *A* and sites in $S_2(A)$ allow us to represent all edges in $\mathcal {D}(S)$ between sites in $S_1(A)$ and sites in $S_2(A)$ by a path of length at most two in *H*. This representation does not change the connectivity between the site vertices in *H*, as compared to $\mathcal {D}(S)$, since $\mathcal {D}(S_1(A))$ is a clique. We will ensure that every edge in $\mathcal {D}(S)$ is represented in *H* in this manner. Furthermore, the number of regions in $\mathcal {A}$, as well as the total size of the associated sets $S_1(A)$ and $S_2(A)$ will be “small”, resulting in a sparse proxy graph *H*.

We now describe the details of the construction. The graph *H* has vertex set $S \cup \mathcal {A}$, where *S* are the sites and $\mathcal {A}$ is a set of *regions*. For every site $s \in S$, let $\sigma _s \in \mathcal {G}$ be the grid cell of the hierarchical grid with $s \in \sigma _s$ and $|\sigma _s| \le r_s < 2|\sigma _s|$. Let $N(s) = N_{15 \times 15}(\sigma _s)$ be the $15 \times 15$ neighborhood of $\sigma$ on the same level of the hierarchical grid. Then, we let $\mathfrak {N} = (\bigcup _{s \in S} \{ \sigma _s \} \cup N(s))$, and we construct the quadforest $\mathcal {F}$ for $\mathfrak {N}$, as described in Sect. 4.1. Recall that $\mathcal {F}$ contains quadtrees that cover the lowest $\lfloor \log \Psi \rfloor + 1$ levels of the hierarchical grid $\mathcal {G}$. For every cell $\sigma$ of $\mathcal {F}$, we define three kinds of regions: the *outer regions*, the *middle regions*, and the *inner region*.

**Fig. 15:**
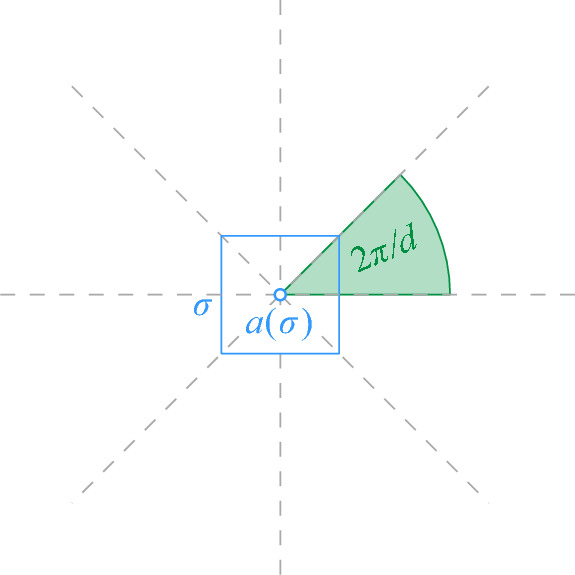
The cones $\mathcal {C}_d$ with angle $2\pi /d$ and apex at the center $a(\sigma )$ of a cell $\sigma$

**Fig. 16:**
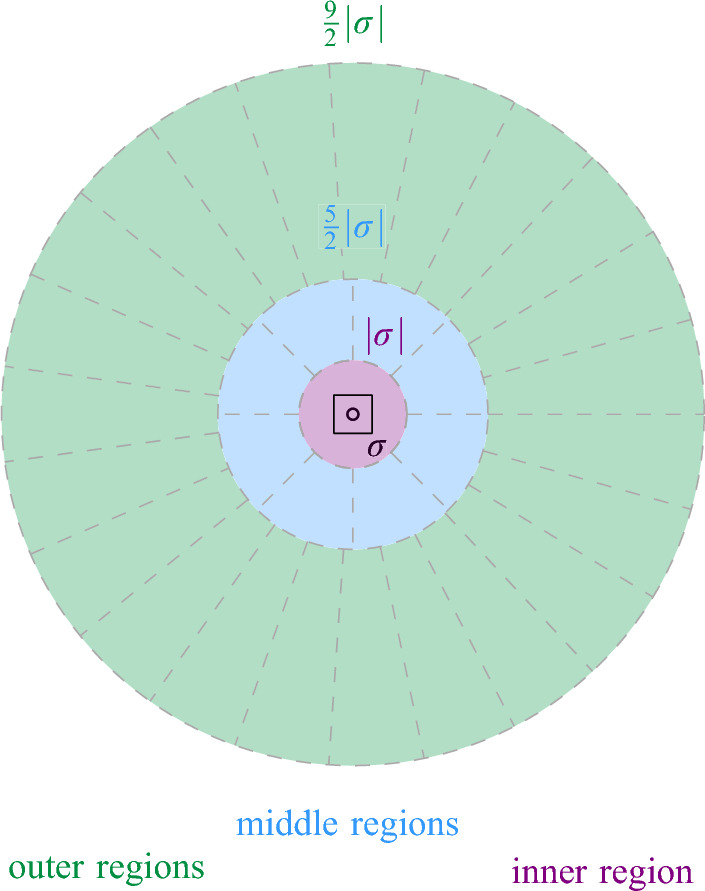
The regions defined by a cell $\sigma$

To describe these regions, we first define for every $d \in {\mathbb {N}}$ a set $\mathcal {C}_d$ of *d* *cones* that have (i) opening angle $2\pi /d$; (ii) the origin as their apex; and (iii) pairwise disjoint interiors. It follows that the cones in $\mathcal {C}_d$ cover the plane. For a cell $\sigma \in \mathcal {F}$, we denote by $\mathcal {C}_d(\sigma )$ the set of all translated copies of the cones in $\mathcal {C}_d$ whose apex has been moved to the center $a(\sigma )$ of $\sigma$, as shown in Fig. 15. To define the *outer regions* for a cell $\sigma \in \mathcal {F}$, we let $d_1 \in {\mathbb {N}}$ be a parameter to be fixed below, and we intersect the cones from $\mathcal {C}_{d_1}(\sigma )$ with the annulus that is centered at $a(\sigma )$ with inner radius $\frac{5}{2}|\sigma |$ and outer radius $\frac{9}{2}|\sigma |$. The *middle regions* of $\sigma$ are defined similarly: we let $d_2 \in {\mathbb {N}}$ be a parameter to be fixed below, and we intersect all cones from $\mathcal {C}_{d_2}(\sigma )$ with the annulus around $a(\sigma )$ that has inner radius $|\sigma |$ and outer radius $\frac{5}{2}|\sigma |$. The *inner region* for $\sigma$ is the disk with center $a(\sigma )$ and radius $|\sigma |$. See Fig. 16 for an illustration. We let $\mathcal {A}_{\mathcal {F}}$ be the set of all inner, middle, and outer regions for all cells in $\mathcal {F}$.

**Fig. 17:**
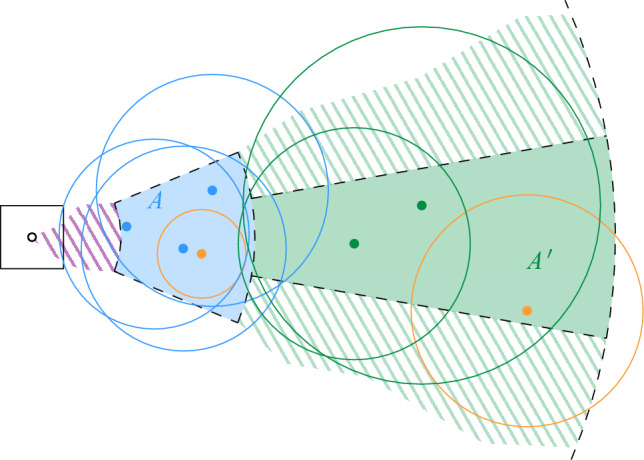
The set $S_1(A)$ is marked blue. The orange site in *A* is not in the set because its radius is too small. The orange site in $A'$ is not in $S_1(A')$: even though its radius is in the correct range, it does not touch or intersect the inner boundary

With every region $A \in \mathcal {A}_\mathcal {F}$, we associate a set of sites $S_1(A) \subseteq S$, as follows:

- if *A* is an outer region for a cell $\sigma$ of $\mathcal {F}$, then the set $S_1(A)$ contains all sites *t* such that (i) $t \in A$; (ii) $|\sigma | \le r_t < 2|\sigma |$; and (iii) $\Vert a(\sigma )t\Vert \le r_t + \frac{5}{2}|\sigma |$. This means that the disk $D_t$ has (i) its center in *A*; (ii) a radius comparable to $|\sigma |$; and (iii) a nonempty intersection with the inner boundary of the annulus used to define *A*.
- if *A* is a middle region or the inner region for a cell $\sigma$ of $\mathcal {F}$, then $S_1(A)$ contains all sites *t* such that (i) $t \in A$; and (ii) $|\sigma | \le r_t < 2|\sigma |$. That is, the disk $D_t$ has (i) its center in *A* and (ii) a radius comparable to $|\sigma |$.

**Fig. 18:**
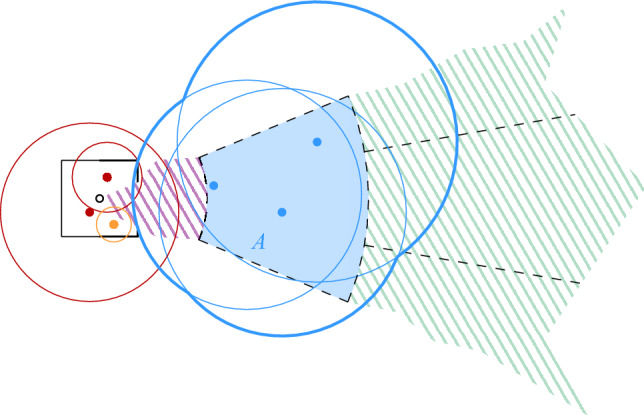
The red sites in $\sigma$ are in $S_2(A)$. The radius of the orange site is in the correct range, but it does not intersect a site in $S_1(A)$ (marked blue)

We define $\mathcal {A} \subseteq \mathcal {A}_\mathcal {F}$ as the set of regions *A* with $S_1(A) \ne \emptyset$. For every $A \in \mathcal {A}$, we define $S_2(A) \subseteq S$ as the set of all sites *s* that (i) lie in $\sigma$; (ii) have radius $r_s < 2|\sigma |$; and (iii) are adjacent in $\mathcal {D}(S)$ to at least one site in $S_1(A)$, see Fig. 18.

We add an edge *sA* in *H* between a site *s* and a region *A* if and only if $s\in S_1(A) \cup S_2(A)$. Note that the sets $S_1(A)$ and $S_2(A)$ are not necessarily disjoint, as for a center region *A* defined by a cell $\sigma$, a site *s* with $s \in \sigma$ and $|\sigma |\le r_s < 2|\sigma |$ will satisfy the conditions for both $S_1(A)$ and $S_2(A)$. However, this will adversely affect neither the preprocessing time nor the correctness. The following structural lemma helps us to show that *H* accurately represents the connectivity in $\mathcal {D}(S)$ as well as to bound the size of *H* and the preprocessing time in the decremental setting.

#### Lemma 6.1

Let $t \in S$ be a site. Then

1. all cells that define a region *A* with $t\in S_1(A)$ are in *N*(*t*); and
2. if $s \in S$ is a site with $r_s \le r_t$ such that *st* is an edge in $\mathcal {D}(S)$, then there is a cell $\sigma \in N(t)$ such that $s \in \sigma$ and such that $\sigma$ defines a region *A* with $t \in S_1(A)$.

**Fig. 19:**
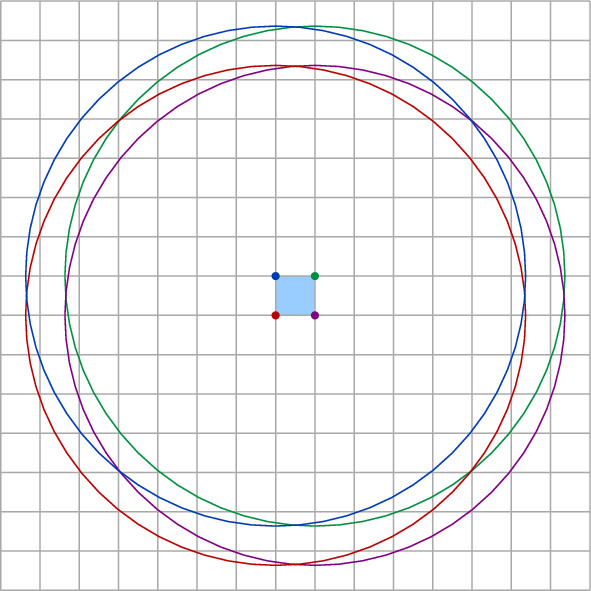
The disk $D(t, \frac{9}{2}|\sigma _t|)$ is contained in $N_{15\times 15}(\sigma _t)$

#### Proof

To prove the first claim, we observe that by definition, all cells $\sigma$ that define a region *A* with $t \in S_1(A)$ have $|\sigma | = |\sigma _t|$ and centers $a(\sigma )$ in the disk $D = D\left( t,\frac{9}{2}|\sigma _t|\right)$. The disk *D* is contained in *N*(*t*) (see Fig. 19), and the claim follows.

We now proceed to the second claim. Since *st* is an edge in $\mathcal {D}(S)$, we have $\Vert st\Vert \le r_s + r_t$. By the definition of $\sigma _t$ and since $r_s \le r_t$, we have $r_s \le r_t < 2 |\sigma _t|$. Now, let $\sigma \in \mathcal {G}$ be the cell with $|\sigma | = |\sigma _t|$ and $s\in \sigma$. Then, the triangle inequality shows

1 $$\begin{aligned} \Vert a(\sigma ) t\Vert \le \frac{1}{2}|\sigma |+ r_s + r_t< \frac{5}{2}|\sigma | + r_t < \frac{9}{2}|\sigma |. \end{aligned}$$

Thus, $a(\sigma )$ lies in the disk $D=D\left( t,\frac{9}{2}|\sigma |\right)$, and $\sigma \in N(t)$, By symmetry, we also have $t\in D\left( a(\sigma ),\frac{9}{2}|\sigma |\right)$, and hence $t \in S_1(A)$. Note that $A$ can be an inner, middle or outer region. The inequalities in (1) also show that the $S_1$-condition (iii) for an outer region is fulfilled. $\square$

Before we argue that *H* accurately represents the connectivity of $\mathcal {D}(S)$, we show that the associated sites of a region in $\mathcal {A}$ form a clique in $\mathcal {D}(S)$.

#### Lemma 6.2

Suppose that $d_1 \ge 23$ and $d_2 \ge 8$. Then, for any region $A \in \mathcal {A}$, the sites in $S_1(A)$ induce a clique in $\mathcal {D}(S)$.

#### Proof

Let $\sigma$ be the cell that defines *A*. We distinguish three cases.

**Fig. 20:**
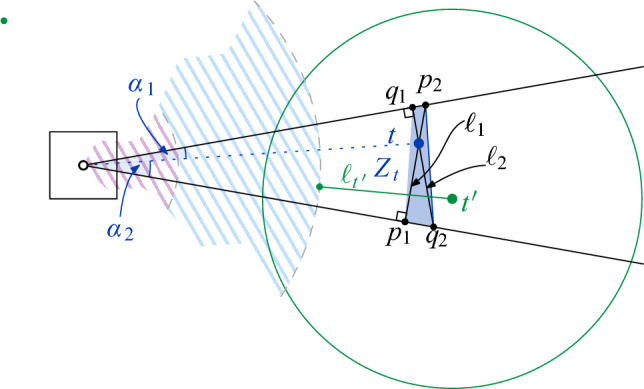
The line segment $\ell _{t'}$ intersects $p_2q_2$

**Case 1:** *A* **is an outer region.** Let $t \in S_1(A)$, and let $C \in \mathcal {C}_{d_1}(\sigma )$ be the cone that was used to define *A*. Consider the line segments $\ell _1$ and $\ell _2$ that that go through *t*, lie in *C*, and are perpendicular to the upper and the lower boundary of *C*, respectively. We denote the endpoints of $\ell _1$ by $p_1$ and $p_2$, and the endpoints of $\ell _2$ by $q_1$ and $q_2$, where $p_1$ and $q_1$ are the endpoints at the right angles; see Fig. 20.

Now, let $Z_t$ be the convex hull of $p_1$, $p_2$, $q_1$, and $q_2$. We claim that $Z_t \subset D_t$. Indeed, let $\alpha _1$, $\alpha _2$ be the angles at $a(\sigma )$ defined by the line segment $\overline{a(\sigma )t}$ and the two boundaries of *C*. By our choice of *C*, we have $\alpha _1 + \alpha _2 = 2\pi /d_1$.

Basic trigonometry yields

$$\begin{aligned} \Vert a(\sigma )p_1 \Vert&= \cos (\alpha _2) \cdot \Vert a(\sigma ) t\Vert ,&\Vert p_1p_2 \Vert&= \Vert a(\sigma )p_1\Vert \cdot \tan \left( 2\pi /d_1\right) ,\\ \Vert a(\sigma )q_1 \Vert&= \cos (\alpha _1) \cdot \Vert a(\sigma ) t\Vert , \text { and }&\Vert q_1q_2 \Vert&= \Vert a(\sigma )q_1\Vert \cdot \tan \left( 2\pi /d_1\right) . \end{aligned}$$

Hence,

$$\begin{aligned} \Vert \ell _1\Vert&= \Vert p_1p_2\Vert = \cos (\alpha _2) \cdot \Vert a(\sigma ) t\Vert \cdot \tan \left( 2\pi /d_1\right) \\ \Vert \ell _2\Vert&= \Vert q_1q_2\Vert = \cos (\alpha _1) \cdot \Vert a(\sigma ) t\Vert \cdot \tan \left( 2\pi /d_1\right) \end{aligned}$$

By $|\cos (\alpha _1)|, |\cos (\alpha _2)| \le 1$, the properties $\Vert a(\sigma )t\Vert \le r_t + (5/2)|\sigma |$ and $|\sigma | \le r_t$ in the definition of $S_1(A)$, and our choice of $d_1$, we get

$$\begin{aligned} \Vert \ell _1 \Vert , \Vert \ell _2\Vert&\le \Vert a(\sigma ) t\Vert \tan \left( \frac{2\pi }{d_1}\right) \le \left( r_t + \frac{5}{2}|\sigma |\right) \cdot \tan \left( \frac{2\pi }{d_1}\right) \\&\le \frac{7}{2} r_t \cdot \tan \left( \frac{2\pi }{d_1}\right) \le r_t. \end{aligned}$$

Since *t* lies on $\ell _1$ and $\ell _2$, it follows that $Z_t \subset D_t$, as claimed.

Next, let $\ell _t = a(\sigma )t \setminus D\left( a(\sigma ),\frac{5}{2}|\sigma |\right)$ be the part of the line segment between *t* and $a(\sigma )$ that lies outside of $D\left( a(\sigma ), \frac{5}{2}|\sigma |\right)$. By the property $\Vert a(\sigma )t \Vert \le r_t + \frac{5}{2}|\sigma |$ in the definition of $S_1(A)$, we have that $\ell _t \subset D_t$.

Consider two sites $t, t' \in S_1(A)$. We argue that $D_t$ and $D_{t'}$ intersect and hence *t* and $t'$ are adjacent in $\mathcal {D}(S)$. Indeed, if $t' \in Z_t \subset D_t$ or $t \in Z_{t'} \subset D_{t'}$, this is immediate. Thus, suppose that this does not hold, and assume without loss of generality that $\Vert a(\sigma )t\Vert \le \Vert a(\sigma )t'\Vert$. As $\Vert a(\sigma ) t\Vert \ge \frac{5}{2}|\sigma |$, the line segment $p_2q_2$ lies completely outside of $D\left( a(\sigma ), \frac{5}{2}|\sigma |\right)$, so $t'$ is separated in *C* from $a(\sigma )$ by the convex hull $Z_t$. Then, $\ell _{t'} \subset D_{t'}$ intersects $p_2q_2 \subset D_t$, as claimed.

**Case 2:** *A* **is a middle region.** The law of cosines and our choice of $d_2$ yield

$$\begin{aligned} {{\,\textrm{diam}\,}}(A) \le |\sigma |\cdot \sqrt{1 + \frac{25}{4} - \frac{10}{2}\cos \left( \frac{2\pi }{d_2}\right) } \le 2|\sigma |. \end{aligned}$$

Now, let $t, t' \in S_1(A)$. By the properties in the definition of $S_1(A)$, we have $t,t'\in A$ and $r_t, r_{t'} \ge |\sigma |$. Thus, we get

$$\begin{aligned} \Vert tt'\Vert \le {{\,\textrm{diam}\,}}(A) \le 2|\sigma | \le r_t + r_{t'}, \end{aligned}$$

and $tt'$ is an edge in $\mathcal {D}(S)$.

**Case 3:** *A* **is an inner region.** Let $t, t' \in S_1(A)$. Since *t* and $t'$ both lie in the disk $D\left( a(\sigma ), |\sigma |\right)$ of diameter $2|\sigma |$, and since $r_t, r_{t'} \ge |\sigma |$, we again get that $tt'$ is an edge in $\mathcal {D}(S)$. $\square$

We can now show that *H* accurately represents the connectivity of $\mathcal {D}(S)$.

#### Lemma 6.3

Two sites are connected in *H* if and only if they are connected in $\mathcal {D}(S)$.

#### Proof

Let $s, t \in S$. First, we show that if *s* and *t* are connected in *H*, they are also connected in $\mathcal {D}(S)$. The path between *s* and *t* in *H* alternates between vertices in *S* and vertices in $\mathcal {A}$. Thus, it suffices to show that if two sites *u* and *u*’ are adjacent in *H* to the same region $A \in \mathcal {A}$, they are connected in $\mathcal {D}(S)$. This follows directly from Lemma 6.2: if $u, u' \in S_1(A)$, they are part of a clique, and hence adjacent. If $u \in S_2(A)$, then there is at least one site in $S_1(A)$ whose disk intersects $D_u$, and hence *u* is connected to all sites in the clique $S_1(A)$. Thus, if $u' \in S_1(A)$, we are done, and if $u' \in S_2(A)$, the same argument shows that $u'$ must also be connected to all sites in $S_1(A)$, and hence *u* and $u'$ are connected through $S_1(A)$.

Now, we consider two sites that are connected in $\mathcal {D}(S)$, and we show that they are also connected in *H*. It suffices to show that if *s* and *t* are adjacent in $\mathcal {D}(S)$, they are connected in *H*. Assume without loss of generality that $r_s \le r_t$, and let $\sigma$ be the cell in *N*(*t*) with $s\in \sigma$. The cell $\sigma$ exists by Lemma 6.1, it belongs to $\mathcal {F}$, since $\sigma$ lies in the first $\lfloor \log \Psi \rfloor + 1$ levels of $\mathcal {G}$, and we have $\sigma _s \subseteq \sigma$. From Lemma 6.1 also follows, that $t \in S_1(A)$ for some $A\in \mathcal {A}_\mathcal {F}$ defined by $\sigma$. As the regions with non-empty sets $S_1(A)$ are in $\mathcal {A}$, the edge *tA* exists in *H*. Now we argue that $s\in S_2(A)$, and thus the edge *As* also exists in *H*. This follows by straightforward checking of the properties from the definition of $S_2(A)$: (i) by the choice of $\sigma$, we have $s\in \sigma$; (ii) by the definition of *N*(*t*) and the assumption $r_s \le r_t$, we have $r_s < 2|\sigma |$; and (iii) we already know that $t \in S_1(A)$, and *s* and *t* are adjacent in $\mathcal {D}(S)$. Thus, *s* and *t* are connected in *H* through *A*, and the claim follows. $\square$

Finally, we show that the size of *H* depends only on *n* and $\Psi$, and not on the number of edges in $\mathcal {D}(S)$ or the diameter of *S*. We first bound the total size of the sets $S_1(A)$ and $S_2(A)$.

#### Lemma 6.4

We have $\sum _{A\in \mathcal {A}} |S_1(A)| = O(n)$ and $\sum _{A \in \mathcal {A}} |S_2(A)| = O(n\log \Psi )$.

#### Proof

First, we bound the total size of the sets $S_1(A)$. Fix a site $t \in S$. By Lemma 6.1, the site *t* can lie only in regions *A* that are defined by cells in *N*(*t*), and there are *O*(1) such cells. Thus, *t* lies in at most *O*(1) sets $S_1(A)$, and since *t* was an arbitrary site, we get $\sum _{A\in \mathcal {A}} |S_1(A)| = O(n)$. Next, we focus on the total size of all sets $S_2(A)$. Fix a site $s \in S$. A necessary condition for $s \in S_2(A)$ is that *s* lies in the cell that defines *A*. There are at most $\lfloor \log \Psi \rfloor + 1$ cells containing $s$ in $\mathcal {F}$, and every such cell defines *O*(1) regions *A*. Since *s* was arbitrary, it follows that $\sum _{A\in \mathcal {A}} |S_2(A)| = O(n\log \Psi )$. $\square$

#### Corollary 6.5

The proxy graph *H* has *O*(*n*) vertices and $O(n\log \Psi )$ edges.

#### Proof

There are at most $\sum _{A\in \mathcal {A}} |S_1(A)|$ non-empty regions *A*, so $|\mathcal {A}| = O(n)$. This gives the bound on the number of vertices. The number of edges is at most $\sum _{A\in \mathcal {A}} |S_1(A)| + |S_2(A)| = O(n\log \Psi )$. $\square$

### Decremental Data Structure

**Fig. 21:**
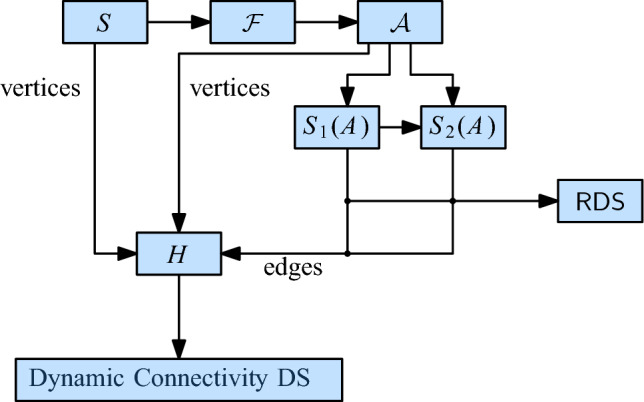
The structure of the decremental data structure

We use the proxy graph *H* from Sect. 6.1 to build a data structure that allows interleaved deletions and connectivity queries in a disk graph. The data structure has several components: we store a quadforest that contains the cells defining $\mathcal {A}$, and for every $A \in \mathcal {A}$, we store the sets $S_1(A)$ and $S_2(A)$. For each region $A\in \mathcal {A}$, we store a reveal data structure (RDS) as in Corollary 5.8 with $B = S_1(A)$ and $R = S_2(A)$. Finally, we store the proxy graph *H* in an HLT-structure $\mathcal {H}$ [23]. See Fig. 21 for an illustration.

As usual, the connectivity queries are answered through $\mathcal {H}$. To delete a site *s*, we first remove from $\mathcal {H}$ all edges incident to *s*. Then, we go through all regions *A* with $s\in S_1(A)$. We remove *s* from $S_1(A)$ and from the RDS of *A*. Let *U* be the set of revealed sites from $S_2(A)$ that are reported by the RDS. We delete every $u\in U$ from $S_2(A)$ and from the corresponding RDS. Additionally, we delete the edges *uA* from $\mathcal {H}$ for all $u\in U$ that are not also in $S_1(A)$. Next, for each region *A* with $s\in S_2(A)$, we simply remove *s* from the set $S_2(A)$ and the associated RDS. First, we analyze the preprocessing time.

#### Lemma 6.6

Given a set *S* of *n* sites, we can construct the data structure described above in $O\left( n\log \Psi \log ^3 n\right)$ time. The data structure requires $O(n (\log n + \log \Psi ))$ space.

#### Proof

For each $s \in S$, we identify the cell $\sigma _s$ of $\mathcal {G}$ that has $s \in \sigma _s$ and $|\sigma _s| \le r_s < 2 |\sigma _s|$. Let $\mathfrak {N} = \bigcup _{s \in S} (\{ \sigma _s \} \cup N(s))$.^11^ We build the quadforest $\mathcal {F}$ for $\mathfrak {N}$, together with a red-black tree [15] that contains the roots, as described in Sect. 4.1. As in Sect. 4.1, the cells of $\mathcal {F}$ lie on the first $\lfloor \log \Psi \rfloor + 1$ levels of $\mathcal {G}$, and it takes $O(n(\log \Psi + \log n))$ steps to construct it.

Now we find the sets $S_1(A)$ and $S_2(A)$. Fix a site $t \in S$. By Lemma 6.1, we can find all sets $S_1(A)$ that contain *t* by looking at the regions defined by cells in *N*(*t*). Thus, we find *N*(*t*), and for every $\sigma \in N(t)$, we iterate over all regions *A* defined by $\sigma$, identifying those with $t \in S_1(A)$. Identifying the cells in *N*(*t*) takes $O(\log n + \log \Psi )$ time per cell and also overall, since *N*(*t*) has constant size. Thus, the sets $S_1(A)$ can be constructed in total time $O(n(\log n + \log \Psi ))$

To find the sets $S_2(A)$, we build an *additively weighted Voronoi diagram* for each set $S_1(A)$, where a site $t\in S_1(A)$ has weight $-r_t$. Such a diagram can be constructed in $O\left( |S_1(A)|\log n\right)$ time, and additively weighted nearest-neighbor queries in $S_1(A)$ then take $O(\log n)$ time [16, 20, 38]. By Lemma 6.4, we need $O(\sum _{A\in \mathcal {A}} |S_1(A)| \log n) = O(n\log n)$ time to compute all the diagrams. For a site $s\in S$, let $\pi _s$ be the path in $\mathcal {F}$ from the root to the cell $\sigma _s$. For each cell along $\pi _s$, we query all additively weighted Voronoi diagrams for its regions *A* with *s*. If *s* intersects the reported nearest neighbor, we add *s* to $S_2(A)$. A site *s* is used for $O(\log \Psi )$ queries, for an overall of $O(n\log n\log \Psi )$ time to find all sets $S_2(A)$.

The edges of *H* are determined by the sets $S_1(A)$ and $S_2(A)$. We insert the edges into an initially empty HLT-structure, so we obtain the connectivity data structure $\mathcal {H}$ in overall $O\left( n\log \Psi \log ^2 n\right)$ time. Following Corollary 6.5, $\mathcal {H}$ requires $O(n \log \Psi )$ space.

For every region $A \in \mathcal {A}$, we build the RDS with $B = S_1(A)$ and $R = S_2(A)$ in $O\left( |S_1(A)|\log ^2 n + |S_2(A)|\log ^3 n \right)$ expected time, using Corollary 5.8. Each of the resulting RDS requires $O(|S_1(A)| \log |S_1(A)| + |S_2(A)|)$ space. Summing over all regions and using Lemma 6.4, we get a total expected time of

$$\begin{aligned}&O\left( \sum _{A\in \mathcal {A}} |S_1(A)|\log ^2 n +|S_2(A)|\log ^3 n \right) \\&\quad = O\left( \left( \sum _{A\in \mathcal {A}} |S_1(A)|\right) \cdot \log ^2 n + \left( \sum _{A\in \mathcal {A}}|S_2(A)|\right) \cdot \log ^3 n \right) \\&\quad =O\left( n\log ^2 n + n \log \Psi \log ^3 n \right) = O(n \log \Psi \log ^3 n), \end{aligned}$$

and this dominates the preprocessing time.

Similarly, summing over all regions and using Lemma 6.4 yields a total space usage for the reveal data structures of

$$\begin{aligned}&O\left( \sum _{A\in \mathcal {A}} |S_1(A)| \log |S_1(A)| + |S_2(A)| \right) \\&\quad = O\left( \left( \sum _{A\in \mathcal {A}} |S_1(A)| \right) \log n + \left( \sum _{A\in \mathcal {A}} |S_2(A)| \right) \right) \\&\quad = O(n (\log n + \log \Psi )), \end{aligned}$$

which dominates the space usage for the regions and their associated sets, the quadforest $\mathcal {F}$, the HLT-structure $\mathcal {H}$, and also the temporarily constructed additively weighted Voronoi diagrams.

$$\square $$

Now we show that the data structure correctly and efficiently handles queries and deletions.

#### Theorem 6.7

The data structure handles deletions of sites in overall expected time $O\left( n\log \Psi \log ^4n \right)$, assuming the deletions are oblivious of the internal random choices of the data structure. Furthermore, it requires $O(\log n/\log \log n)$ time to answer connectivity queries correctly and requires $O(n (\log n + \log \Psi ))$ space.

#### Proof

We first show that the answers given by our data structure are indeed correct. Over the lifetime of the data structure, we maintain the invariant that the sets $S_1(A)$ and $S_2(A)$ always contain the sites as defined in Sect. 6.1, the graph stored in $\mathcal {H}$ is the proxy graph *H*, and each RDS associated with a region *A* contains the sets $S_1(A)$ and $S_2(A)$. Assuming that this invariant holds, Lemma 6.3 implies that the connectivity queries are answered correctly.

To show that the invariant is maintained when deleting a site *s*, we first note that removing *s* from a set $S_2(A)$ only leads to the deletion of a single edge in *H*. As we make sure to mirror the removal from $S_2(A)$ in $\mathcal {H}$ and the RDS, removing *s* from all sets $S_2(A)$ that contain it maintains the second half of the invariant.

Now, let *A* be a region such that *s* lies in $S_1(A)$. Then, for all sites *t* in the corresponding set $S_2(A)$, it is necessary that *t* intersects at least one site in $S_1(A)$. Furthermore, there is a—possibly empty—set $U'$ of sites in $S_2(A)$ that only intersect *s*. So to maintain the invariant, we have to delete $U'$ from $S_2(A)$ and the associated RDS. As $U'$ contains exactly the sites reported in the set *U* returned by the RDS, the sites from $U'$ are removed by construction, and the invariant on $S_2(A)$ and the RDS is maintained. Since we do not delete the edges that were present because a site was in $S_1(A) \cap S_2(A)$, the graph stored in $\mathcal {H}$ is still the proxy graph *H*, and the invariant is maintained.

By these observations, applied to $S_1(A)$ and $S_2(A)$ for all regions *A* during a deletion, and by the fact that a deletion removes all edges incident to the deleted site, the invariant holds.

Now, we analyze the running time. Queries are performed to $\mathcal {H}$ take $O(\log n/\log \log n)$ time. Every edge is removed exactly once from $\mathcal {H}$, for a total of $O(n\log \Psi \log ^2n)$ time. Finding the regions *A* whose sets $S_1(A)$ and $S_2(A)$ have to be updated during a deletion takes again $O(\log n+\log \Psi )$ time, by similar argument as in the proof of Lemma 6.6. The running time is dominated by the deletions from the RDS. By Corollary 5.8, the RDS associated with a single region adds $O\left( |S_1(A)| \log ^2 n + k_A \log ^{4} n + |S_2(A)|\log ^4 n\right)$ expected steps to the total running time, where $k_A$ is the number of sites deleted from $S_1(A)$ and the deletions are assumed to be oblivious of the internal random choices of the RDS . Summing over all regions, we have $\sum _{A\in \mathcal {A}} k_A = O(k)$, since every site is contained in *O*(1) sets $S_1(A)$. Furthermore, as we have $\sum _{A\in \mathcal {A}} |S_1(A)| = O(n)$ and $\sum _{A\in \mathcal {A}} |S_2(A)| = O(n\log \Psi )$, an overall running time of

$$\begin{aligned} O\left( n\log ^2 n + k \log ^{4}n + n\log \Psi \log ^4 n \right) = O\left( n\log \Psi \log ^4 n + k \log ^{4} n\right) \end{aligned}$$

for *k* deletions follows. As $k \in O(n\log \Psi )$ and the the space usage is unchanged from Lemma 6.6, the theorem follows. $\square$

### Incremental Data Structure

Next, we describe our incremental connectivity data structure for the bounded radius ratio case. It is also based on the proxy graph *H* from Sect. 6.1. Since we only do insertions, we use the connectivity data structure from Theorem 2.1 for $\mathcal {H}$. This data structure achieves $O\left( 1\right)$ amortized time for updates and $O\left( \alpha (n)\right)$ amortized time for queries.

**Fig. 22:**
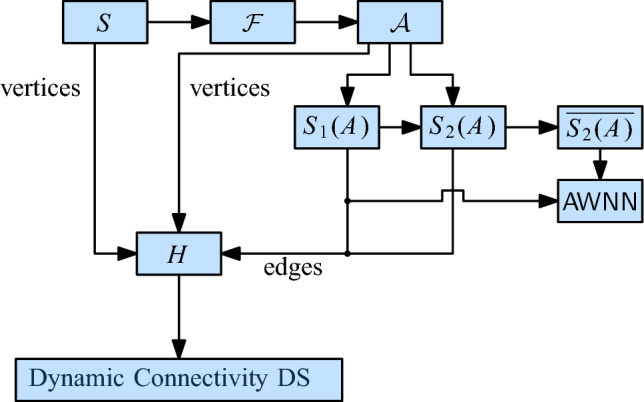
The structure of the incremental data structure

To update the edges incident to a region *A* defined by a cell $\sigma$, we use two fully dynamic additively weighted nearest neighbor data structures (AWNN, Lemma 2.4): one for the set $S_1(A)$ and one for the set $\overline{S_2(A)}$ that contains those sites $s \in \sigma$ with radius $r_s < 2|\sigma |$ that have not been added to $S_2(A)$ yet. As before, we maintain a quadforest $\mathcal {F}$ of height $\lfloor \log \Psi \rfloor + 1$ to navigate the cells. See Fig. 22 for an illustration of the data structure.

The data structure works as follows: when inserting a site *s*, we determine the cells of the neighborhood *N*(*s*), and add those among then that are not in $\mathcal {F}$ to $\mathcal {F}$. Furthermore, we add the associated region vertices to *H* and also to the dynamic connectivity graph structure $\mathcal {H}$. Then, we have to connect the site *s* to the regions. Hence, we have to identify the sets $S_1(A)$, $S_2(A)$, and $\overline{S_2(A)}$ that the site *s* belongs to, add *s* to the corresponding AWNN s, and insert the edges incident to *s* into $\mathcal {H}$. After the insertion of *s* into a set $S_1(A)$, we also query the AWNN of the associated set $\overline{S_2(A)}$ to find possible sites that intersect $D_s$ and thus have to be moved to $S_2(A)$, do so if required, and add the edges incident to the transferred site to $\mathcal {H}$.

#### Theorem 6.8

The data structure described above correctly answers connectivity queries in $O(\alpha (n))$ amortized time, performs insertions in $O(\log \Psi \log ^{4} n)$ amortized expected time, and requires $O(n \log \Psi \log n)$ space, where *n* is the number of sites stored in the data structure.

#### Proof

First, we show correctness, using similar invariants as in the proof of Theorem 6.7. For every region *A*,

1. the AWNN of $S_1(A)$ contains exactly the sites in $S_1(A)$,
2. the AWNN $\overline{S_2(A)}$ contains exactly the sites that would lie in $S_2(A)$ if there was a disk in $S_1(A)$ intersecting them,
3. the sets $S_1(A)$ and $S_2(A)$ represented in our data structure always contain the sites as defined in Sect. 6.1; and
4. the graph stored in $\mathcal {H}$ is the proxy graph *H*.

Note that we do not need to store the sets $S_2(A)$ explicitly, since no sites are deleted from $S_2(A)$ and since $S_2(A)$ is not used during the insertion procedure. Thus, the set $S_2(A)$ is implicitly represented by the edges of $\mathcal {H}$. Under the assumption that the invariants hold, Lemma 6.3 again implies that connectivity queries are answered correctly.

Invariant 1 and the first part of Invariant 3 hold by construction. When inserting a site *s*, it is added to the sets $S_1(A)$ of all regions *A* with $A \in N(s)$ that *s* belongs to. By Lemma 6.1, these are the only regions potentially containing *s*. When adding *s* to $S_1(A)$, we also add *s* to the AWNN of $S_1(A)$, and insert the edge *sA* into $\mathcal {H}$. This is exactly the edge we need in *H*. To complete the proof of Invariants 3 and 4, we first need to prove that Invariant 2 holds.

By definition of $S_2(A)$, for a region *A* defined by a cell $\sigma$, we only have in $S_2(A)$ sites *s* with $s \in \sigma$, $r_s < 2|\sigma |$, which satisfy the constraint that $D_s$ intersects at least one site in $S_1(A)$. Furthermore, we add *s* to $\overline{S_2(A)}$, if it fulfills the first two but not the last constraint. This guarantees that a site *s* is potentially present in the sets $S_2(A)$ of the regions defined by any cell on the path from the root to $\sigma _s$, as these are exactly the cells for which the radius constraint of $S_2(A)$ holds. We check for all those regions if *s* intersects any site in $S_1(A)$. The site is only inserted into $S_2(A)$ if there is an intersection, otherwise, it is added to the AWNN of $\overline{S_2(A)}$. Furthermore, a site in $\overline{S_2(A)}$ is only transferred from $\overline{S_2(A)}$ to $S_2(A)$ if a newly inserted site to $S_1(A)$ intersects it. This proves Invariant 2. Whenever we assign a site to a region $S_2(A)$, during its insertion or due to a change of the corresponding set $S_1(A)$, we add the edge *sA* to $\mathcal {H}$, thus representing the edge we need in *H*. This also concludes the proof of Invariants 3 and 4. The correctness follows.

Now, we consider the finer details and the running time. When inserting a site *s*, we first have to identify the root of the quadtree in $\mathcal {F}$ that contains $\sigma _s$. Then, we can descend into this quadtree to find $\sigma _s$, creating new quadtree nodes when necessary, including in the neighborhoods of the cells on the path towards $\sigma _s$. As in Sect. 4.1, this takes $O\left( \log n + \log \Psi \right)$ time, where *n* is the number of sites at the time of the insertion. When introducing new cells into $\mathcal {F}$, we also need their associated regions to be present in the proxy graph *H*. Hence, we insert the regions of the new cells as isolated vertices to $\mathcal {H}$, and we initialize the corresponding AWNN s for $S_1(A)$ and $\overline{S_2(A)}$. Subsequently, we have to insert *s* and its incident edges: first, we add *s* as an isolated vertex to $\mathcal {H}$. Then, we insert *s* into the sets it needs to be contained in and, if applicable, to the associated AWNN s. Since we implicitly store $S_1(A)$ and $S_2(A)$, we add the corresponding edges between the regions and *s* to $\mathcal {H}$ along the way.

Recall that by Lemma 6.1, the site *s* can only be in a set $S_1(A)$ defined by a cell in *N*(*s*). Thus, we iterate through the cells of *N*(*s*) and we add *s* with weight $-r_s$ to the AWNN s of the sets $S_1(A)$ it belongs to. We also introduce the respective edges *sA* into *H*. As *N*(*s*) is of constant size, this step takes *O*(1) amortized time for the insertion of edges in $\mathcal {H}$ and $O(\log ^2 n)$ amortized expected time for insertions into the AWNN s. As the size of $N(s)$ is a constant, this step can be implemented without explicit pointers to neighboring cells by traversing the quadtree from the root to each cell in $N(s)$ without any overhead.

Furthermore, for a region *A*, changes in $S_1(A)$ may affect the set $S_2(A)$. Hence, we have to check if the insertion of *s* into $S_1(A)$ causes a site *t* to move from $\overline{S_2(A)}$ to $S_2(A)$ because $D_s$ intersects $D_t$. We identify these sites by successive weighted nearest-neighbor queries with *s* in the AWNN of $\overline{S_2(A)}$, stopping when the resulting site does not intersect $D_s$. For each site *t* that intersects $D_s$, we delete *t* from the AWNN of $\overline{S_2(A)}$ and insert the edge *tA* into $\mathcal {H}$. The amortized expected running time of $O(\log ^{4} n)$ to delete *t* from $\overline{S_2(A)}$ dominates this step. We do not know how many deletions are performed in this step, but a site *t* can be present in the most $O(\log \Psi )$ sets $\overline{S_2(A)}$, associated to the cells containing *t* on the path from the root to $\sigma _t$. Furthermore, a site *t* is inserted into $\overline{S_2(A)}$ only once. Thus, we can charge those deletions to the previous insertions, which yields an amortized expected running time of $O(\log \Psi \log ^{4} n)$ for this step per insertion.

Now, we consider the time needed to insert *s* either into $S_2(A)$ or $\overline{S_2(A)}$ for some regions *A*. By definition, we know that *s* has to be added either to $S_2(A)$ or to $\overline{S_2(A)}$ of the regions of the cells along the path from the corresponding root to $\sigma _s$ in $\mathcal {F}$. Let *A* be a region defined by a cell along this path. To determine whether to add *s* to $S_2(A)$ or to $\overline{S_2(A)}$, we perform a weighted nearest neighbor query in the AWNN of $S_1(A)$. If we find a site in $S_1(A)$ that intersects $D_s$, we know that *s* belongs to $S_2(A)$. Then, we insert the edge *sA* to $\mathcal {H}$, if it does not exist yet. This takes *O*(1) amortized time. In the other case, *s* is inserted to the AWNN of $\overline{S_2(A)}$, in $O(\log ^2 n)$ amortized expected time. The assignment to $S_2(A)$ or to $\overline{S_2(A)}$ concludes the insertion. Since each cell defines a constant number of regions, the step requires a constant number of lookup and insertion operations on each level. As the time for insertions into the AWNN of $\overline{S_2(A)}$ dominates, we get an amortized expected running time of $O(\log \Psi \log ^2 n)$. Summing over all insertion steps, we achieve an amortized expected running time of $O(\log \Psi \log ^{4} n)$ per insertion, due to the dominating running time of the deletions from $\overline{S_2(A)}$.

The query time follows directly from the query time in $\mathcal {H}$.

Analyzing the space usage of the data structure is similar to the proof of Lemma 6.6. The quadforest $\mathcal {F}$ requires $O(n \log \Psi )$ space, as does $\mathcal {H}$ by Corollary 6.5. This space usage is again dominated by the space required for the AWNN s for $S_1(A)$ and $\overline{S_2(A)}$ for all regions *A*.

First, we have $\sum _{A\in \mathcal {A}} |\overline{S_2(A)}| = O(n \log \Psi )$ for the same reason as in Lemma 6.4. Following this and Lemma 6.4, we get a space usage for the AWNN s of

$$\begin{aligned}&O\left( \sum _{A\in \mathcal {A}} |S_1(A)|\log |S_1(A)| +|\overline{S_2(A)}|\log |\overline{S_2(A)}| \right) \\&\quad = O\left( \left( \sum _{A\in \mathcal {A}} |S_1(A)| +|\overline{S_2(A)}| \right) \cdot \log n \right) \\&\quad = O(n \log \Psi \log n), \end{aligned}$$

which is also the overall space bound. The theorem follows. $\square$

## Arbitrary Radius Ratio

We extend the approach from Sect. 6 to obtain a decremental data structure with a running time that is independent of $\Psi$. The $O\left( n \log \Psi \log ^4n\right)$ term in Theorem 6.7 came from the total size of the sets $S_2(A)$ which, in turn, followed from the height of the quadtrees in $\mathcal {F}$. We can get rid of this dependency by using a *compressed quadtree* $\mathcal {Q}$ instead of $\mathcal {F}$ (see Sect. 7.1). The height and size of $\mathcal {Q}$ do not depend on the radius ratio or the diameter of *S*, but only on *n*. Nonetheless, the height of $\mathcal {Q}$ could still be $\Theta (n)$, which is not favorable for our purposes. In order to reduce the number of edges in our proxy graph to $O\left( n\log ^2n\right)$, we use a *heavy path decomposition* of $\mathcal {Q}$ (see Sect. 7.1) in combination with a *canonical decomposition* of every heavy path. The new proxy graph *H* is described in Sect. 7.2, and the decremental connectivity data structure based on *H* can be found in Sect. 7.3.

### More Preliminaries

Let ${{\,\textrm{diam}\,}}(S) = \max _{s,t\in S} \Vert st \Vert$ be the diameter of the initial site set *S*. To simplify our arguments, we assume without loss of generality that *S* and its associated radii are scaled so that all associated radii are at least 1. This allows us to keep working with the hierarchical grid $\mathcal {G}$ from Sect. 2.3

**Compressed quadtrees.** If we define a quadtree for a set $\mathcal {C}$ of *n* cells as in Sect. 2.3, then it has *O*(*n*) leaves and height $O(\log (|\rho |))$, where $\rho$ is the smallest cell in $\mathcal {G}$ that contains all cells of $\mathcal {C}$. This height can be arbitrarily large, even if *n* is small. To avoid this, we need the notion of a *compressed quadtree* $\mathcal {Q}$ [21]. Let $\mathcal {T}$ be the (uncompressed) quadtree for $\mathcal {C}$. Then, let $\sigma _1, \dots , \sigma _k$ be a maximal path in $\mathcal {T}$ towards the leaves, where all $\sigma _i$, $1 \le i \le k - 1$, have only one child that contains (not necessarily proper) cells from $\mathcal {C}$, and no $\sigma _i$, $2 \le i \le k - 1$ lies in $\mathcal {C}$. In the compressed quadtree $\mathcal {Q}$, this path is replaced by the single edge $\sigma _1 \sigma _k$. Then, $\mathcal {Q}$ has *O*(*n*) vertices, height *O*(*n*), and it can be constructed in $O(n\log n)$ time [7, 21].

**Fig. 23:**
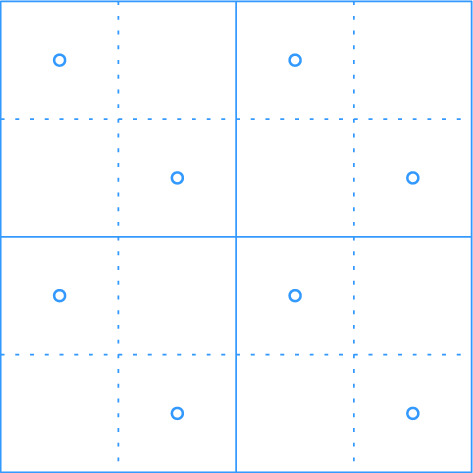
Four cells with the virtual sites to ensure that they are present in the compressed quadtree

Indeed, the latter construction algorithm is stated for planar point sets (and not for cells), but it can be applied by using a set of *O*(*n*) virtual sites, similar to a construction of Har-Peled [21]: for each cell $\sigma \in \mathcal {C}$, we add two virtual sites that lie at the centers of two of the four cells that partition $\sigma$, see Fig. 23. Now, all cells in $\mathcal {C}$ have at least two children in the non-compressed quadtree for the virtual sites, and thus the cells are also present in the compressed quadtree for the virtual sites, as constructed by the traditional algorithms.

The construction of Buchin et al. [7] does not use the floor function at the cost of having cells that are not aligned. In Appendix A.2 we give the details on how to slightly modify the construction process and the operations on the quadtrees such that we can for our purposes assume to use an aligned compressed quadtree with pointers from each cell to its neighbors at each level of the hierarchical grid.

**Heavy paths.** Let *T* be a rooted ordered tree (i.e., we have an order on the children of each interior node of *T*). An edge $uv \in T$ is called *heavy* if *v* is the first child of *u* in the given child-order that maximizes the total number of nodes in the subtree rooted at *v* (among all children of *u*). Otherwise, the edge *uv* is *light*. By definition, every interior node in *T* has exactly one child that is connected by a heavy edge.

A *heavy path* is a maximum path in *T* that consists only of heavy edges. The *heavy path decomposition* of *T* is the set of all the heavy paths in *T*. The following lemma summarizes a classic result on the properties of heavy path decompositions.

#### Lemma 7.1

(Sleator and Tarjan [39]) Let *T* be a tree with *n* vertices. Then, the following properties hold:

1. Every leaf-root path in *T* contains $O(\log n)$ light edges;
2. every vertex of *T* lies on exactly one heavy path; and
3. the heavy path decomposition of *T* can be constructed in *O*(*n*) time.

### The Proxy Graph

The general structure of the proxy graph is as in Sect. 6.1, and we will often refer back to it. We still have a bipartite graph with *S* on one side and a set of regions vertices on the other side. The regions are again used to define sets $S_1(A)$ and $S_2(A)$ that determine the edges. However, we adapt the regions *A* and define them based on certain *subpaths* of the compressed quadtree $\mathcal {Q}$ instead of single cells. Furthermore, we relax the condition on the radii in the definition of the sets $S_1(A)$.

As usual, for a site $s \in S$, let $\sigma _s$ be the cell in $\mathcal {G}$ with $s \in \sigma _s$ and $|\sigma _s| \le r_s < 2|\sigma _s|$. Let *N*(*s*) be the $(15 \times 15)$-neighborhood of $\sigma _s$. Let $\mathfrak {N} = \bigcup _{s \in S} \{ \sigma _s \} \cup N(s)$, and let $\mathcal {Q}$ be the compressed quadtree for $\mathfrak {N}$. Now, let $\mathcal {R}$ be the heavy path decomposition of $\mathcal {Q}$, as in Lemma 7.1. For each heavy path $R \in \mathcal {R}$, we find a set $\mathcal {P}_R$ of *canonical paths* such that every subpath of *R* can be written as the disjoint union of $O(\log n)$ canonical paths. Specifically, for each $R \in \mathcal {R}$, we build a *biased* binary search tree $T_R$ with the cells of *R* in the leaves, sorted by increasing diameter. The weights in the biased binary search tree are chosen as described by Sleator and Tarjan [39]: for a node $\sigma$ of *R*, let the weight $w_\sigma$ be the number of nodes in $\mathcal {Q}$ that are below $\sigma$ (including $\sigma$), but not below another node of *R* below $\sigma$. Then, the depth of a leaf $\sigma$ in $T_R$ is $O(\log (w_R/w_\sigma ))$, where $w_R$ is the total weight of all leaves in $T_R$. We associate each vertex *v* in $T_R$ with the path induced by the cells in the subtree rooted at *v*, and we add this path to $\mathcal {P}_R$. Using this construction, we can write every path in $\mathcal {Q}$ that starts at the root as the disjoint union of $O(\log n)$ canonical paths, as shown in the following lemma:

#### Lemma 7.2

Let $\sigma$ be a vertex of $\mathcal {Q}$, and let $\pi$ be the path from the root of $\mathcal {Q}$ to $\sigma$. There exists a set $\mathcal {P}_\pi$ of canonical paths such that:

1. $$|\mathcal {P}_\pi | = O\left( \log n\right) $$; and
2. $\pi$ is the disjoint union of the canonical paths in $\mathcal {P}_\pi$.

**Fig. 24:**
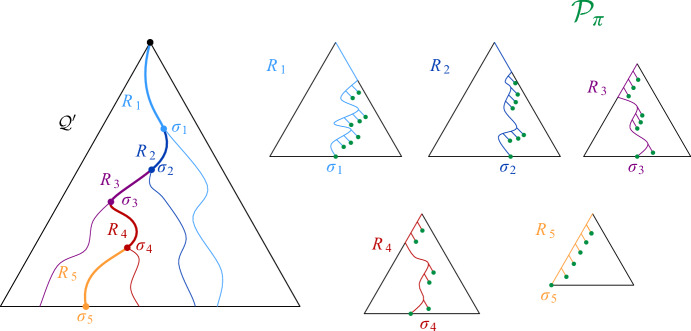
Illustration of Lemma 7.2. On the left, we see the decomposition of $\pi$ into $R_1,\dots R_k$. On the right, the vertices defining $\mathcal {P}_\pi$ are depicted in green

#### Proof

Consider the heavy paths $R_1,\dots , R_k$ encountered along $\pi$. By Lemma 7.1, $k = O(\log n)$, and the path $\pi$ can be subdivided into the intersections $\pi \cap R_i$. Each of these intersections constitutes a subpath of $R_i$ whose largest cell is also the largest cell of $R_i$. Let $\sigma _i$ be the smallest cell of $\pi \cap R_i$. Then, the subpath of $R_i$ consists of all cells in $R_i$ with diameter at least $|\sigma _i|$. This subpath can be composed as the disjoint union of all canonical paths defined by the right children along the search path of $\sigma _i$ in the biased binary search tree associated with $R_i$, together with the path consisting of $\sigma _i$ only; see Fig. 24. Summing the depths of the corresponding leaves of the biased binary search trees, we get that the overall number of canonical paths for $\pi$ is $O(\log n)$. $\square$

The vertex set of the proxy graph *H* again consists of *S* and a set of regions $\mathcal {A}$. We define *O*(1) regions for each canonical path *P* in a similar way as in Sect. 6.1. Let $\sigma$ be the smallest cell and $\tau$ the largest cell of *P*. The *inner* and *middle regions* of *P* are defined as in Sect. 6.1, using $\sigma$ as the defining cell. More precisely, the inner region for *P* is the disk with center $a(\sigma )$ and radius $|\sigma |$. The middle regions of *P* are the $d_2$ regions that are defined as the intersection of the cones in $\mathcal {C}_{d_2}(\sigma )$ with the annulus of inner radius $|\sigma |$ and outer radius $\frac{5}{2}{|\sigma |}$. For the *outer regions* of *P*, we extend the outer radius of the annulus: they are defined as the intersections of the cones in $\mathcal {C}_{d_1}(\sigma )$ with the annulus of inner radius $\frac{5}{2}{|\sigma |}$ and outer radius $\frac{5}{2}|\sigma | + 2|\tau |$, again centered at $a(\sigma )$. The set $\mathcal {A}$ now contains the regions defined in this way for all canonical paths.

Given a region $A\in \mathcal {A}$ for a canonical path *P* with smallest cell $\sigma$ and largest cell $\tau$, we can now define the sets $S_1(A)$ and $S_2(A)$. The set $S_1(A)$ is defined similarly to the analogous set in Sect. 6.1, again using $\sigma$ as the defining cell for most parts. The difference is that the radius range for a site *t* in a set $S_1(A)$ is larger, as its upper bound depends on the diameter of $\tau$. The set $S_1(A)$ contains all sites *t* such that

(i) $$t \in A$$;
(ii) $$|\sigma | \le r_t < 2|\tau |$$; and
(iii) $$\Vert a(\sigma )t\Vert \le r_t + \frac{5}{2}|\sigma |$$.

The last condition is only relevant if *A* is an outer region, as it is trivially true for middle and inner regions.

The definition for $S_2(A)$ is also similar to Sect. 6.1, using canonical paths instead of cells. Let $s \in S$ be a site and $\pi _s$ be the path in $\mathcal {Q}$ from the root to $\sigma _s$. Let $\mathcal {P}_{\pi _s}$ be the decomposition of $\pi _s$ into canonical paths as in Lemma 7.2. Let *A* be a region, defined by a canonical path *P*. Then, $s \in S_2(A)$ if

(i) $$P \in \mathcal {P}_{\pi _s}$$ and
(ii) *s* is adjacent in $\mathcal {D}(S)$ to at least one site in $S_1(A)$.

If $\sigma$ is the smallest cell in a canonical path defining a region *A*, then every site $s \in S_2(A)$ lies in $\sigma$, has $r_s < 2|\sigma |$, and intersects at least one site in $S_1(A)$ , satisfying basically the same conditions we had in Sect. 6.1. However, as the definition here is restricted to those canonical paths in $\mathcal {P}_{\pi _s}$, not all sites satisfying the conditions from Sect. 6.1 are considered for inclusion in $S_2(A)$. As we will see below, this suffices to make sure that the proxy graph represents the connectivity, while also ensuring that each site *s* lies in few sets $S_2(A)$.

The graph *H* is now again defined by connecting each region $A \in \mathcal {A}$ to all sites in $s\in S_1(A) \cup S_2(A)$. To show that *H* accurately reflects the connectivity in *D*(*S*), we need the following corollary of Lemma 6.2.

#### Corollary 7.3

Suppose that $d_1 \ge 23$ and $d_2 \ge 8$. Then, for any region $A \in \mathcal {A}$, the sites in $S_1(A)$ form a clique in $\mathcal {D}(S)$.

#### Proof

Recall that the center of the annuli and disks defining *A* is given by the smallest cell of the associated canonical path. A close inspection of the proof for Lemma 6.2 shows that we only use the lower bound on the radii of the sites in $S_1(A)$. As this lower bound is unchanged, all arguments carry over for sites with larger radii. $\square$

#### Lemma 7.4

Two sites are connected in *H* if and only if they are connected in $\mathcal {D}(S)$.

**Fig. 25:**
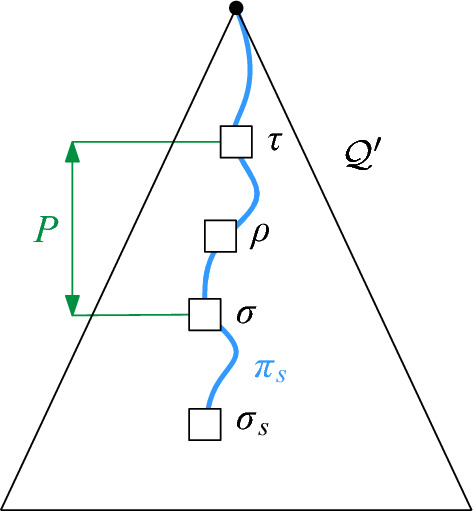
The cell $\sigma _s$ is the smallest cell with $r_s \le 2|\sigma _s|$. The canonical path *P* contains $\rho$

#### Proof

Let $s, t \in S$. If *s* and *t* are connected in *H*, the same argument as in the proof of Lemma 6.3 with Corollary 7.3 instead of Lemma 6.2 applies. The more challenging part is to show that if *s* and *t* are connected in $\mathcal {D}(S)$, they are also connected in *H*. It suffices to show that if *s* and *t* are adjacent in $\mathcal {D}(S)$, they are connected to the same region $A\in \mathcal {A}$. Refer to Fig. 25 for an illustration of the following argument. Assume without loss of generality that $r_s \le r_t$, and consider the neighborhood *N*(*t*) of *t*. By Lemma 6.1, there is a cell $\rho \in N(t)$ that contains *s*. Consider the path $\pi _s$ in $\mathcal {Q}$ from the root to $\sigma _s$. Then, $\rho$ belongs to $\pi _s$. Let $\mathcal {P}_{\pi _s}$ be the decomposition of $\pi _s$ into canonical paths as in Lemma 7.2, and let $P \in \mathcal {P}_{\pi _s}$ be the canonical path containing $\rho$. Let $\sigma$ and $\tau$ be the smallest and the largest cell on *P*, respectively. By the definition of *P*, we have $\sigma _s \subseteq \sigma \subseteq \rho \subseteq \tau$. As *st* is an edge in $\mathcal {D}(S)$, we have $\Vert st \Vert \le r_s + r_t < 2|\sigma | + 2|\tau |$, and thus $\Vert a(\sigma ) t\Vert \le \frac{5}{2}|\sigma | + 2|\tau |$. This implies that *t* lies in a region *A* defined by *P*, and thus $t\in S_1(A)$. As $D_s$ intersects $D_t$, it intersects at least one site in $S_1(A)$. Moreover, *P* is a canonical path in $\mathcal {P}_{\pi _s}$, and thus $s\in S_2(A)$. Thus, *s* and *t* are both connected to *A* in *H*, and the claim follows. $\square$

#### Lemma 7.5

The total size of the binary search trees that define the canonical paths is *O*(*n*) and $\sum _{A\in \mathcal {A}}(|S_1(A)| + |S_2(A)|) = O\left( n\log n\right)$. The proxy graph *H* has *O*(*n*) vertices and $O\left( n\log n\right)$ edges.

#### Proof

As discussed in Sect. 7.1, the compressed quadtree $\mathcal {Q}$ consists of *O*(*n*) cells. Each cell is part of exactly one heavy path, so the total size of the binary search trees that define the canonical paths is *O*(*n*). A balanced binary search tree with *m* leaves has *O*(*m*) inner vertices, and thus, there is a total of *O*(*n*) canonical paths. As each canonical path defines *O*(1) regions, the number of regions, and therefore the number of vertices in *H* follows.

To bound the number of edges, we again count the number of sets $S_1(A)$ and $S_2(A)$ that a single site $t \in S$ can be contained in. Let *P* be a canonical path, let $\sigma$ and $\tau$ be the smallest and largest cell of *P*, and let *A* be an associated region. Suppose that $t \in S_1(A)$. We claim that *N*(*t*) contains a cell that belongs to *P*. By property (ii) in the definition of $S_1(A)$, we have $|\sigma | \le r_t < 2|\tau |$. If $|\sigma |\le r_t < 2|\sigma |$, the claim holds by Lemma 6.1 (we have $\sigma \in N(t)$). Thus, suppose that $2|\sigma | \le r_t < 2|\tau |$, and let $\rho \in \mathcal {G}$ be the cell with $2|\sigma | \le |\rho | \le r_t < 2|\rho |$ and $\sigma \subset \rho \subseteq \tau$. By property (ii) in the definition of $S_1(A)$, we have

$$\begin{aligned} \Vert ta(\sigma ) \Vert \le r_t + \frac{5}{2}|\sigma | \le 2|\rho | + \frac{5}{4}|\rho | = \frac{13}{4}|\rho |, \end{aligned}$$

since $r_t < 2 |\rho |$ and $2|\sigma | \le |\rho |$. Hence, by the triangle inequality $\Vert ta(\rho ) \Vert \le \frac{15}{4}|\rho |$, as $\sigma \subseteq \rho$. By the same considerations as in the proof of Lemma 6.1, it follows that $\rho \in N(t)$, and $\rho$ is in $\mathcal {Q}$ and thus in *P*.

Let $\rho$ be a cell of *N*(*t*), and let *R* be the heavy path containing $\rho$. Then, *t* is considered for the set $S_1(A)$ for all regions that are defined by a canonical path containing $\rho$. These are the $O(\log n)$ paths along the search path for $\rho$ in the binary search tree on the cells of *R*, and thus each site can be part of at most $O(\log n)$ sets $S_1(A)$.

The number of sets $S_2(A)$ that contain a fixed site $t \in S$ is at most the number of canonical paths that partition the path from the root to $\sigma _t$. By Lemma 7.2, there are $O(\log n)$ such paths. This yields $\sum _{A\in \mathcal {A}}(|S_1(A)| + |S_2(A)|) = O\left( n\log n\right)$ as an upper bound for the number of edges. $\square$

### Decremental Data Structure

The approach for the decremental structure is the same as in Sect. 6.2, see Fig. 21. We again store for each region $A \in \mathcal {A}$ the sets $S_1(A)$ and $S_2(A)$ together with an associated RDS. The set *B* for the RDS is again $S_1(A)$, and the set *R* is $S_2(A)$. Furthermore, we store the graph proxy graph *H* in an HLT-structure $\mathcal {H}$.

Both queries and deletions work exactly as in Sect. 6.2, but we repeat them here for completeness. Queries are performed directly to $\mathcal {H}$. To delete of a site *s* from *S*, we first remove all edges incident to *s* from $\mathcal {H}$. Then, *s* is removed from all the sets $S_1(A)$ containing it, as well as from the associated RDS s. The sites *U* reported as revealed by the RDS are then removed from the corresponding sets $S_2(A)$, and the edges *uA*, for $u\in U \setminus S_1(A)$, are removed from $\mathcal {H}$. Finally, the site *s* is removed from all the sets $S_2(A)$ and all corresponding RDS s.

#### Lemma 7.6

The data structure can be preprocessed in $O(n\log ^4 n)$ expected time and requires $O(n \log ^2 n)$ space.

#### Proof

To find the regions $\mathcal {A}$, we first compute the extended compressed quadtree $\mathcal {Q}$. As described in Sect. 7.1, this takes $O(n \log n)$ time.

After that, we need *O*(*n*) additional time to find the heavy paths in $\mathcal {Q}$, by 3 of Lemma 7.1. It also takes *O*(*n*) time to compute the biased binary search trees over the heavy paths by standard techniques [4]. This gives us the set of regions $\mathcal {A}$. To find the sets $S_1(A)$, recall that a site *t* can only belong to the set $S_1(A)$ for a region defined by a canonical path that contains a cell in *N*(*t*). Therefore the sets $S_1(S)$ can be found as follows: for each site $t \in S$, find the cells in *N*(*t*), and for each $\rho \in N(t)$, find the heavy path *R* that contains it. In the biased binary search tree defined on *R*, follow the search path for $\rho$ and for each canonical path along this search path, explicitly find the region containing *t*, and check if the distance condition (condition (iii) for the set $S_1(A)$) holds. A naive implementation of this step takes $O\left( n\log n\right)$ time, which is fast enough for our purposes.

As in Lemma 6.6, we construct an additively weighted Voronoi diagram for each set $S_1(A)$, again assigning the weight $-r_s$ to each site $s\in S$. Recall that the time needed to build a single Voronoi diagram is $O(|S_1(A)| \log n)$ [16, 20, 38]. Since $\sum _{A\in \mathcal {A}} |S_1(A)| = O(n\log n)$ by to Lemma 7.5, the construction of all diagrams takes $O\left( n\log ^2 n \right)$ time. The query time however remains $O(\log n)$ in each diagram, as such a diagram contains at most *O*(*n*) sites. This also limits the temporary space required for all Voronoi diagrams to $O(n \log n)$.

Let $s \in S$. Recall that $\pi _s$ is the path in $\mathcal {Q}$ to $\sigma _s$. To find all sets $S_2(A)$ containing *s*, we obtain the decomposition of $\pi _s$ into canonical paths, and we query the Voronoi diagrams with *s* for all regions defined by these paths. As there are $O\left( \log n\right)$ canonical paths in the decomposition, this takes an additional $O\left( n\log ^2 n\right)$ time for all sites. Inserting the $O\left( n\log n\right)$ edges into $\mathcal {H}$ takes $O\left( \log ^2 n\right)$ amortized time each, for a total of $O\left( n\log ^3 n\right)$.

Again, the step dominating the preprocessing time is the construction of the $\textsf {RDS}$. For a single region $A \in \mathcal {A}$, this is expected $O\left( |S_1(A)|\log ^2 n + |S_2(A)| \log ^3 n \right)$, by Corollary 5.8. We have $\sum _{A\in \mathcal {A}} |S_1(A)| = O(n\log n)$ and $\sum _{A\in \mathcal {A}} |S_2(A)|=O\left( n\log n\right)$ by Lemma 7.5. The claimed preprocessing time follows.

Following Lemma 7.5 again, $\mathcal {H}$ requires $O(n \log n)$ space and both the quadtree and all canonical paths require *O*(*n*) space. Each of the RDS requires $O(|S_1(A)| \log |S_1(A)| + |S_2(A)|)$ space by Corollary 5.8. Using Lemma 7.5 we get that the total space usage for the RDS s is

$$\begin{aligned}&O\left( \sum _{A\in \mathcal {A}} |S_1(A)| \log |S_1(A)| + |S_2(A)| \right) \\&\quad = O\left( \left( \sum _{A\in \mathcal {A}} |S_1(A)| \right) \log n + \left( \sum _{A\in \mathcal {A}} |S_2(A)| \right) \right) \\&\quad = O(n \log ^2 n). \end{aligned}$$

This dominates the overall space usage. $\square$

#### Theorem 7.7

The data structure described above correctly answers connectivity queries in $O(\log n/\log \log n)$ time. It requires $O(n\log ^{5} n)$ overall expected update time , assuming the deletions are oblivious of the internal random choices of the data structure. The data structure requires $O(n \log ^2 n)$ space.

#### Proof

As the only difference with the structure from Theorem 6.7 is the definition of the sets $S_1(A)$ and $S_2(A)$, the correctness follows from Theorem 6.7. The $O(\log n/\log \log n)$ bound for the queries in $\mathcal {H}$ also carries over. The preprocessing takes $O\left( n\log ^4 n\right)$ time by Lemma 7.6. Deletions from $\mathcal {H}$ take $O\left( \log ^2n\right)$ amortized time for each of the $O\left( n\log n\right)$ edges, for an overall time of $O\left( n\log ^3 n\right)$. The time for the sequence of deletions is again dominated by the time needed for the updates in the RDS s. For a region $A \in \mathcal {A}$, let $k_A$ be the number of sites deleted from $S_1(A)$. Then, the time needed by the RDS associated with *A* is $O\left( |S_1(A)| \log ^2 n + k_A\log ^4 n + |S_2(A)|\log ^4 n \right)$ under the assumption of an oblivious adversary, by Corollary 5.8. We have $\sum _{A\in \mathcal {A}} |S_1(A)| = O(n\log n)$, $\sum _{A\in \mathcal {A}} k_A = O(k\log n)$, and $\sum _{A\in \mathcal {A}} |S_2(A)| = O(n\log n)$, following Lemma 7.5. Summing over all $A\in \mathcal {A}$, we get a running time of $O\left( n\log ^5 n\right)$ as claimed. The space usage is unchanged from Lemma 7.6. $\square$

It should be noted that following a similar line of argument as Klost [30], Lemma 7.6 implies an efficient static data structure.

#### Lemma 7.8

There is a static connectivity data structure for disk graphs with $O(n\log ^2n)$ preprocessing time and $O(1)$ query time.

#### Proof

The part in the proof of Lemma 7.6 that computes the proxy graph has a running time of $O(n\log ^2n)$ and yields a graph with the same connected components and only $O(n\log ^2n)$ edges. Using a graph traversal to annotate each vertex with a label of its connected component then takes an additional $O(n\log ^2n)$ time and allows $O(1)$ query time by comparing the labels. $\square$

## Conclusion

We discussed several problems related to dynamic connectivity in disk graphs. First of all, we significantly improved the state of the art for unit disk graphs, by developing data structures tailored to this case. Furthermore, in the general bounded radius ratio case, we were able to improve the dependency on $\Psi$ for updates. We then considered the incremental and decremental setting. For the incremental setting with bounded radius ratio, we gave a data structure with an amortized update time that is logarithmic in $\Psi$ and polylogarithmic in *n* and near constant query time.

In order to obtain a similarly efficient data structure in the decremental setting, we first considered problems related to the lower envelopes of planes and more general two-dimensional surfaces. Using these, we were able to describe a dynamic reveal data structure that is fundamental for our decremental data structure and might be of independent interest. Using the RDS, we were able to give data structures with $O(\log n/\log \log n)$ query time. In the bounded setting, the update time is again logarithmic in $\Psi$ and polylogarithmic in *n*, while for the setting of unbounded radius ratio, we managed to achieve a data structure whose update time depends only on *n*.

For the semi-dynamic data structures described in Sects. 6 and 7, this significantly improves the previously best time bounds that can be derived from the fully dynamic data structure of Chan et al. [13].

There are still several open questions. First of all, our result in the incremental setting only deals with the setting of bounded radius ratio. We are currently working on extending it to the case of general disk graphs. Furthermore, as the incremental and decremental data structures we developed are significantly faster than the fully dynamic data structures, an interesting question would be if similar bounds can be achieved in the fully dynamic setting.

## Figures and Tables

**Fig. 26:**
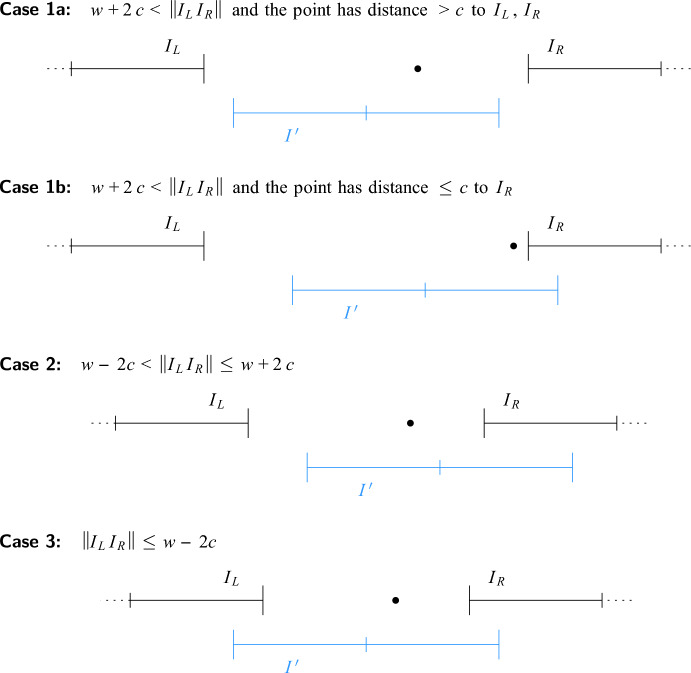
Examples for cases 1–4 with $w = 9c$ and distance $\Vert I_L I_R \Vert$ between $I_L$ and $I_R$. Existing intervals are shown at the top and the newly created intervals at the bottom. The points to cover are drawn as black dots

**Fig. 27:**
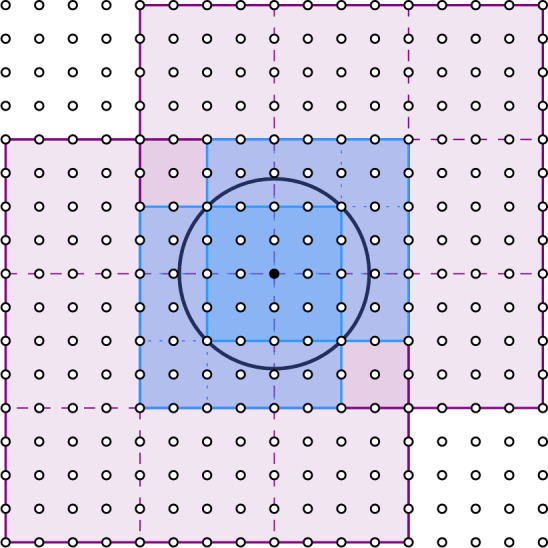
A site $s$ together with the dummy points for constructing the compressed quadtree that includes the $3\times 3$ neighborhood. For the $13\times 13$ neighborhood more dummy points are added

## Implementation on the Real RAM

### Quadforests

Given an arbitrary set of sites $S$ with $\Phi = \max _{s,t\in S} \Vert st\Vert$, the quadtree as defined in Sect. 2.3 has height $O(\log \Phi )$. In Sects. 4 and 6, we developed data structures whose update time depend on the radius ratio $\Psi$ instead. This is done by considering a quadforest instead of a quadtree. The quadforest consists of a set of quadtrees whose root have diameter $\Psi ' = 2^{\lfloor \log _2 \Psi \rfloor }$. Note that given $\Psi$, the value $\Psi '$ can be found with a doubling search in $O(\log \Psi )$ time. In Sects. 3,4 and 6 we assumed that all roots of quadtrees are aligned to a global grid, where the cells of Sect. 3 can be considered as quadtrees with $\Psi \in O(1)$. These assumptions can not easily be satisfied on the real RAM where no floor function is available. In the following, we show how we can achieve the same query, update, and space bounds as with the results of Sects. 3, 4 and 6 without explicitly aligning the quadtree roots to a global grid.

The general idea is to *locally* align quadtree roots instead. To avoid costly rebuilding when sets of roots with incompatible local alignments get too close after several insertions, we allow a small overlap of sets with different alignments. In such an overlap quadtree roots of different sets are handled separately. They may not be aligned to each other and will overlap each other as well. Disks may fall into such an overlap and will then be inserted into quadtrees of all involved sets according to the respective data structures of Sects. 3, 4 and 6, but into at most *O*(1) many. A single set of aligned quadtrees might not contain the full neighborhood of the disk or its cell, but all involved sets together cover the required space.

First, we reduce the problem to a single dimension: we describe how to construct (potentially overlapping) intervals, which allows finding locally aligned quadtree roots in one dimension. Afterwards, we apply this approach for the *x*- and *y*-dimension separately and combine the results to construct the sets of aligned quadtree roots in two dimensions. The approaches are dynamic. Disks can be added or deleted over time, requiring the creation or deletion of new quadtree roots. As a byproduct of handling the dimensions separately, some cells close in one but not two dimensions will be (partially) aligned as well, but that does not cause any problems.

#### Covering Points in One Dimension

In the following we describe how to maintain a set of *O*(*n*) *intervals* of equal size to cover a dynamic set of *n* input points—which will be centers of disks later on—in one dimension. Each $v \in {\mathbb {R}}$ is covered by *O*(1) intervals and the intervals are constructed in such a way that local connectivity of disks is preserved when using them later in Appendix A.1.2. Their size is a multiple of the side length $\Psi '/\sqrt{2}$ of a quadtree root, inducing an alignment of quadtree roots in one dimension in their interior.

Let $c \in O(\Psi )$ be the width of the neighborhood of a disk or quadtree root of maximum size in the respective connectivity data structure described in Sects. 3, 4 and 6. Then, each interval has a width of $w > 8 c \in O(\Psi )$ with *w* a multiple of $\Psi '/\sqrt{2}$. Each interval saves its left and right endpoint and the number of contained points.

During updates of the point set we adjust the set of intervals to maintain the following invariants:

$\varvec{\mathcal {I}}{} {\textbf {1}}$: Each input point is covered by at least one interval.
$\varvec{\mathcal {I}}{} {\textbf {2}}$: Each $v \in {\mathbb {R}}$ is covered by at most two intervals.
$\varvec{\mathcal {I}}{} {\textbf {3}}$: The length of the overlap of two intersecting intervals is at least $c$ and at most $(w-c)/2$.
$\varvec{\mathcal {I}}{} {\textbf {4}}$: Non-intersecting intervals have a distance of at least *c*.^12^

The left and right endpoints of all intervals are maintained in two separate red-black trees [15]. Assuming the invariants hold, the red-black trees allow the retrieval of the up to two covering intervals for a given point in $O(\log n)$ time. Similarly, given a point, the nearest intervals to the left and to the right can be obtained in $O(\log n)$ time. Additionally, a red-black tree containing the current input point set is maintained which contains in each inner node the number of descendant leaves, allowing range counting queries in $O(\log n)$ time.

When a new input point $p$ is added to the point set we might need to create new intervals to fulfill $\mathcal {I}1$. For this, the endpoint trees are queried for intervals containing the new point. If one or two intervals are returned, all invariants are already fulfilled for the updated point set and we just need to increase their point counter by one. If no interval is returned we have to create a new interval to cover the newly added point. For this, the endpoint trees are queried again for the closest interval $I_L$ to the left and $I_R$ to the right. We denote by $\Vert I_L I_R \Vert$ the size of the gap between these disjoint intervals. Then, four different cases can arise, depending on the returned intervals. In the first three cases a new interval will be constructed which leaves a gap at one or both sides, whereas in the last case a gap will be covered with the new interval. The cases are visualized in Fig. 26.

- **Case 1:** $I_L$ **or** $I_R$ **does not exist or** $w + 2c < \Vert I_L I_R\Vert$. This case has two subcases:
  - **Case 1a:** $\Vert p I_L\Vert > c$ **and** $\Vert p I_R\Vert > c$. An arbitrary interval covering the new input point is created which has a distance of at least *c* to the closest intervals.
  - **Case 1b:** $\Vert p I_L\Vert \le c$ **or** $\Vert p I_R\Vert \le c$. Let *I* be the interval with a distance of *c* or less to the new input point. Then, a new interval $I'$ is created such that $I'$ overlaps *I* by *c* and contains the new input point.
- **Case 2:** $w - 2c <\Vert I_L I_R\Vert \le w+2c$. Assume without loss of generality that the distance from the new input point to $I_R$ is at most the distance to $I_L$. Then, we create an interval $I'$ which overlaps $I_R$ by 3*c* on the left side. This new interval must overlap the new input point due to $(w + 2c) / 2 < (w - 3c)$ and has a distance to $I_L$ of more than $(w - 2c) - (w - 3c) = c$.
- **Case 3:** $\Vert I_L I_R\Vert \le w - 2c$. A new interval $I'$ can be created centrally between them. Assuming the distance between $I_L$ and $I_R$ is at least *c*, then $I'$ must overlap $I_L$ and $I_R$ each by at least *c* and by at most $(w - c) / 2$.

Additionally, a range counting query is done on the existing point set, to find all points contained in the new interval. The counter of the new interval is then initialized with the result plus one. Finally, $p$ and the new interval are added to all relevant red-black trees.

When an input point is deleted, we query for all intervals containing it. In all obtained intervals the internal counter is decremented and intervals reaching zero are deleted. Afterwards, the corresponding red-black trees are updated.

The intervals maintained with the construction described fulfill the invariants $\mathcal {I}1$–$\mathcal {I}4$ by induction. Additionally, every interval covers at least one input point. All operations for maintaining the intervals require $O(\log n)$ time per point update, and we maintain three red-black trees and an interval set of size *O*(*n*). Hence, we can conclude:

##### Lemma A.1

We can maintain *O*(*n*) intervals for a dynamic set of points in one dimension which fulfill invariants $\mathcal {I}1$–$\mathcal {I}4$, where *n* is the maximum number of points at any time. To maintain the intervals $O(\log n)$ additional time per point update operation and *O*(*n*) additional space is required.

#### Local Alignment in Two Dimensions

To locally align quadtree roots in two dimensions we construct interval sets according to Lemma A.1 separately for the *x*- and *y*-dimension where the sites form the point set of the lemma. Then, each pair of intervals with one interval from the *x*-set together with one interval from the *y*-set forms a square. Inside each such square, we can for any given point identify a quadtree root aligned to the square’s boundary using binary search in $O(\log \Psi )$ time. This allows us to apply the data structures of Sects. 3, 4 and 6 for each square separately^13^ and a site will be assigned to the data structures of the up to four squares containing it. In this approach we have to take special care to reflect the global connectivity by carefully handling overlapping squares, and we have to make sure to only handle interval pairs whose squares contain at least one site to keep the space requirement low.

To be more precise, we maintain two interval sets of Lemma A.1, one for the *x*-coordinates of the sites and one for their *y*-coordinates. Additionally, we maintain a partial mapping of pairs of *x*- and *y*-intervals to sets of aligned quadtree roots inside the square induced by the respective interval pair. This mapping is implemented as a red-black tree [15] which maps such an interval pair (using e.g.@ the tuple of their left endpoints as key with lexicographical ordering) to a red-black tree that contains the aligned quadtree roots in the square, with the quadtree root’s upper right corner’s coordinate as the key in lexicographical order.

By similar arguments as for the global grid $\mathcal {G}_{\lfloor \log \Psi \rfloor }$, given a query point the corresponding quadtree root in an interval pair can be obtained in $O(\log n)$ time due to the alignment of the quadtree roots. Hence, given a query point, it is possible to obtain all (up to four) quadtree roots containing a point in $O(\log n)$ with the following approach. First, query the red-black trees of the interval endpoints used in Lemma A.1 for both dimensions. Due to $\mathcal {I}2$ of Lemma A.1 at most two intervals are returned in each dimension. Then, for each of the at most four resulting interval pairs in $O(\log n)$ time the corresponding square can be found and as described above with additional time $O(\log n)$ the quadtree root can be identified.

This construction already captures all local connectivity information of the corresponding data structure when inserting sites in all interval pairs which overlap them. In the following we will describe step by step how the data structures from the main part of the paper can be adapted to work with the alignment framework described above. We will refer to the data structures of the main body as the *original* data structures and to the new data structure that is obtained by using the original data structures with the interval as the *adapted* data structures.

##### Lemma A.2

Fix a connectivity data structure $\mathcal {X}$ as described Theorems 3.2, 4.2, 4.6, 6.7, or 6.8. Construct a new data structure by applying the mapping based on interval pairs of Lemma A.1 to a set of sites, constructing an instance of $\mathcal {X}$ for every interval pair that covers at least one site, and inserting all sites into all instances of $\mathcal {X}$ assigned to interval pairs covering it. For this new data structure the following holds:

1. For each pair of sites that are connected by an edge in the disk graph, there exist an interval pair covering both sites.
2. If $\mathcal {X}$ is not the data structure from Theorem 4.6, then the direct connectivity between two sites in the instance of $\mathcal {X}$ of each covering pair is maintained as in the corresponding Lemmas 3.1, 4.1, or 6.3.
3. If $\mathcal {X}$ is the data structure from Theorem 4.6, then in the instance *x* of $\mathcal {X}$ of each covering pair of intervals for two directly connected sites either(a) one of the sites’ disk Minkowski covers the cell of the other site, or (b) they share an edge in the connectivity graph constructed inside *x*.
4. If two sites are not connected in the disk graph, then they are not connected in all instances of $\mathcal {X}$.

##### Proof

Let *c* be the width of a neighborhood in the fixed data structure as defined in Appendix A.1.1. We first consider each dimension and its associated interval set of Lemma A.1 separately.

By $\mathcal {I}1$ of Lemma A.1 each site is covered by at least one interval of the set. We show by contradiction that two sites whose disks intersect are covered by a common interval. Hence, assume that they are not covered by a common interval. Then, two different cases can arise: Either all intervals covering one site do not intersect any interval covering the other site, or at least one interval covering one site intersects an interval covering the other site.

By the definition of the neighborhood in $\mathcal {X}$, the centers of two intersecting disks must have a distance of *less* than *c* in each dimension. Hence, if no intersection between covering intervals exists, invariant $\mathcal {I}4$ of Lemma A.1 is contradicted, as their distance must be at least *c*. If there is an overlap between an interval pair, then at least one interval must contain both disk centers as the overlap is at least of size *c* due to $\mathcal {I}3$ and the length of the intervals is larger than 2*c*, contradicting the assumption. Hence, there exists an interval in each dimension covering the centers of each pair of intersecting disks.

Following this, there exists a pair of an *x*-interval and a *y*-interval, such that their associated two-dimensional area covers both sites. Therefore, if $\mathcal {X}$ is not of Theorem 4.6, then its instance of a covering pair contains all relevant quadtree roots, cells, and other associated parts required to show their connectivity according to Lemma 3.1, 4.1, or 6.3. In case the data structure is of Theorem 4.6 and no site’s disk Minkowski covers the cell of the other site in the instance of $\mathcal {X}$ of the covering pair, then the instance has both disks connected in its internal HLT-structure due to the edge conditions in Sect. 4.2 and the same reasoning as for the other data structures.

Similarly, the instances of $\mathcal {X}$ are constructed according to Theorems 3.2, 4.2, 4.6, 6.7, or 6.8. Hence, two sites in different components of the disk graph are not connected in any instance of $\mathcal {X}$. $\square$

Note that in the construction of instances of Theorem 4.6 for interval pairs the proxy vertices for cells are also determined locally only. In the original, global approach of Theorem 4.6 or in instances of the data structure for other interval pairs there might exist other, possibly larger, sites’ disks which Minkowski cover a site’s cell in a local instance of Theorem 4.6 in Lemma A.2. Due to Lemma A.2 they again share an interval pair, as they have intersecting disks. Hence, multiple candidates for proxy vertices according to Lemma 4.5 can exist in different interval pairs, which requires additional attention later on.

We can use the approach of Lemma A.2 as a basis to adapt the disk connectivity data structures of Sects. 3, 4 and 6 on the real RAM. It remains to bridge the individual data structures with local connectivity into a global connectivity data structure. This can be done by using a global proxy graph *H*, which is shared between all the data structures, instead of many local ones. Additionally, individual subcomponents in the global proxy graph need to be linked appropriately. For the data structures of Theorems 6.7 and 6.8 no further action is required. For the other data structures we add a vertex to *H* for every site and link this vertex to all cells of different interval pairs this site gets assigned to.

##### Lemma A.3

Fix a connectivity data structure $\mathcal {X}$ of Theorems 3.2, 4.2, 4.6, 6.7, or 6.8. Additionally, assume that the interval pairs and instances of $\mathcal {X}$ are constructed as described in Lemma A.2, except that the proxy graph *H* is augmented and shared globally as described above. For this new data structure the following holds:

1. If $\mathcal {X}$ is not the data structure from Theorem 4.6, then two sites are connected in *H* iff they are connected in the disk intersection graph.
2. If $\mathcal {X}$ is the data structure from Theorem 4.6, then at least one pair of proxy cells of two sites is connected in *H* iff the two sites are connected in the disk intersection graph.

##### Proof

First assume that $\mathcal {X}$ is not the data structure from Theorem 4.6. Then, by Lemma A.2 the connectivity of each pair of sites with intersecting disks is reflected in *H* through at least one instance of $\mathcal {X}$, possibly indirectly through the cells they are assigned to. Due to the (potentially added) vertices and edges for each site which link the sites’ cells or regions between different instances of $\mathcal {X}$, a site is connected to all other sites in *H* to which it has an edge in the disk intersection graph. This allows applying the proofs of Lemma 3.1, 4.1, or 6.3.

Now, assume $\mathcal {X}$ is the data structure from Theorem 4.6. Let $D_{s}$, $D_t$ be the disk pair of two connected sites *s*, *t* and let $\{\sigma _i\}$, $\{\tau _j\}$ be the up to four cells of instances of $\mathcal {X}$ the sites are assigned to. Then, let $\{\sigma _i'\}$ be the up to four cells which are proxy vertices of the cells in $\{\sigma _i\}$ and let $\{D_{s_i'}\}$ be the up to four disk which define the proxy vertices, i.e.@ the disks of maximum radius which Minkowski cover the respective $\overline{\sigma _i}$. Every site whose disk contains $D_s$ must be in at least one instance of $\mathcal {X}$ which also contains *s* by Lemma A.2. Hence, at least one disk $D_{s_{k}'}$ must be inclusion maximal. Similarly, at least one disk $D_{t_{\ell }'}$ defining a proxy vertex of a $\tau _j$ must be inclusion maximal.

Similar to the other case, by Lemma A.2 and the bridging edges, the connectivity of all paths of intersecting inclusion maximal disks is preserved in *H*. This allows applying the proof of Lemma 4.5 with $\sigma _{k}'$ and $\tau _{\ell }'$ as the proxy vertices. $\square$

As in the original data structures, this global proxy graph is represented by an instance of Theorem 2.1 when extending Theorem 6.8 and the HLT-structure of Theorem 2.2 for the remaining cases. This directly implies that the query bounds from the original data structures carry over to the adapted data structures. For all adapted data structures except the one from Theorem 4.6 the data structure of the proxy graph is queried directly with the sites. For the adapted data structure derived from the original data structure from Theorem 4.6 all up to four proxy vertices are obtained for both sites according to the instances of the original data structure and then the HLT-structure is queried for connectivity of all up to 16 pairs of proxy vertices.

To insert a new site (unless the original data structure is of Theorem 6.7), we obtain the intervals overlapping the corresponding coordinate of the site from both sets of intervals. When at least one interval per dimension could be found, then the corresponding instances of the original data structure for the up to four pairs are obtained (or initialized if required) and the site is added to them. New aligned quadtree roots can be created and added to the respective red-black trees managing them in $O(\log \Psi + \log n)$ time each, which is always overshadowed by the insertion routine of the respective original data structure.

If instead for at least one dimension no interval could be obtained, then we have to create a new interval as described in Appendix A.1.1. This new interval can overlap previously existing intervals and contain already inserted sites, requiring us to update various data structures. We have to insertion of the sites contained in the new interval into the original data structure assigned to the interval. First, all sites contained in the new interval in the corresponding dimension are obtained with a range query using the red-black tree for points of Appendix A.1.1 in $O(\log n + k)$ time, where *k* is the number of returned sites. Then, for each of the *k* sites, the covering intervals of the other dimension are obtained. For each pair formed by the new interval with an existing interval a new original data structure is initialized, and the pair is added to the red-black tree of pairs. The *k* sites are then added to the new original data structure. This requires in total time for *k* insertions into the instances of the original data structure and $O(k \log n)$ for the remaining updates. The cost for the *k* additional insertions can be charged to the original insertion of the *k* sites, as they occur at most once per dimension due to $\mathcal {I}3$ of Appendix A.1.1.

Afterwards, the new site and the bridging edges are added to $\mathcal {H}$ if required by the original data structure. In total, we have to pay for up to four insertions into instances of the original data structure and the additional operations require $O(\log n)$ time in total.

Deletions work similar as insertions, assuming the original data structure supports deletions. First, the corresponding *x*- and *y*-intervals are obtained in $O(\log n)$ time, then the up to four instances of the original data structure. In each of them the site is deleted. When a quadtree root or even an instance of the data structure for an interval is no longer used, then it is destroyed. In the latter case the corresponding interval pair is removed from the red-black tree of pairs. Additionally, the counter for covered sites of the intervals is decremented. In case one interval’s counter reaches a value of zero, then the interval is deleted as described in Lemma A.1 and removed from all relevant red-black trees. Note that in that case only one or two interval pairs containing that interval can exist, all covering the deleted site. Furthermore, the site vertex and its bridging edges required for Lemma A.3 are removed from $\mathcal {H}$ if they were introduced, depending on the original data structure. All steps require the time for at most four deletions in the instances of the original data structures, four deletions in $\mathcal {H}$, and additional $O(\log n)$ overhead for the remaining work. Hence, asymptotically the run time is unchanged.

Although we require a relatively large amount of auxiliary data structures, all of them require *O*(*n*) additional storage. Similarly, we require *O*(*n*) space for the interval data structures due to Lemma A.1. The number of additional vertices and edges in comparison to the original data structures is also bound by *O*(*n*). This results in an asymptotic space usage identical to the respective original data structure.

Combining everything above, we can conclude the following:

##### Theorem A.4

(Semi-)dynamic disk connectivity can be implemented with the bounds of Theorems 3.2, 4.2, 4.6, 6.7 and 6.8 on a real RAM.

### Compressed Quadtrees with Neighbor Pointers

In this section we describe how the compressed quadtrees can be adapted in order to allow the same running times as given in Sect. 7 in the real RAM model without using the floor function. The relevant operations that are needed for the data structure in Sect. 7 are constructing a compressed quadtree that contains the cells in $N(s)$ for any given $s\in S$ and efficiently finding the neighbors on the same level of a given cell.

The algorithm of Buchin et al. [7] to construct a compressed quadtree from a set of point sites takes $O(n\log n)$ time on the real RAM without rounding. It achieves this running time with the disadvantage of not having all cells aligned to the hierarchical grid. On a high level, Buchin et al. start building a non-compressed quadtree and whenever splitting a cell only lead to one new non-empty cell for $c$ iterations, a compressed vertex is added and a new bounding box is computed for the vertices in the cell. This process is then continued recursively.

This section will focus on how the data structure in Sect. 7 can be easily adapted to work with a non-aligned grid. We still want to have a cell $\sigma _s$ for each site $s\in S$ with $s\in \sigma$ and $|\sigma _s|\le r_s < 2 |\sigma _s|$ but relax the definition in so far that $\sigma _s$ does not have to be part of a global hierarchical grid but is the cell of the (not necessary aligned) compressed quadtree on the sites with this property. Below we will describe a process to guarantee that such a cell exists. We also relax the notion of the neighborhood $N(s)$. We will not use a $15\times 15$ subgrid on the same level as $|\sigma _s|$, but instead use all cells of the compressed quadtree that would intersect this $15\times 15$ subgrid and whose diameter lies in the interval $\left[ |\sigma _s|, 2|\sigma _s|\right)$. The size of the neighborhood is still $O(1)$.

#### Observation A.5

Given a site $s$, the cells in $N(s)$ are disjoint.

#### Lemma A.6

Let $S\subseteq \mathbb {R}^2$ be a set of sites with associated radii. A compressed quadtree that contains a suitable neighborhood $N(s)$ for every $s\in S$ can be constructed in $O(n\log n)$ time on a real RAM. Furthermore, the constructed quadtree allows to return the neighborhood of a cell in $O(1)$ time, assuming a pointer to the cell is given.

#### Proof

We use the algorithm of Buchin et al. [7] to construct the quadtree. The algorithm expects a set of points as its input, so we construct a point set that makes sure that a cell $\sigma _s$ and its neighborhood are contained in the quadtree.

For this we take all sites $s\in S$ and surround them with a $61\times 61$ grid of points, where the vertical and horizontal distance between neighboring points is $\frac{r_s}{2\sqrt{2}}$. The distance between these points enforces a cell $\sigma _s$ with $s\in \sigma _s$ and $|\sigma _s|\le r_s < 2|\sigma _s|$ together with the cells in its $15\times 15$ neighborhood to be split and thus be present in the compressed quadtree, see Fig. 27. As these are $O(n)$ points, the construction takes $O(n\log n)$ time.

Now we describe how to find the neighborhood. This can be done by constructing a set of pointers in a traversal of the constructed quadtree. Here the main observation is, that the neighborhood of a given cell is fully contained in the neighborhood of its parent. So when traversing the quadtree cells in increasing size, one can always traverse the neighborhood of the parent in order to find the neighborhood of any cell. This traversal can be done using a priority queue in $O(n\log n)$ overall time. After this traversal, the stored neighborhood of a cell can be returned in $O(1)$ time. $\square$

## Footnotes

1 Actually, Chan et al. [13] claim an amortized expected update time of $O(\log ^{10} n)$. However, the underlying data structures have been improved since their paper was published [12]. With this, the approach yields the stated improved running time; see Sect. 3 for more details.

2 The original paper claims an amortized expected update time of $O(\Psi ^2 \lambda _6(\log n) \log ^{9}n)$, but recent improvements in the underlying data structure [31] lead to the better bound.

3 We emphasize that the diameter of $\sigma$ is defined with respect to the closure, i.e., $\sigma$ has diameter $2^i$, but any pair of points that lie in $\sigma$ have distance strictly less than $2^i$.

4 Usually, quadtrees are defined for planar point sets and not for cells in a hierarchical grid [16]. However, for our purposes, it is more convenient to work at the cell level, since sites that are close together form cliques in $\mathcal {D}(S)$ and can thus be represented by a single cell that contains them.

5 At the time of Chan et al.’s original paper [13], Chan’s improvement [12] was not available yet. Instead, Chan et al. [13] use a result by Eppstein that reduces the DBCP problem to several instances of the DNN problem for points in the plane [18], with another $O(\log ^2 n)$ factor as overhead in the update time. Using Chan’s original DNN structure [11] with amortized expected update time $O(\log ^6 n)$, this leads to a total amortized expected update time of $O(\log ^{10} n)$.

6 Actually, instead of the MBM s, we could also use the dynamic bichromatic closest pair (DBCP) structure for general distance functions of Kaplan et al. [27]. It achieves the same time bounds, but it is slightly more complicated. Hence, we prefer to stay with MBM s in our presentation.

7 Note that this differs from Sect. 3, where sites with radius 1 are assigned to cells of diameter 2.

8 Note that unlike in Sect. 4.1, the neighborhoods depend on the site *s* and not just the cell $\sigma$ that *s* is assigned to.

9 Note that the non-deleted planes below *q* could still be marked as pruned, and hence cannot be used for the sampling. However, we will see below that this difficulty can be easily dealt with.

10 In the proof of Kaplan et al.@, the constant $b'$ has to be chosen larger than originally (e.g., $b' \ge 8 \alpha b''$), as purging a prism $\tau$ releases at least $(b'/4 \alpha - b'') \left| {{\,\textrm{CL}\,}}(\tau ) \right| \log N$ credits when changing *f* to $f'$.

11 Here, in the decremental setting, $\mathfrak {N} = \{ \sigma _s \mid s \in S\}$ would already be sufficient, as the omitted cells would never have a nonempty $S_2(A)$ for their regions *A*.
